# ﻿Taxonomy, phylogeny, and bioactive potential of Xylariales (Sordariomycetes, Ascomycota) from Thailand: novel species discovery, new host and geographical records, and antibacterial properties

**DOI:** 10.3897/mycokeys.120.155915

**Published:** 2025-07-29

**Authors:** Achala R. Rathnayaka, K. W. Thilini Chethana, Areerat Manowong, Amuhenage T. Bhagya, Hsan Win, Zaw L. Tun, Ausana Mapook, Kevin D. Hyde

**Affiliations:** 1 School of Science, Mae Fah Luang University, Chiang Rai 57100, Thailand; 2 Center of Excellence in Fungal Research, Mae Fah Luang University, Chiang Rai 57100, Thailand; 3 Key Laboratory of Phytochemistry and Natural Medicines, Kunming Institute of Botany, Chinese Academy of Sciences, Kunming 650201, China; 4 Department of Plant Pathology, College of Agriculture, Guizhou University, Guiyang Guizhou 550025, China

**Keywords:** 5 new species, Ascomycota, morphology, multi-gene phylogeny, preliminary screening test

## Abstract

Xylariales (Sordariomycetes, Ascomycota) comprise a wide range of species that exhibit considerable variation in stromatic characteristics, including conspicuous to inconspicuous perithecia and unitunicate asci. Most known species are endophytes and saprobes, recognized for producing secondary metabolites of fundamental importance in the pharmaceutical and chemical industries. The main objectives of this study were to identify novel species, document new host and geographical records within the families Diatrypaceae, Hypoxylaceae, and Xylariaceae in northern and central Thailand, and explore the bioactive properties of secondary metabolites produced by selected Xylariales species. Taxa were identified through morphological examination, supported by phylogenetic analyses using maximum likelihood and Bayesian inference based on LSU, ITS, *rpb2*, and *β-tub* gene sequences. These taxa are accompanied by comprehensive descriptions and illustrations. Xylariales cultures were screened for preliminary antibacterial activity against the bacterial pathogens *Bacillussubtilis* (Gram-positive) and *Escherichiacoli* (Gram-negative). Based on the screening results, two newly introduced species (*Annulohypoxylonchiangraiense* and *Hypoxylonthailandicum*) and two known species (*Xylariachrysanthum* and *Daldiniaeschscholtzii*), which exhibited antibacterial activity, were selected for secondary metabolite extraction. Crude extracts from these isolates were chemically profiled using high-performance liquid chromatography (HPLC) and Q-TOF analyses, revealing a variety of potential compounds. The present study enhances our understanding of the taxonomic diversity and bioactive potential of Xylariales by introducing five new species, reporting nine new host records—including one new geographical record—and evaluating the bioactive properties of selected Xylariales cultures.

## ﻿Introduction

Xylariales was established by Nannfeldt (1932) with Xylariaceae as the type family, along with Diatrypaceae, Hypocreaceae, Hyponectriaceae, Lasiosphaeriaceae, and Polystigmataceae. Initially, Xylariales species were mainly classified based on morphological characters (Hawksworth et al. 1995). However, the incorporation of molecular data has since diversified the classification criteria (Eriksson et al. 2003). Smith et al. (2003) conducted the first multi-gene analysis and identified seven families within this order. Lumbsch and Huhndorf (2010) listed six families, while Senanayake et al. (2015) included eleven families in Xylariales. Based on morphology and multi-gene analyses, Hyde et al. (2020a) accepted fifteen families in Xylariales. Fasciatisporaceae was introduced by Hyde et al. (2020a) to accommodate *Fasciatispora* within Xylariales, which comprises twenty families as outlined by Wijayawardene et al. (2022). However, some taxa in this order are considered genera *incertae sedis* due to the uncertainty of their taxonomic positions (Hyde et al. 2020a). Currently, Xylariales is considered the second-largest order in Xylariomycetidae, with 160 genera placed at the family level and 52 in genera *incertae sedis* (Hyde et al. 2020b; Phukhamsakda et al. 2020; Konta et al. 2021). Recently, Samarakoon et al. (2022) revised the taxonomy of xylarialean taxa based on morphology coupled with molecular phylogeny and accepted 57 genera *incertae sedis* in Xylariales. Hyde et al. (2024) and Thiyagaraja et al. (2025) included 22 families, expanding the taxonomic framework of Xylariales.

Xylariales species possess both conspicuous and inconspicuous fruiting bodies and are unitunicate, perithecial ascomycetes (Smith et al. 2003). This is a diverse group of fungi with distinct stromatic characteristics, which play a major role in generic and family-level classification (Jayawardena et al. 2022; Phukhamsakda et al. 2022; Samarakoon et al. 2022; 2023). In some xylarialean taxa, a distinct morphological character called the clypeus is present. It consists of stromatic tissues or melanized hyphae that develop above partially submerged or immersed ascomata. The clypeus forms a shield-shaped structure with variable development (Læssøe and Spooner 1993). Xylariales species occur as saprobes and endophytes in temperate, subtropical, and tropical regions worldwide, associated with wood, fallen fruits or seeds, fallen leaves or petioles, and termite nests (U’Ren et al. 2016; Fournier et al. 2018).

Within Xylariales, Xylariaceae and Hypoxylaceae are the most well-known families, producing secondary metabolites significant to the pharmaceutical and chemical industries. Some endophytic Xylariales species, such as *Hypoxylonrubiginosum* and *Xylariacf.curta*, are used for biological control due to their strong antagonistic effects against fungal and other pathogens (Halecker et al. 2020; Becker and Stadler 2021; Chen et al. 2024). Xylariaceae is considered one of the largest and most diverse families in Xylariales and comprises 42 genera and nearly 852 species (Hyde et al. 2024). *Xylaria*, the largest genus in Xylariaceae, was introduced with *X.hypoxylon* as the type species (Peršoh et al. 2009). Most Xylariaceae species are endophytes or saprobes, although a few have been reported as pathogens (Rogers 2000; Okane et al. 2008; Stadler et al. 2013; Husbands et al. 2018; Pourmoghaddam et al. 2022). These species can be found in wood, leaves, fruits, seeds, dung, and soil (Karimi et al. 2024). The family is characterized by embedded, well-developed ascomata and dark-colored stromata, which may be reduced or absent. The asci are 8-spored, unitunicate, and cylindrical and may possess an amyloid apical ring. The ascospores are pigmented and exhibit germ slits or pores (Rogers 2000). The asexual morph is characterized by holoblastic conidia and sympodially or occasionally percurrently proliferating conidiogenous cells (Rogers 2000).

Hypoxylaceae comprises 19 genera and approximately 422 species (Hyde et al. 2024). Within this family, *Annulohypoxylon* and *Hypoxylon* are the largest genera, comprising 69 and 235 species, respectively (Karimi et al. 2024). Hypoxylaceae species have a cosmopolitan distribution and occur as saprobes, endophytes, and pathogens (Reyes et al. 2024). This family is characterized by erect, glomerate, pulvinate, discoid, effused-pulvinate, hemispherical, spherical, or peltate stromata, which may be solitary or confluent, brightly colored, dark or black, pruinose, or smooth. Some stromata can produce extractable pigments visible in 10% KOH (Reyes et al. 2024). The perithecia are spherical, obovoid, or tubular, with spherical, umbilicate, or papillate ostioles, with or without discs formed by dehiscence of the surrounding tissue. A nodulisporium-like asexual state, distinguishable from that in Xylariaceae, occurs in Hypoxylaceae species (Reyes et al. 2024).

In Xylariales, diatrypaceous taxa are abundant and widely distributed (Ma et al. 2023). Diatrypaceae species occur as saprobes, endophytes, and pathogens on a wide range of crops and woody plants (Vasilyeva and Ma 2014; Konta et al. 2020a; Samarakoon et al. 2022; Li et al. 2023). Members of this family can produce extracellular, ligninolytic enzymes that degrade plant cell walls, playing an important role in wood decomposition (Bucher et al. 2004; Trouillas et al. 2011; Mehrabi et al. 2015). Diatrypaceae species are characterized by eustromatic or pseudostromatic, black or dark brown, erumpent to immersed, and occasionally superficial stromata. Ostioles are present in the perithecia. The asci are 8-spored or polysporous, occasionally with one or two spores, and are unitunicate. The ascospores are ellipsoidal, globose, filiform, or allantoid, and hyaline to light brown (Senanayake et al. 2015). The asexual morph is characterized by acervulus-like subcortical, erumpent conidiomata. The conidia are hyaline, filiform, curved, or rarely straight (Senanayake et al. 2015; Wijayawardene et al. 2017; Phukhamsakda et al. 2022). Currently, Diatrypaceae comprises 31 genera (Hyde et al. 2024). Given the widespread occurrence and varied lifestyles of Xylariales species, further taxonomic and ecological studies are essential.

Within Xylariales, the families Hypoxylaceae and Xylariaceae are the most prolific producers of secondary metabolites (Becker and Stadler 2021). Numerous unique secondary metabolites have been discovered from the stromata and mycelial cultures of Xylariales fungi, many of which have potential pharmaceutical and agrochemical applications (Becker and Stadler 2021). For example, *Kretzschmariazonata*, a plant pathogenic fungus, produces various enzymes such as β-glucosidases, endoglucanases, hemicellulases, pectinases, and xylanases (da Luz Morales et al. 2021). In Xylariaceae, *Xylaria* species have yielded a wide range of bioactive compounds, including alkaloids, aromatic compounds, cytochalasins, polyketides, and terpenoids. These compounds exhibit diverse biological activities, including antibacterial, antifungal, anticancer, antimalarial, anti-inflammatory, and α-glucosidase inhibitory activities (Chen et al. 2024). Additionally, Whalley and Edwards (1995, 1998) reported that many chemical metabolites produced by Xylariaceae cultures exhibit a degree of genus specificity.

Beyond their bioactivity, chemotaxonomic data support the segregation of genera within Hypoxylaceae, such as *Annulohypoxylon*, *Hypomontagnella*, *Hypoxylon*, and *Jackrogersella* (Becker et al. 2020; Cedeño-Sanchez et al. 2024). Species in Hypoxylaceae are known to produce a diverse array of secondary metabolites, particularly azaphilones and binaphthalenes (Kuhnert et al. 2021). To date, more than 200 metabolites with various bioactivities have been identified from *Hypoxylon* species, including hypoxyloamide, 8-methoxynaphthalene-1,7-diol, and hypoxylonol, which exhibit antimicrobial and anticancer activities (Tan and Zou 2001; Cheng et al. 2020). The growing focus on chemotaxonomic studies of Xylariales species is likely to uncover many previously unknown secondary metabolites with significant bioactive potential.

The main objectives of this study are to introduce five novel taxa, report nine new host records including one new geographical record of species belonging to Diatrypaceae, Hypoxylaceae, and Xylariaceae in Xylariales, and to investigate bioactive compounds produced by cultures of selected Xylariales species. Morphological characteristics and multi-gene phylogenetic analyses using maximum likelihood (ML) and Bayesian inference (BI) confirmed the phylogenetic placement of the studied fungal taxa within the order Xylariales. Preliminary antibacterial screening was conducted for selected species, including newly described taxa in *Annulohypoxylon* and *Hypoxylon*. Additionally, bioactive compounds from the cultures of these species were analyzed using HPLC/Q-TOF techniques.

## ﻿Materials and methods

### ﻿Specimen collections, morphological studies, and isolations

Specimens (dead wood) were collected during March–July 2024 in Thailand. Samples were enclosed in zip-lock plastic bags and transported to the laboratory. Observations followed the methodology of Senanayake et al. (2020). Morphological characteristics were examined using a LEICA EZ4 stereomicroscope (Leica Microsystems, Germany) and an AXIOSKOP 2 PLUS compound microscope (Carl Zeiss Microscopy, Germany). Microscopic structures were photographed with a Canon 550D digital camera mounted on the microscope. Melzer’s reagent, Congo red, and Indian ink were used as needed. Measurements were made using ZEN2 (Blue Edition) software and calculated with the Tarosoft® Image Framework program. Figures and photo plates were assembled using Adobe Photoshop CS3 Extended version 10.0 (Adobe Systems, USA).

Single-spore isolations were carried out on potato dextrose agar (PDA) following Senanayake et al. (2020). Herbarium specimens were deposited at the Mae Fah Luang University Herbarium (MFLU), Chiang Rai, Thailand, and living cultures were preserved in the Mae Fah Luang University Culture Collection (MFLUCC). For newly introduced taxa, Faces of Fungi numbers were obtained according to Jayasiri et al. (2015), and Index Fungorum numbers were registered via Index Fungorum (2025). All data generated in this study were deposited in the Greater Mekong Subregion Fungal Database (Chaiwan et al. 2021).

### ﻿DNA extraction, PCR amplification, and sequencing

Genomic DNA was extracted from 50–100 mg of fungal mycelium using the E.Z.N.A Fungal DNA Mini Kit (D3390-02, Omega Bio-Tek, USA) according to the manufacturer’s instructions. Extracted DNA was stored at 4 °C for short-term use and at -20 °C for long-term storage. Polymerase chain reactions (PCR) were performed for the large subunit rDNA (LSU), the internal transcribed spacer region (ITS), *β-tubulin* (*β-tub*), and RNA polymerase II second largest subunit (*rpb2*) gene regions as described in previous studies (Thiyagaraja et al. 2019; Konta et al. 2020a; Karimi et al. 2023).

The LSU, ITS, *β-tub*, and *rpb2* gene regions were amplified using the primers LR0R/LR5 (Vilgalys and Hester 1990; Rehner and Samuels 1994), ITS4/ITS5 (White et al. 1990), T1/T22 (O’Donnell and Cigelnik 1997), Bt2a/Bt2b (Glass and Donaldson 1995), and fRPB2-5f/fRPB2-7cR (Liu et al. 1999), respectively. PCR reactions were carried out in a final volume of 50 μl, consisting of 25 μl of 2× Power Taq PCR Master Mix, 1 μl of each forward and reverse primer, 2 μl of genomic DNA, and 21 μl of deionized water. The PCR products were visualized on 1.5% agarose gels, stained with 4S Green Stain, and subsequently sequenced at SolGent Co., Ltd. (South Korea). Newly generated nucleotide sequences were deposited in GenBank (Table 1).

**Table 1.** Taxa used in the phylogenetic analysis and their GenBank accession numbers. New isolates generated in this study are in bold, and type strains are indicated in superscript ‘^T^’.

Species	Strain no.	GenBank accession numbers				References
		LSU	ITS	*rpb*2	*β-tub*	
*Allocryptovalsacastaneicola*	CFCC52432^T^	–	NR 177157	–	NA	Zhu et al. (2021)
*A.cryptovalsoidea*	HVFIG02^T^	–	HQ692573	–	HQ692524	Trouillas et al. (2011)
*A.elaeidis*	MFLUCC 15-0707	–	MN308410	–	MN340296	Konta et al. (2020b)
*A.polyspora*	MFLUCC 17-0364 ^T^	–	MF959500	–	MG334556	Senwanna et al. (2017)
*A.sichuanensis*	HKAS 107017^T^	–	NR 175673	–	MW775592	Samarakoon et al. (2022)
*A.xishuangbanica*	GMB0417	–	OP935176	–	OP938739	Li et al. (2023)
*A.xishuangbanica*	KUMCC 21-0830^T^	–	ON041128	–	ON081498	Maharachchikumbura et al. (2022)
*Allodiatrypealbelloscutata*	IFRD 9100^T^	–	OK257020	–	N/A	Li et al. (2022a)
*A.albelloscutata*	KUNCC 23-15531	–	PP584727	–	N/A	Dissanayake et al. (2024)
*A.arengae*	MFLUCC 15-0713 ^T^	–	MN308411	–	MN340297	Konta et al. (2020b)
*A.elaeidicola*	MFLUCC 15-0737^T^	–	MN308415	–	MN340299	Konta et al. (2020b)
*A.elaeidis*	MFLUCC 15-0708b	–	MN308413	–	N/A	Konta et al. (2020b)
*Alloeutypaflavovirens*	CBS 272. 87	–	AJ302457	–	DQ006959	Rolshausen et al. (2006)
*A.milinensis*	FCATAS 4309^T^	–	OP538689	–	OP557595	Ma et al. (2023)
*A.milinensis*	FCATAS 4382	–	OP538690	–	OP557596	Ma et al. (2023)
*Annulohypoxylonalbidiscum*	MFLUCC 15-0645^T^	N/A	KU852741	N/A	N/A	Li et al. (2016)
*A.annulatum*	DSM:107931	MK287546	MK287534	MK287559	MK287572	Sir et al. (2019)
*A.archeri*	SFC20220920-G065	N/A	MW497176	N/A	N/A	Lee et al. (2023)
*A.areolatum*	MFLUCC 14-1233^T^	N/A	NR_153554	N/A	KX376344	Kuhnert et al. (2017)
*A.atroroseum*	EK14006	N/A	KP401581	N/A	KP401588	Sir et al. (2015)
*A.bahnphadengense*	STMA 13115	N/A	KX376338	N/A	KX376347	Kuhnert et al. (2017)
***A.bahnphadengense***	**MFLUCC 24-0608**	**PQ860996**	**PQ861001**	**PQ878514**	**N/A**	This study
*A.bovei*	PDD:119612	N/A	PP965769	PP963634	PP963581	Johansen and Johnston (2011)
***A.chiangraiense***	**MFLUCC 24-0606^T^**	**PV468228**	**PP988691**	**PV426649**	**PV426648**	This study
*A.cohaerens*	CBS 119126	N/A	KC477233	N/A	N/A	Stadler et al. (2013)
*A.crowfoothodgkiniae*	BRIP 72527h^T^	OP598059	OP599617	N/A	N/A	Tan and Shivas (2022a)
***A.crowfoothodgkiniae***	**MFLUCC 25-0023**	**PQ860994**	**PQ860999**	**N/A**	**PV443841**	This study
*A.fulvum*	MUCL 54617	N/A	KX376336	N/A	KX376355	Kuhnert et al. (2017)
*A.kwolekiae*	BRIP 72473a^T^	PP348248	NR_182614	N/A	N/A	Tan and Shivas (2022a)
*A.lancangensis*	ZHKUCC 23-0104^T^	OR224539	OR224542	N/A	N/A	Liu et al. (2025)
*A.leptascum*	MFLUCC 13-0587	N/A	KU604576	N/A	KU604580	Kuhnert et al. (2017)
*A.macrosporum*	ST2584	N/A	DQ322097	N/A	N/A	Suwannasai (2005)
*A.maeteangense*	CBS 123835	N/A	KX376322	N/A	N/A	Kuhnert et al. (2017)
*A.massivum*	MUCL 47218^T^	N/A	AM749938	N/A	KC977276	Bitzer et al. (2008); Kuhnert et al. (2014a)
*A.michelianum*	CBS 119993	KY610423	KX376320	N/A	KX271239	Kuhnert et al. (2017)
*A.microdiscum*	YMJ 90080807	N/A	EF026137	N/A	AY951660	Hsieh et al. (2010)
*A.minutellum*	CBS 119015	N/A	JX658447	N/A	KX271240	Kuhnert et al. (2017)
*A.moriforme*	CBS 123579	KY610425	KX376321	KY624289	KX271261	Kuhnert et al. (2017)
*A.nitens*	MFLUCC 12-0823	KJ934992	KJ934991	KJ934994	KJ934993	Daranagama et al. (2015)
*A.nouraguense*	MUCL 54607	N/A	KX376335	N/A	KX376348	Kuhnert et al. (2017)
*A.palmicola*	MFLUCC 11-0020^T^	NG 071242	KT369002	N/A	N/A	Ariyawansa et al. (2015)
*A.purpureonitens*	ST2448	N/A	DQ223756	N/A	N/A	Suwannasai (2005)
***A.purpureonitens***	**MFLUCC 24-0609**	**PQ860997**	**PQ861002**	**PQ878515**	**N/A**	This study
*A.purpureopigmentum*	MUCL:54616	N/A	KC968942	N/A	KC977306	Kuhnert et al. (2014a)
*A.squamulosum*	YMJ 90081905	N/A	EF026139	N/A	AY951665	Hsieh et al. (2010)
*A.spougei*	SWUF09-032	N/A	OL449769	PP598908	PP598940	Pimjuk et al. (2022)
***A.spougei***	**MFLUCC 24-0607**	**PQ860995**	**PQ861000**	**PQ878513**	**N/A**	This study
*A.stygium*	MFLUCC 12-0826^T^	KJ940869	KJ940870	KJ940868	KJ940867	Daranagama et al. (2015)
*A.subnitens*	MUCL 54594	PP348087	KX376333	N/A	N/A	Kuhnert et al. (2017)
*A.substygium*	MUCL 51708	N/A	KC968915	N/A	KC977285	Kuhnert et al. (2017)
*A.substygium*	STMA 14066	N/A	KU604575	N/A	KU159526	Sir et al. (2016)
*A.thailandicum*	MFLUCC 13-0118^T^	NG_228734	NR_153529	N/A	KX376349	Liu et al. (2015)
*A.truncatum*	CBS 140778^T^	N/A	NR_153580	KY624277	KX376352	Kuhnert et al. (2017)
*A.truncatum*	DSM: 107925	MK287543	MK287531	MK287556	MK287569	Sir et al. (2019)
*A.urceolatum*	EK14014	N/A	KP401582	N/A	KP401589	Sir et al. (2015)
*A.violaceopigmentum*	MFLUCC 14-1225	N/A	KX376326	N/A	KX376343	Kuhnert et al. (2017)
***A.violaceopigmentum***	**MFLUCC 24-0610**	**PQ860998**	**PQ861003**	**PQ878516**	**N/A**	This study
*A.viridistratum*	MFLUCC 14-1224	PP356919	KX376325	PP598916	KX376342	Kuhnert et al. (2017)
*A.yungensis*	STMA 14046	N/A	KX376323	N/A	KX376340	Kuhnert et al. (2017)
*Anthostomadecipiens*	IPV-FW349	–	AM399021	–	AM920693	Luque et al. (2012)
*A.decipiens*	JL567	–	JN975370	–	JN975407	Luque et al. (2012)
*Astrocystisbambusae*	GMB0700	–	PP146578	–	PP209113	Li et al. (2024)
*A.bambusae*	HAST 89021904	–	GU322449	GQ844836	GQ495942	Hsieh et al. (2010)
***A.bambusae***	**MFLUCC 25-0022**	–	**PQ844814**	**PQ855789**	**PQ855790**	This study
*A.cocoes*	GMB0037	–	MW732441	MW755333	MW755339	Wu et al. (2021)
*A.concavispora*	MFLUCC 14-0174	–	KP297404	KP340532	KP406615	Daranagama et al. (2015)
*A.dinghuensis*	GMB0704	–	PP133237	PP198070	PP197684	Li et al. (2024)
*A.dinghuensis*	GMB0783	–	PP133238	PP198071	PP197683	Li et al. (2024)
*A.guizhouensis*	GMB0705	–	PP133239	PP198072	PP197682	Li et al. (2024)
*A.guizhouensis*	GMB0796	–	PP133240	PP198073	PP197681	Li et al. (2024)
*A.heterocyclae*	GMB0706	–	PP153340	PP198074	PP197680	Li et al. (2024)
*A.heterocyclae*	GMB0788	–	PP153341	PP198075	PP197679	Li et al. (2024)
*A.mirabilis*	HAST 94070803	–	GU322448	GQ844835	GQ495941	Hsieh et al. (2010)
*A.multiloculata*	GMB0033	–	MW732439	MW755330	MW755336	Wu et al. (2021)
*A.pseudomirabilis*	GMB0122^T^	–	ON471845	ON462000	ON461996	Li et al. (2022b)
*A.sichuanensis*	GMB0708	–	PP153343	PP198076	PP197678	Li et al. (2024)
*A.sichuanensis*	GMB0709	–	PP153342	N/A	PP197677	Li et al. (2024)
*A.sublimbata*	HAST 89032207	–	GU322447	GQ844834	GQ495940	Hsieh et al. (2010)
*A.tessellati*	GMB0120^T^	–	ON471849	ON462003	ON461994	Li et al. (2022b)
*Biscogniauxiapetrensis*	HKAS 102388	MW240544	MW240615	MW342619	MW775576	Samarakoon et al. (2022)
*Collodisculabambusae*	GZUH0102	–	KP054279	KP276675	KP276674	Li et al. (2015)
*C.baoshanensis*	GMB0720	–	PP153344	PP198077	PP197676	Li et al. (2024)
*C.baoshanensis*	GMB0795	–	PP153345	PP198078	PP197675	Li et al. (2024)
*C.fangjingshanensis*	GZUH0109^T^	–	KR002590	KR002591	KR002592	Li et al. (2015)
*C.japonica*	CBS:124266	–	JF440974	KY624273	KY624316	Jaklitsch and Voglmayr (2012)
*C.lancangjiangensis*	GMB0030^T^	–	MW732442	N/A	MW755343	Wu et al. (2021)
*C.leigongshanensis*	GZUH0107	–	KP054281	KR002588	KR002587	Li et al. (2015)
*C.quadrangularis*	GMB0722	–	PP153347	–	PP197674	Li et al. (2024)
*C.quadrangularis*	GMB0784	–	PP153346	–	PP197673	Li et al. (2024)
*C.tubulosa*	GACP QR0111	–	MN017302	MN018403	MN018405	Xie et al. (2020)
*Cryptosphaeriaeunomia*	CBS 216.87	–	AJJ02417	–	N/A	Acero et al. (2004)
*C.eunomia*	CBS 223.87	–	AJ302421	–	N/A	Acero et al. (2004)
*C.ligniota*	CBS 273. 87^T^	–	KT425233	–	KT425168	Acero et al. (2004)
*C.pullmanensis*	ATCC 52655	–	KT425235	–	KT425170	Trouillas et al. (2015)
*C.subcutanea*	CBS 240. 87^T^	–	NR_138412	–	KT425167	Trouillas et al. (2015)
*C.subcutanea*	DSUB100A	–	KT425189	–	KT425124	Trouillas et al. (2015)
*Cryptovalsaampelina*	A001	–	GQ293901	–	GQ293972	Trouillas et al. (2010)
*C.ampelina*	DRO101	–	GQ293902	–	GQ293982	Trouillas et al. (2010)
*Daldiniabambusicola*	CBS 122872^T^	MH874769	MH863245	KY624241	N/A	Vu et al. (2019)
*D.childiae*	CBS 122881^T^	MH874773	NR_172249	KU684290	KU684129	U’Ren et al. (2016)
*D.concentrica*	CBS 113277	KY610434	JX658475	KY624243	KC977274	Wendt et al. (2018)
*D.dennisii*	CBS 114741	KY610435	JX658477	KY624244	KC977262	Kuhnert et al. (2014a); Stadler et al. (2014); Wendt et al. (2018)
*D.eschscholtzii*	MUCL 45435	KY610437	PQ632365	KY624246	KC977266	Wendt et al. (2018)
*D.petriniae*	MUCL 49214	KY610439	JX658512	KY624248	KC977261	Kuhnert et al. (2014a); Stadler et al. (2014); Wendt et al. (2018)
*D.placentiformis*	MUCL 47603	KY610440	AM749921	KY624249	KC977278	Wendt et al. (2018)
*D.vernicosa*	CBS 119316^T^	KY610442	NR_152501	KY624252	N/A	Wendt et al. (2018)
*Diatrypebetulaceicola*	FCATAS 2725^T^	–	OM040386	–	OM240966	Yang et al. (2022a)
*D.betulae*	CFCC 52416^T^	–	NR_177156	–	MW656391	Zhu et al. (2021)
*D.betulae*	GMB0426	–	OP935181	–	OP938750	Li et al. (2023)
*D.bullata*	UCDDCh400	–	DQ006946	–	DQ007002	Rolshausen et al. (2006)
*D.camelliae-japonicae*	GMB0427 ^T^	–	NR_198356	–	OP938734	Li et al. (2023)
*D.camelliae-japonicae*	GMB0428	–	OP935173	–	OP938735	Li et al. (2023)
*D.castaneicola*	CFCC 52425^T^	–	NR_177155	–	MW656389	Zhu et al. (2021)
*D.disciformis*	MFLU 17-1549	–	MW240629	–	N/A	Samarakoon et al. (2022)
*D.disciformis*	MFLUCC 15-0538^T^	–	KR092795	–	N/A	Senanayake et al. (2015)
*D.enteroxantha*	GMB0433	–	OP935170	–	OP938736	Li et al. (2023)
*D.enteroxantha*	HUEFS 155112	–	KM396624	–	KR869728	de Almeida et al. (2016)
*D.enteroxantha*	HUEFS 155116	–	KM396618	–	N/A	de Almeida et al. (2016)
*D.lancangensis*	GMB 0045^T^	–	NR_174916	–	MW814885	Long et al. (2021)
*D.lancangensis*	GMB 0046	–	MW797114	–	MW814886	Long et al. (2021)
*D.larissae*	FCATAS_2723^T^	–	OM040384	–	OM240964	Yang et al. (2022a)
*D.lijiangensis*	MFLU 19-0717^T^	–	MK852582	–	MK852583	Thiyagaraja et al. (2019)
*D.linzhiensis*	FCATAS 4304^T^	–	OP538691	–	OP557597	Ma et al. (2023)
*D.linzhiensis*	FCATAS 4381	–	OP538692	–	OP557598	Ma et al. (2023)
*D.palmicola*	MFLUCC 11-0018^T^	–	NR_185365	–	N/A	Liu et al. (2015)
*D.palmicola*	MFLUCC 11-0020	–	KP744439	–	N/A	Liu et al. (2015)
*D.quercicola*	CFCC 52418^T^	–	NR_177154	–	MW656386	Zhu et al. (2021)
*D.rubi*	GMB0429^T^	–	OP935182	–	OP938740	Li et al. (2023)
*D.rubi*	GMB0430	–	OP935183	–	OP938741	Li et al. (2023)
*D.spilomea*	D17C	–	AJ302433	–	N/A	Acero et al. (2004)
*D.stigma*	DCASH200	–	GQ293947	–	GQ294003	Trouillas et al. (2015)
*D.undulata*	D20C	–	AJ302436	–	N/A	Acero et al. (2004)
*D.virescens*	CBS:128344	–	MH864890	–	N/A	Vu et al. (2019)
*D.whitmanensis*	Bent023	–	OP038009	–	OP079844	Travadon et al. (2022)
*Diatrypellaatlantica*	HUEFS 136873	–	KM396614	–	KR259647	de Almeida et al. (2016)
*D.banksiae*	CPC 29118^T^	–	NR_154026	–	N/A	Crous et al. (2016)
*D.betulae*	CFCC 52406^T^	–	NR_177150	–	MW656379	Zhu et al. (2021)
*D.betulicola*	CFCC 52411^T^	–	NR_177152	–	MW656383	Zhu et al. (2021)
*D.delonicis*	MFLUCC 15-1014^T^	–	MH812994	–	MH847790	Hyde et al. (2019)
*D.delonicis*	MFLU 16-1032	–	MH812995	–	MH847791	Hyde et al. (2019)
*D.elaeidis*	MFLUCC 15-0279^T^	–	MN308417	–	MN340300	Konta et al. (2020a)
*D.fatsiae-japonica*	GMB 0422^T^	–	OP935184	–	OP938744	Li et al. (2023)
*D.fatsiae-japonica*	GMB 0423	–	OP935185	–	OP938745	Li et al. (2023)
*D.favacea*	DL26C	–	AJ302440	–	N/A	Acero et al. (2004)
*D.favacea*	CFCC 52409	–	MW632934	–	MW656382	Zhu et al. (2021)
*D.frostii*	UFMGCB 1917	–	HQ377280	–	N/A	Vieira et al. (2011)
*D.guiyangensis*	GMB 0414^T^	–	OP935188	–	OP938742	Li et al. (2023)
*D.guiyangensis*	GMB 0415	–	OP935189	–	OP938743	Li et al. (2023)
*D.heveae*	MFLUCC 15-0274	–	MN308418	–	MN340301	Konta et al. (2020b)
*D.heveae*	MFLUCC 17-0368^T^	–	MF959501	–	MG334557	Senwanna et al. (2017)
*D.hubeiensis*	CFCC 52413^T^	–	NR_177153	–	MW656385	Zhu et al. (2021)
*D.iranensis*	KDQ18 ^T^	–	KM245033	–	N/A	Mehrabi et al. (2015)
*D.kunmingensis*	KUNCC 23-15532^T^	–	PP584732	–	PP982304	Dissanayake et al. (2024)
*D.kunmingensis*	KUNCC 23-15533	–	PP584733	–	PP982305	Dissanayake et al. (2024)
*D.łongiasca*	KUMCC 20-0021^T^	–	MW039349	–	MW239658	Dissanayake et al. (2021)
*D.macrospora*	IRAN 2344C	–	KR605648	–	KY352430	Mehrabi et al. (2019)
*D.oregonensis*	CA117	–	GQ293934	–	GQ293996	Trouillas et al. (2010)
*D.oregonensis*	DPL200	–	GQ293940	–	GQ293999	Trouillas et al. (2010)
*D.pseudooregonensis*	GMB0041^T^	–	NR_174917	–	MW814890	Long et al. (2021)
*D.pseudooregonensis*	GMB0040	–	MW797117	–	MW814889	Long et al. (2021)
*D.pulvinata*	H048	–	FR715523	–	FR715495	de Almeida et al. (2016)
*D.pulvinata*	MEND-F-0815	–	OQ357998	–	OQ379485	Spetik et al. (2024)
*D.quercina*	MFLU 18-1865	–	ON705330	–	ON713468	Jayawardena et al. (2022)
*D.tectonae*	MFLUCC 12-0172a^T^	–	KY283084	–	N/A	Shang et al. (2017)
*D.tectonae*	MFLUCC 12-0172b^T^	–	KY283085	–	KY421043	Shang et al. (2017)
***D.thailandica***	**MFLU 24-0533^T^**	–	**PQ164184**	–	**PV443842**	This study
***D.thailandica***	**MFLU 24-0534**	–	**PV445684**	–	**PV443843**	This study
*D.verruciformis*	UCROK1467	–	JX144793	–	JX174093	Lynch et al. (2013)
*D.verruciformis*	UCROK754	–	JX144783	–	JX174083	Lynch et al. (2013)
*D.vulgaris*	HVFRA02	–	HQ692591	–	HO692503	Trouillas et al. (2011)
*D.vulgaris*	HVGRF03^T^	–	HQ692590	–	HQ692502	Trouillas et al. (2011)
*D.vulgaris*	KUNCC 23-15534	–	PP584734	–	PP982306	Dissanayake et al. (2024)
*D.vulgaris*	KUNCC 23-15535	–	PP584735	–	PP982307	Dissanayake et al. (2024)
*D.vulgaris*	KUNCC 23-15536	–	PP584736	–	PP982308	Dissanayake et al. (2024)
*D.vulgaris*	KUNCC 23-15537	–	PP584737	–	PP982309	Dissanayake et al. (2024)
*D.yunnanensis*	VT01^T^	–	MN653008	–	MN887112	Hyde et al. (2020a)
*Durothecacrateriformis*	GMBC0205^T^	MH645425	NG_068849	MH645427	MH645424	De Long et al. (2019)
*D.eurima*	CGMB0060	MH645417	MH645419	MH645418	MH049437	De Long et al. (2019)
*D.guizhouensis*	HKAS 101453^T^	NG_068848	NR_169679	MH645431	MH645428	De Long et al. (2019)
*D.rogersii*	GMBC0204	MH645434	MH645433	MH645435	MH645432	De Long et al. (2019)
*D.rogersii*	YMJ 92031201^T^	N/A	NR_186907	JX507794	EF025612	Hsieh et al. (2010)
*Entonaemaliquescens*	CNF 2/11263	OQ865124	OQ869784	OQ877106	OQ877117	Pošta et al. (2023)
*Eutypalaevata*	CBS 291.87	–	HM164737	–	HM164771	Trouillas and Gubler (2010)
*E.lata*	EP18	–	HQ692611	–	HQ692501	Trouillas et al. (2011)
*E.lata*	RGA01	–	HQ692614	–	HQ692497	Trouillas et al. (2011)
*E.armeniacae*	ATCC 28120	–	DQ006948	–	DQ006975	Rolshausen et al. (2006)
*E.camelliae*	HKAS 107022^T^	–	NR_175674	–	MW775593	Samarakoon et al. (2022)
*E.cerasi*	GMB 0048^T^	–	NR_174915	–	MW814893	Long et al. (2021)
*E.cerasi*	GMB 0049	–	MW797105	–	MW814877	Long et al. (2021)
*Eutypellacearensis*	HUEFS 131070	–	KM396639	–	N/A	de Almeida et al. (2016)
*E.cerviculata*	EL59C	–	AJ302468	–	N/A	Acero et al. (2004)
*E.cerviculata*	M68^T^	–	JF340269	–	N/A	Arhipova et al. (2012)
*E.parasitica*	CBS 210.39^T^	–	MH855984	–	N/A	Vu et al. (2019)
*E.quercina*	IRAN 2543C^T^	–	NR_171806	–	KY352449	Mehrabi et al. (2019)
*E.semicircularis*	MP4669	–	JQ517314	–	N/A	Mehrabi et al. (2016)
*E.motuoensis*	FCATAS 4379	–	OP538694	–	OP557600	Ma et al. (2023)
*E.motuoensis*	FCATAS 4082^T^	–	OP538693	–	OP557599	Ma et al. (2023)
*Graphostromaplatystomum*	CBS 270.87^T^	–	JX658535	KY624296	N/A	Wendt et al. (2018)
*Halocryptovalsasalicorniae*	MFLUCC 15-0185	–	MH304410	–	MH370274	Dayarathne et al. (2020a)
*Halodiatrypeavicenniae*	MFLUCC 15-0953^T^	–	KX573916	–	KX573931	Dayarathne et al.(2016)
*H.salinicola*	MFLUCC 15-1277^T^	–	KX573915	–	KX573932	Dayarathne et al.(2016)
*Haloroselliniakrabiensis*	MFLU 17-2596^T^	–	NR_166289	N/A	MN431493	Dayarathne et al. (2020b)
*H.oceanica*	BCC: 60405	–	MK606079	N/A	N/A	Yoiprommarat et al. (2019)
*H.rhizophorae*	MFLU 17-2591	–	MN047118	N/A	MN431492	Dayarathne et al. (2020b)
*H.xylocarpi*	MFLU 18-0545^T^	–	NR_166290	N/A	MN077076	Dayarathne et al. (2020b)
***H.xylocarpi***	**MFLU 24-0536**	–	**PV436687**	**N/A**	**N/A**	This study
***H.xylocarpi***	**MFLUCC 25-0025**	–	**PV436688**	**N/A**	**N/A**	This study
*Hypomontagnellabarbarensis*	STMA 14081^T^	MK131718	MK131720	MK135891	MK135893	Lambert et al. (2019)
***H.hibisci***	**MFLUCC 24-0613^T^**	**PV468230**	**PQ164174**	**PV476726**	**PV469694**	This study
***H.hibisci***	**MFLU 24-0532**	**N/A**	**PV459061**	**PV476727**	**PV469695**	This study
*H.monticulosa*	MUCL 54604^T^	KY610487	KY610404	KY624305	KX271273	Wendt et al. (2018)
***H.monticulosa***	**MFLUCC 24-0612**	**PV468229**	**PP980679**	**PQ999133**	**PQ999134**	This study
*H.submonticulosa*	CBS 115280	KY610457	KC968923	KY624226	KC977267	Kuhnert et al. (2014a); Wendt et al. (2018)
*Hypoxylonaddis*	MUCL 52797^T^	N/A	KC968931	N/A	KC977287	Kuhnert et al. (2014a)
*H.anthochroum*	YMJ 9	N/A	JN660819	N/A	AY951703	Hsieh et al. (2005)
*H.aurantium*	MFLU 16-1202^T^	NG_068298	NR_166287	MN077081	N/A	Dayarathne et al. (2020b)
*H.aurantium*	MFLU 18-0531	MN017879	MN047115	MN077081	N/A	Dayarathne et al. (2020b)
*H.aveirense*	MUM 19.40^T^	NG_243120	NR_173851	N/A	N/A	Cedeño-Sanchez et al. (2023)
*H.baruense*	UCH 9545^T^	NG_228989	NR_169973	PP732079	MK908142	Cedeño-Sanchez et al. (2023, 2024)
*H.begae*	YMJ 215	N/A	JN660820	N/A	AY951704	Hsieh et al. (2005)
*H.begae*	YMJ 215	N/A	JN660820	N/A	AY951704	Hsieh et al. (2005)
*H.bellicolor*	UCH 9543	N/A	MN056425	N/A	MK908139	Cedeño-Sanchez et al. (2020)
*H.bimaculatum*	GYJF21259	N/A	OR415331	N/A	N/A	Fournier et al. (2024)
*H.blackburniae*	BRIP 72467b^T^	NG_149119	NR_182618	N/A	N/A	Tan and Shivas (2022b)
*H.brevisporum*	YMJ 36	N/A	JN660821	N/A	AY951705	Hsieh et al. (2005)
*H.calileguense*	STMA 14059^T^	N/A	NR_167964	N/A	KU604579	Sir et al. (2016)
*H.calileguense*	STMA 14070	N/A	KU604565	KY624271	KU604578	Sir et al. (2016)
*H.canariense*	MUCL 47224	ON954140	ON792787	OP251029	N/A	Stadler et al. (2008)
*H.carneum*	MUCL 54177	KY610480	KY610400	KY624297	KX271270	Wendt et al. (2018)
*H.cercidicola*	CBS 119009	KY610444	KC968908	KY624254	KX271270	Kuhnert et al. (2014a); Wendt et al. (2018)
*H.chionostomum*	STMA 14060	ON954144	KU604563	OP251030	N/A	Cedeño-Sanchez et al. (2023)
*H.chrysalidosporum*	FCATAS2710^T^	OL615106	OL467294	OL584222	OL584229	Ma et al. (2022a)
*H.cinnabarinum*	UCH9546	N/A	MN056429	N/A	MK908143	Cedeño–Sanchez et al. (2020)
*H.crocopeplum*	CNF 2/11316^T^	OQ869786	OQ865120	OQ877107	OQ877118	Pošta et al. (2023)
*H.cyclobalanopsidis*	FCATAS2714^T^	OL615108	OL467298	OL584225	OL584232	Ma et al. (2022a)
*H.damuense*	FCATAS 4207^T^	ON075433	ON075427	ON093251	ON093245	Song et al. (2022)
*H.dieckmannii*	YMJ 45	N/A	JN979412	N/A	AY951712	Hsieh et al. (2005)
*H.dieckmannii*	YMJ 89041203	N/A	JN979413	N/A	AY951713	Hsieh et al. (2005)
*H.erythrostroma*	MUCL 53759	ON954154	KC968910	OP251031	KC977296	Kuhnert et al. (2014a)
*H.eurasiaticum*	MUCL 57720^T^	N/A	MW367851	MW373852	MW373861	Lambert et al. (2021)
*H.fendleri*	MUCL 54792	KY610481	KF234421	KY624298	KF300547	Kuhnert et al. (2014a); Wendt et al. (2018)
*H.fendleri*	DSM:107927	MK287545	MK287533	MK287558	MK287571	Sir et al. (2019)
*H.ferrugineum*	CBS 141259	N/A	KX090079	N/A	KX090080	Friebes and Wendelin (2016)
*H.flavoargillaceum*	STMA 14062	N/A	KU604577	N/A	KU159532	Sir et al. (2016)
*H.fragiforme*	YMJ 383	N/A	JN979420	N/A	AY951720	Hsieh et al. (2005)
*H.fragiforme*	MUCL 51264^T^	KM186295	KC477229	KM186296	KX271282	Stadler et al. (2013); Daranagama et al. (2015); Wendt et al. (2018)
*H.fraxinophilum*	MUCL 54176^T^	N/A	KC968938	N/A	KC977301	Kuhnert et al. (2014a)
*H.fuscoides*	MUCL 52670	ON954145	ON792789	OP251038	ON813076	Cedeño-Sanchez et al. (2023)
*H.fuscopurpureum*	YMJ 67	N/A	JN979421	N/A	AY951721	Hsieh et al. (2005)
*H.fuscum*	CBS 113049^T^	KY610482	KY610401	KY624299	KX271271	Wendt et al. (2018)
*H.fuscum*	STMA 13090	KY610482	KY610401	N/A	KX271271	Cedeño-Sanchez et al. (2023)
*H.fuscum*	43621	MW367847	MW367856	MW373857	MW373866	Lambert et al. (2021)
*H.gibriacense*	MUCL 52698^T^	NG_228990	NR_137100	OP251026	ON813074	Fournier et al. (2010a)
*H.griseobrunneum*	CBS 331.73^T^	MH872399	KY610402	KY624300	KC977303	Kuhnert et al. (2014a); Wendt et al. (2018); Vu et al. (2019)
*H.guilanense*	MUCL 57726^T^	MT214992	MT214997	MT212235	MT212239	Pourmoghaddam et al. (2020)
*H.guizhouense*	KUNCC 23-15544^T^	NG_244017	PP584753	PP993509	PP951419	Dissanayake et al. (2024)
*H.guizhouense*	KUNCC 23-15545	PP584827	PP584754	PP993510	PP951420	Dissanayake et al. (2024)
*H.haematostroma*	MUCL 53301^T^	KY610484	KC968911	KY624301	KC977291	Lambert et al. (2019)
*H.hainanense*	FCATAS2712^T^	OL616132	NR_184951	OL584224	OL584231	Ma et al. (2022a)
*H.hinnuleum*	CBS 286.62^T^	NG_064032	NR_145212	N/A	N/A	Bitzer et al. (2008)
*H.hinnuleum*	DSM 107932	MK287547	N/A	MK287560	MK287573	Sir et al. (2019)
*H.hongheensis*	KUMCC 21-0452	OM001334	OM001333	ON392008	ON468655	Yang et al. (2022b)
*H.hongheensis*	HKAS 122663	OM001339	OM001336	ON392009	ON468656	Yang et al. (2022b)
*H.howeanum*	MUCL 47599	KY610448	AM749928	KY624258	KC977277	Bitzer et al. (2008); Kuhnert et al. (2014a); Wendt et al. (2018)
*H.howeanum*	CNF 2/11315	OQ865215	OQ865216	OQ877109	OQ877120	Pošta et al. (2023)
*H.hypomiltum*	MUCL 51845	KY610449	KY610403	KY624302	KX271249	Wendt et al. (2018)
*H.inaequale*	HKAS 123207^T^	N/A	NR_185719	N/A	OQ652093	Jayawardena et al. (2022)
*H.invadens*	MUCL 51475^T^	MT809132	MT809133	MT813037	MT813038	Becker et al. (2020)
*H.investiens*	CBS 118183	KY610450	KC968925	KY624259	KC977270	Kuhnert et al. (2014a); Wendt et al. (2018)
*H.isabellinum*	MUCL 53308^T^	NG_228991	NR_155157	OP251032	KC977295	Cedeño-Sanchez et al. (2023)
*H.jaklitschii*	CBS 135869	N/A	KM610281	N/A	KM610295	Kuhnert et al. (2015)
*H.jecorinum*	YMJ 39	N/A	JN979429	N/A	AY951731	Hsieh et al. (2005)
*H.jianfengense*	FCATAS845^T^	MZ029707	MW984546	MZ047260	MZ047264	Song et al. (2022)
*H.laschii*	MUCL 52796	ON954147	JX658525	OP251027	ON813075	Stadler et al. (2014)
*H.lateripigmentum*	MUCL 53304^T^	KY610486	KC968933	KY624304	KC977290	Kuhnert et al. (2014a); Wendt et al. (2018)
*H.lechatii*	MUCL 54609	ON954148	KF923407	OP251033	KF923405	Kuhnert et al. (2014b)
*H.lenormandii*	CBS 119003	KY610452	KC968943	KY624261	KC977273	Wendt et al. (2018)
*H.lenormandii*	MFLUCC 13-0311	KM039136	KM039135	KM039137	KM039138	Daranagama et al. (2015)
*H.lienhwacheense*	MFLUCC 14-1231	MK287550	KU604558	MK287563	KU159522	Sir et al. (2019)
*H.liviae*	CBS 115282^T^	N/A	NR_155154	N/A	KC977265	Kuhnert et al. (2014a)
*H.lividicolor*	YMJ 70	N/A	JN979432	N/A	AY951734	Hsieh et al. (2005)
*H.lividipigmentum*	STMA14045	ON954149	ON792788	PP732080	ON813077	Cedeño-Sanchez et al. (2023)
*H.lividipigmentum*	YMJ 233	N/A	JN979433	N/A	AY951735	Hsieh et al. (2005)
*H.macrocarpum*	CBS 119012	ON954151	ON792785	ON813071	OP251034	Cedeño-Sanchez et al. (2023)
*H.mangrovei*	MFLU 18-0559^T^	NG_068299	NR_166288	N/A	MN077053	Dayarathne et al. (2020b)
*H.mangrovei*	MFLU 18-0575	MN017881	MN047117	N/A	MN077054	Dayarathne et al. (2020b)
*H.medogense*	FCATAS 4061^T^	ON075431	ON075425	ON093249	ON093243	Song et al. (2022)
*H.medogense*	FCATAS4320	ON075432	ON075426	ON093250	ON093244	Song et al. (2022)
*H.munkii*	MUCL 53315	ON954153	KC968912	OP251035	KC977294	Kuhnert et al. (2014a); Cedeño-Sanchez et al. (2023)
*H.munkii*	YMJ 90080403	N/A	JN979436	N/A	AY951738	Hsieh et al. (2005)
*H.musceum*	MUCL 53765	KY610488	KC968926	KY624306	KC977280	Kuhnert et al. (2014a); Wendt et al. (2018)
*H.notatum*	YMJ 250	N/A	JQ009305	N/A	AY951739	Hsieh et al. (2005)
*H.ochraceum*	MUCL 54625^T^	N/A	NR_155158	KY624271	KC977300	Kuhnert et al. (2014a)
*H.olivaceopigmentum*	DSM: 107924^T^	MK287542	MK287530	MK287555	MK287568	Sir et al. (2019)
*H.perforatum*	CBS115281	KY610455	KY610391	KY624224	KX271250	Wendt et al. (2018)
*H.petriniae*	CBS 114746^T^	KY610491	NR_155185	KY624279	KX271274	Wendt et al. (2018)
*H.pilgerianum*	YMJ 92042505	N/A	JQ009310	N/A	AY951744	Hsieh et al. (2005)
*H.polyporoideum*	YMJ 15	N/A	JQ009311	N/A	AY951747	Hsieh et al. (2005)
*H.polyporoideum*	YMJ 56	N/A	JQ009312	N/A	AY951748	Hsieh et al. (2005)
*H.porphyreum*	CBS 119022	KY610456	KC968921	KY624225	KC977264	Kuhnert et al. (2014a); Wendt et al. (2018)
*H.pseudofuscum*	DSM 112038^T^	MW367849	NR_172359	MW373859	MW373868	Lambert et al. (2021)
*H.pulicicidum*	CBS 122622^T^	NG_066188	JX183076	KY624280	JX183074	Wendt et al. (2018)
*H.rickii*	MUCL 53309^T^	KY610416	KC968932	KY624281	KC977288	Wendt et al. (2018)
*H.rickii*	YMJ 25	N/A	JQ009313	N/A	AY951750	Hsieh et al. (2005)
*H.rubiginosum*	MUCL 52887^T^	KY610469	KC477232	KY624266	KY624311	Stadler et al. (2013); Wendt et al. (2018)
*H.rubiginosum*	423	MT214993	MT214998	MT212236	MT212240	Pourmoghaddam et al. (2020)
*H.rutilum*	YMJ 181	N/A	N/A	N/A	AY951752	Hsieh et al. (2005)
*H.samuelsii*	MUCL 51843^T^	KY610466	KC968916	KY624269	KC977286	Kuhnert et al. (2014a); Wendt et al. (2018)
*H.spegazzianum*	STMA 14082^T^	N/A	KU604573	N/A	KU604582	Sir et al. (2016)
*H.sporistriatatunicum*	UCH9542^T^	N/A	MN056426	N/A	MK908140	Cedeño-Sanchez et al. (2020)
*H.subgilvum*	STMA 24034	PP729637	PP718985	PP732078	PP721317	Cedeño-Sanchez et al. (2024)
*H.sublenormandii*	JF13026^T^	N/A	KM610291	N/A	KM610303	Kuhnert et al. (2015)
*H.subrutiloides*	F202416	N/A	FJ185304	N/A	FJ185281	Platas et al. (2009)
*H.cf.subticinense*	MUCL 53752	ON954152	KC968913	N/A	KC977297	Kuhnert et al. (2014a)
*H.szostakii*	BRIP 72527b^T^	NG_149115	NR_182620	N/A	N/A	Tan and Shivas (2022a)
*H.texense*	DSM 107933^T^	MK287548	MK287536	MK287561	MK287574	Sir et al. (2019)
*H.texense*	DSM 107928	MK287538	MK287527	MK287551	MK287564	Sir et al. (2019)
***H.thailandicum***	**MFLUCC 25-0024^T^**	**PV468231**	**PQ164172**	**PV476728**	**PV469696**	This study
***H.thailandicum***	**MFLU 24-0530**	**PV468232**	**PV469663**	**N/A**	**PV469697**	This study
*H.ticinense*	CBS 115271	KY610471	JQ009317	KY624272	AY951757	Hsieh et al. (2005); Wendt et al. (2018)
*H.ticinense*	CNF 2/11314	OQ865219	OQ869783	OQ877110	OQ877121	Pošta et al. (2023)
*H.trugodes*	MUCL 54794^T^	NG_066380	KF234422	KY624282	KF300548	Kuhnert et al. (2014a); Wendt et al. (2018)
*H.ulmophilum*	YMJ 350	N/A	JQ009320	N/A	AY951760	Hsieh et al. (2005)
*H.vogesiacum*	CBS 115273	KY610417	KC968920	KY624283	KX271275	Wendt et al. (2018)
*H.wujianggense*	GMBC0213^T^	MT568853	MT568854	MT585802	MT572481	Pi et al. (2020)
*H.wuzhishanense*	FCATAS2708^T^	OL615104	OL467292	OL584220	OL584227	Ma et al. (2022a)
*H.zangii*	FCATAS 4029^T^	ON075429	ON075423	ON093247	ON093241	Song et al. (2022)
*H.zangii*	FCATAS 4319	ON075424	ON075424	ON093248	ON093242	Song et al. (2022)
*H.zhaotongensis*	GMBCC1168^T^	OP598100	OP597690	OP615662	OP615660	Zhang et al. (2023a)
*H.zhaotongensis*	GMBCC1157	OP598101	OP597691	OP615663	OP615661	Zhang et al. (2023a)
*Jackrogersellacohaerens*	CBS 119126	KY610396	KY610497	KY624270	KY624314	Wendt et al. (2018)
*J.multiformis*	CBS 119016^T^	KY610473	KC477234	KY624290	KX271262	Kuhnert et al. (2014b); Wendt et al. (2018)
*Kretzschmariadeusta*	CBS 826.72	–	KU683767	–	KU684190	U’Ren et al. (2016)
*Kretzschmariellaculmorum*	JDR 88	–	KX430043	KX430045	KX430046	Hsieh et al. (2005)
*Monosporascuscannonballus*	ZJUP0990-2	–	OR357656	–	OR365762	Jin et al. (2024)
*M.cannonballus*	ATCC 26931^T^	–	NR_111370	–	N/A	Schoch et al. (2014)
*Natonodosaspeciosa*	CLM-RV86	N/A	MF380435	MH745150	N/A	Heredia et al. (2020)
*Neoeutypellabaoshanensis*	HKAS 133108	–	PP584738	–	PP982310	Dissanayake et al. (2024)
*N.baoshanensis*	HKAS 133107	–	PP584739	–	PP982311	Dissanayake et al. (2024)
*Paraeutypellacitricola*	HKAS 133112	–	PP584747	–	PP951424	Dissanayake et al. (2024)
*P.citricola*	HKAS 133113	–	PP584749	–	PP951426	Dissanayake et al. (2024)
*P.citricola*	HKAS 133114	–	PP584748	–	PP951425	Dissanayake et al. (2024)
***P.citricola***	**MFLUCC 24-0614**	–	**PV426438**	–	**PV387964**	This study
*P.karsti*	GZAAS 20-4001^T^	–	OR225064	–	OR189507	Zhang et al. (2023b)
*P.longiasca*	GZAAS 19-1765^T^	–	OR225065	–	OR189505	Zhang et al. (2023b)
*P.subguizhouensis*	GMB0420^T^	–	NR_198359	–	OP938748	Li et al. (2023)
*P.subguizhouensis*	GMB0421	–	OP935187	–	OP938749	Li et al. (2023)
*P.vitis*	UCD2291AR	–	HQ288224	–	HQ288303	Urbez-Torres et al. (2012)
*P.vitis*	UCD2428TX	–	FJ790851	–	GU294726	Urbez-Torres et al. (2012)
*Parahypoxylonpapillatum*	ATCC 58729^T^	KY610454	NR_155153	KY624223	N/A	Kuhnert et al. (2014a)
*P.ruwenzoriense*	MUCL51392^T^	NG_243121	NR_191199	OP251039	ON813078	Cedeño-Sanchez et al. (2023)
*Pedumisporarhizophorae*	BCC44877	–	KJ888853	–	N/A	Klaysuban et al. (2014)
*P.rhizophorae*	BCC44878	–	KJ888854	–	N/A	Klaysuban et al. (2014)
*Peroneutypaalsophila*	EL58C	–	AJ302467	–	N/A	Acero et al. (2004)
*P.curvispora*	HUEFS 136877	–	KM396641	–	N/A	de Almeida et al. (2016)
*P.diminutiasca*	MFLUCC 17-2144^T^	–	MG873479	–	MH316765	Shang et al. (2018)
*P.kunmingensis*	HKAS 113189^T^	–	MZ475070	–	MZ490589	Hyde et al. (2020a)
*P.longiasca*	MFLU 17-1217^T^	–	NR_154386	–	MG334558	Senwanna et al. (2017)
*P.mackenziei*	MFLUCC 16-0072^T^	–	NR_154363	–	KY706363	Shang et al. (2017)
*P.rubiformis*	MFLU 17-1185^T^	–	NR_158867	–	MH316763	Shang et al. (2018)
*Pseudodiatrypehainanensis*	GMB 0054^T^	–	NR_174076	–	MW814883	Long et al. (2021)
*P.hainanensis*	GMB 0055	–	MW797112	–	MW814884	Long et al. (2021)
*Pyrenopolyporushunteri*	MUCL 52673	KY610472	KY610421	N/A	N/A	Wendt et al. (2018)
*P.laminosus*	TBRC:8871	MH938536	MH938527	MK165424	MK165415	Wongkanoun et al. (2019)
*P.nicaraguensis*	CBS 117739	KY610489	AM749922	KY624307	KC977272	Kuhnert et al. (2014a); Wendt et al. (2018)
*Quaternariaquaternata*	MFLU:15-2605	–	MT185553	–	N/A	Li et al. (2020)
*Q.quaternata*	GNF13	–	KR605645	–	KY352464	Mehrabi et al. (2016)
*Rhopalostromaangolense*	CBS 126414	MH875559	MH864100	KY624228	KX271277	Wendt et al. (2018); Vu et al. (2019)
*Rostrohypoxylonterebratum*	CBS 119137^T^	NG_057759	NR_137677	DQ631954	DQ840097	Fournier et al. (2010b)
*Ruwenzoriapseudoannulata*	MUCL 51394	KY610494	NR_137733	KY624286	KX271278	Stadler et al. (2010a)
***Stilbohypoxylonchiangraiense***	**MFLUCC 24-0611^T^**	–	**PQ165948**	**PV469698**	**PV476729**	This study
***S.chiangraiense***	**MFLU 24-0529**	–	**PV469662**	**N/A**	**PV476730**	This study
*S.elaeidicola*	GMB0763	–	PP153399	N/A	PP209120	Li et al. (2024)
*S.elaeicola*	JDR 173	–	EF026148	GQ844826	EF025616	Hsieh et al. (2010)
*S.elaeicola*	HAST 94082615	–	GU322440	GQ844827	GQ495933	Hsieh et al. (2010)
*S.elaeidis*	MFLUCC 15-0295a	–	MT496745	MT502416	MT502420	Konta et al. (2020a)
*S.elaeidis*	MFLUCC 15-0295b	–	MT496746	MT502417	MT502421	Konta et al. (2020a)
*S.quisquiliarum*	JDR 172	–	EF026119	GQ853020	EF025605	Hsieh et al. (2010)
*Thamnomycesdendroidea*	CBS 123578^T^	KY610467	NR_154472	KY624232	KY624313	Stadler et al. (2010b)
*Vasilyevacinnamomi*	GMB0418^T^	–	OP935174	–	OP938737	Li et al. (2023)
*V.cinnamomi*	GMB0419	–	OP935175	–	OP938738	Li et al. (2023)
*Xylariaacuminatilongissima*	HAST 623^T^	–	NR_147516	GQ853028	GQ502711	Hsieh et al. (2010)
*X.adscendens*	HAST 570	–	GU300101	GQ844817	GQ487708	Hsieh et al. (2010)
*X.aethiopica*	YMJ 1136^T^	–	MH790445	MH785222	MH785221	Fournier et al. (2018)
*X.allantoidea*	HAST 94042903	–	GU324743	GQ848356	GQ502692	Hsieh et al. (2010)
*X.apoda*	HAST 90080804	–	GU322437	GQ844823	GQ495930	Hsieh et al. (2010)
*X.arbuscula*	CBS 126415	MH875560	MH864101	KY624287	KX271257	Vu et al. (2019)
*X.arbuscula*	HAST 89041211	–	GU300090	GQ844805	GQ478226	Hsieh et al. (2010)
X.arbusculavar.plenofissura	HAST 93082814	–	GU339495	GQ844804	GQ478225	Hsieh et al. (2010)
*X.atrodivaricata*	HAST 95052001^T^	–	EU178739	GQ853030	GQ502713	Hsieh et al. (2010)
*X.badia*	HAST 95070101	–	GU322446	GQ844833	GQ495939	Hsieh et al. (2010)
*X.bambusicola*	JDR 162	–	GU300088	GQ844801	GQ478223	Hsieh et al. (2010)
*X.berteri*	JDR 256	–	GU324750	GQ848363	GQ502698	Hsieh et al. (2010)
*X.brunneovinosa*	HAST 720^T^	–	NR_153201	GQ853023	GQ502706	Hsieh et al. (2010)
*X.castorea*	PDD 600	–	GU324751	GQ853018	GQ502703	Hsieh et al. (2010)
*X.cf.glebulosa*	HAST 431	–	GU322462	GQ848345	GQ495956	Hsieh et al. (2010)
*X.cirrata*	HAST 664^T^	–	EU179863	GQ853024	GQ502707	Hsieh et al. (2010)
*X.coccophora*	HAST 786	–	GU300093	GQ844809	GQ487701	Hsieh et al. (2010)
*X.crozonensis*	HAST 398	–	GU324748	GQ848361	GQ502697	Hsieh et al. (2010)
*X.cubensis*	HAST 477	–	N/A	GQ848364	GQ502699	Hsieh et al. (2010)
*X.culleniae*	JDR 189	–	GU322442	GQ844829	GQ495935	Hsieh et al. (2010)
*X.escharoidea*	HAST 658^T^	–	EU179864	GQ853026	GQ502709	Hsieh et al. (2010)
*X.fabacearum*	MFLUCC 16-0456 ^T^	–	NR_171104	MT212202	MT212220	Perera et al. (2020)
*X.fabaceicola*	MFLUCC 16-0461^T^	–	NR_171103	MT212201	MT212219	Perera et al. (2020)
*X.feejeensis*	HAST 92092013	–	GU322454	GQ848336	GQ495947	Hsieh et al. (2010)
*X.fimbriata*	HAST 491	–	GU324753	GQ853022	GQ502705	Hsieh et al. (2010)
*X.glebulosa*	GMB1053	–	PP153391	PP198097	PP209117	Li et al. (2024)
*X.grammica*	HAST 479	–	GU300097	GQ844813	GQ487704	Hsieh et al. (2010)
*X.griseosepiacea*	HAST 641^T^	–	EU179865	GQ853031	GQ502714	Hsieh et al. (2010)
*X.hypoxylon*	CBS 122620^T^	–	KY610407	KY624231	KX271279	Wendt et al. (2018)
*X.hypoxylon*	HAST 152	–	GU300096	GQ844812	GQ260187	Hsieh et al. (2010)
*X.ianthinovelutina*	HAST 553	–	GU322441	GQ844828	GQ495934	Hsieh et al. (2010)
*X.intraflava*	HAST 725^T^	–	EU179866	GQ853035	GQ502718	Hsieh et al. (2010)
*X.juruensis*	HAST 92042501	–	GU322439	GQ844825	GQ495932	Hsieh et al. (2010)
*X.laevis*	HAST 419	–	GU324746	GQ848359	GQ502695	Hsieh et al. (2010)
*X.laevis*	HAST 95072910	–	GU324747	GQ848360	GQ502696	Hsieh et al. (2010)
*X.liquidambaris*	HAST 93090701	–	GU300094	GQ844810	GQ487702	Hsieh et al. (2010)
*X.longissima*	GMB1076	–	PP146609	PP198095	PP209118	Li et al. (2024)
*X.multiplex*	JDR 259	–	GU300099	GQ844815	GQ487706	Hsieh et al. (2010)
*X.nigripes*	HAST 653	–	GU324755	GQ853027	GQ502710	Hsieh et al. (2010)
*X.oxyacanthae*	JDR 859	–	GU322434	GQ844820	GQ495927	Hsieh et al. (2010)
*X.oxyacanthae*	YMJ 1320	–	MF773431	MF773435	MF773439	Ju et al. (2018)
*X.palmicola*	PDD 604	–	GU322436	GQ844822	GQ495929	Hsieh et al. (2010)
*X.phyllocharis*	HAST 528	–	GU322445	GQ844832	GQ495938	Hsieh et al. (2010)
*X.plebeja*	HAST 91122401	–	GU324740	GQ848353	GQ502689	Hsieh et al. (2010)
*X.polymorpha*	JDR 1012	–	GU322460	GQ848343	GQ495954	Hsieh et al. (2010)
*X.reevesiae*	HAST 90071609	–	GU322435	GQ844821	GQ495928	Hsieh et al. (2010)
*X.regalis*	HAST 920	–	GU324745	GQ848358	GQ502694	Hsieh et al. (2010)
*X.rogersii*	FCATAS 915^T^	–	NR_184943	MZ707121	N/A	Ma et al. (2022b)
*X.schimicola*	FCATAS 896^T^	–	NR_184945	MZ707114	MZ695787	Ma et al. (2022b)
*X.schweinitzii*	HAST 92092023	–	GU322463	GQ848346	GQ495957	Hsieh et al. (2010)
*X.striata*	HAST 304	–	GU300089	GQ844803	GQ478224	Hsieh et al. (2010)
*X.theaceicola*	FCATAS 903^T^	–	NR_184944	MZ707115	MZ695788	Ma et al. (2022b)
*X.venosula*	HAST 94080508	–	EF026149	GQ844806	EF025617	Hsieh et al. (2010)
*X.venustula*	HAST 88113002	–	GU300091	GQ844807	GQ487699	Hsieh et al. (2010)
*X.vivantii*	HAST 519	–	GU322438	GQ844824	GQ495931	Hsieh et al. (2010)
*X.wallichii*	FCATAS 923	–	MZ648861	MZ707118	MZ695793	Ma et al. (2022b)
*X.xylarioides*	CBS 127883	–	KP218909	N/A	N/A	Vu et al. (2019)
*Xylotumulusgibbisporus*	ATCC MYA-4109^T^	–	NR_119711	N/A	N/A	Schoch et al. (2014)

N/A- Sequences not available; “–” - Sequences not used for analyses.

### ﻿Phylogenetic analyses

The quality of the sequence chromatograms was checked using BioEdit v. 7.0.9.0 (Hall 1999). Forward and reverse sequences were assembled into consensus sequences using Lasergene SeqMan Pro v. 7. Newly generated sequences were searched using the BLASTn search engine at NCBI (https://www.ncbi.nlm.nih.gov) against the GenBank database, and related literature was referred to (Thiyagaraja et al. 2019; Karimi et al. 2023). Each locus (LSU, ITS, β-tub, and rpb2) was individually aligned using MAFFT 6.864b (Katoh et al. 2019), trimmed using trimAl v. 1.2 software (Capella-Gutiérrez et al. 2009), and manually adjusted for improvement where necessary using BioEdit v. 7.2 (Hall 1999). Single gene alignments and the concatenated aligned dataset were analyzed separately using ML and BI. Best-fit models for BI analyses were selected using MrModeltest v. 2.2 (Nylander 2004) under the AIC (Akaike Information Criterion) implemented in PAUP v. 4.0b10. The GTR+G model was selected as the best model for BI analyses for all gene regions.

The ML analyses were performed using IQ-TREE with bootstrap support obtained from 1,000 pseudoreplicates (Nguyen et al. 2015; Chernomor et al. 2016). The BI analyses were conducted with MrBayes v. 3.2.6 (Ronquist et al. 2012). The Markov Chain Monte Carlo (MCMC) algorithm of six chains was initiated for 1,000,000 generations. The trees were sampled at every 100^th^ generation, resulting in 10,000 trees. The first 10% of trees were discarded as the burn-in phase, while the remaining 90% were used to calculate the posterior probabilities (PP) in the majority rule consensus tree. Phylograms were visualized in the FigTree v. 1.4.0 program (Rambaut 2012) and reorganized in Microsoft PowerPoint (2010).

### ﻿Preliminary screening for antibacterial activity

Preliminary screening for antibacterial activity was conducted for new isolates from the present study, along with selected existing Xylariales cultures from the Mae Fah Luang University Culture Collection (MFLUCC), following the methods described by Mapook et al. (2020). Ampicillin antibacterial discs were used as a positive control for the screening tests (Alam et al. 2019). The agar plug diffusion method was employed to assess antibacterial activity against *Bacillussubtilis* (gram-positive bacteria) and *Escherichiacoli* (gram-negative bacteria) (Balouiri et al. 2016). Both bacterial strains were cultured on nutrient agar (NA) for 24 hours. Prior to inoculating the Mueller-Hinton agar medium, bacterial cell concentrations were determined using a hemocytometer (6.7 × 10^5^ cells/mL), as described by Mapook et al. (2020). Fungal mycelial plugs from the test samples were transferred onto Mueller-Hinton agar plates and incubated at room temperature (25 °C) for 24 hours. Inhibition zones were measured and compared with both the positive and negative controls.

### ﻿Chemical extraction and HPLC/Q-TOF analyses

Xylariales cultures that showed positive results in the antibacterial activity assay were selected for fermentation and crude extraction. Culture conditions for the selected fungi were optimized using potato dextrose broth (PDB; potatoes, infusion from 200 g/L; dextrose, 20 g/L; pH 5.1 ± 0.2), yeast malt broth (YMB; dextrose, 10 g/L; malt extract, 3 g/L; peptic digest of animal tissue, 5 g/L; yeast extract, 3 g/L; pH 6.2 ± 0.2), and malt extract broth (MEB; malt extract, 30 g/L; mycological peptone, 5 g/L; agar, 15 g/L; pH 5.4 ± 0.2). Five mycelial plugs were cut from freshly grown cultures on each of the three media using a sterilized cork borer. These plugs were inoculated into 250 mL of the respective liquid medium in 500 mL Erlenmeyer flasks. The flasks were incubated on an Innova rotary shaker 43/43R (Eppendorf, Germany) at 140 rpm at 23 °C for 3–5 days. During fermentation, glucose depletion was monitored daily using Bayer Harnzuckerstreifen glucose test strips (Bayer, Leverkusen, Germany), and pH levels were measured using litmus paper (Merck KGaA, Darmstadt, Germany). When glucose levels reached zero and the pH dropped below 7.0, the cultures were processed for extraction.

Fungal mycelium and supernatant were separated by vacuum filtration. Crude extracts were prepared from both fractions. For the supernatant, ethyl acetate extraction was performed three times by mixing it with an equal volume of ethyl acetate in a separatory funnel. An equal volume of acetone was added to the mycelium, which was then freeze-dried and extracted three times with methanol (MeOH) at 40 °C in an ultrasonic bath for 30 minutes (Hellwig et al. 2005). After centrifugation at 1000 × *g* for 10 minutes, the supernatants were evaporated, and 50–100 mL of deionized water was added. Ethyl acetate extraction was then performed three times on the mycelium using an equal volume of ethyl acetate to yield a crude extract. These extracts were weighed.

Crude extracts obtained from both supernatant and mycelium were dissolved in methanol to a final concentration of 1 mg/mL. The solutions were filtered through a 0.22 µm membrane filter to remove particulates prior to HPLC injection. High-performance liquid chromatography (HPLC) was conducted using a Waters ACQUITY Arc System. Detection was performed with a photodiode array (PDA) detector and a fluorescence (FLR) detector (Waters, USA) using a reverse-phase C18 column (Kinetex® 5 µm EVO C18 100 Å, 150 × 2.1 mm LC) maintained at 30 °C. The mobile phase consisted of water with 0.2% formic acid (A) and methanol (B). Elution was carried out at a flow rate of 0.2 mL/min using the following linear gradient: 5–30% B (0–8 min), 30% B (8–10 min), 30–95% B (10–18 min), 95% B (18–22 min), 95–5% B (22–25 min), and 5% B (25–32 min). Samples were analyzed using a photodiode array detector set at 256 and 425 nm. Data analysis was conducted using Empower 3 software.

Liquid chromatography–quadrupole time-of-flight mass spectrometry (LC-QTOF-MS) was performed using an Agilent II/G6545B QTOF/MS and 1290 Infinity system equipped with an electrospray ionization (ESI) source. The instrument was operated in both positive and negative ionization modes under UHPLC pressure (1,300) and a mass range of 100–10,000.

## ﻿Results

### ﻿ Astrocystis

Taxon classificationFungiAscomycotaXylariales

Berk. & Broome, J. Linn. Soc., Bot. 14(no. 74): 123 (1873) [1875]

E52E5287-8EFF-559F-8D79-FC67E4A3E461

Index Fungorum: IF439

Facesoffungi Number: FoF00420

#### Notes.

Berkeley and Broome (1875) introduced *Astrocystis*, with *A.mirabilis* as the type species. Morphologically, this genus is characterized by uni- or occasionally multi-peritheciate stromata development, often beneath the host cuticle or on the surface; relatively short stipe asci; and ascus apical apparatus that are relatively small, amyloid, and stopper-shaped (Smith et al. 2003). Currently, 42 records are available in the Index Fungorum (2025).

##### ﻿Phylogenetic analyses for *Astrocystis*

**Figure 1.**
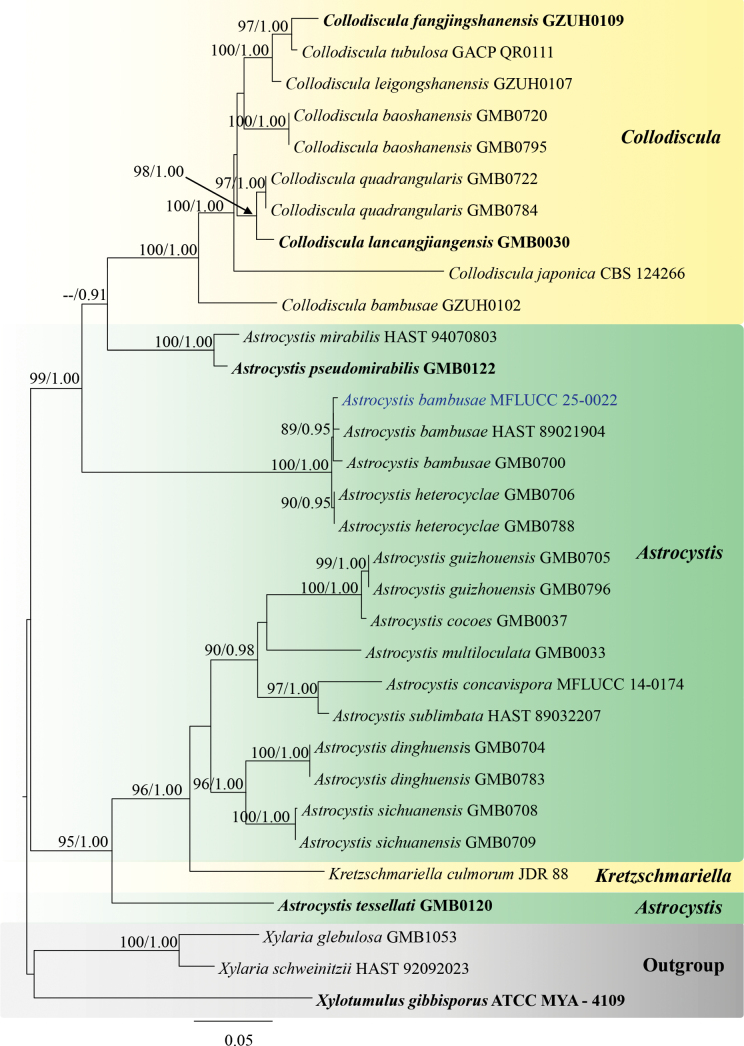
Phylogram generated from ML analysis based on the combined dataset of ITS, *β-tub*, and *rpb2*. The tree is rooted to *Xylotumulusgibbisporus* (ATCC MYA-4109), *Xylariaglebulosa* (GMB1053), and *X.schweinitzii* (HAST 92092023). Bootstrap support values for ML ≥ 70% and Bayesian posterior probabilities (PP) ≥ 0.90 are noted at the node. Strain numbers are noted after the species names. Strains isolated in this study are represented in blue, and type strains are in bold.

For *Astrocystis*, 32 taxa were included in the combined data set (ITS, *β-tub*, and *rpb2*) with *Xylotumulusgibbisporus* (ATCC MYA-4109), *Xylariaglebulosa* (GMB1053), and *X.schweinitzii* (HAST 92092023) as outgroup taxa. The final alignment consisted of 1953 characters, including gaps (ITS = 435 bp, *β-tub* = 514 bp, and *rpb*2 = 1004 bp). Both ML and BI analyses exhibit similar tree topology. The best-scoring RAxML tree was obtained (Fig. 1), with a final likelihood value of -12708.0675. The matrix included 851 distinct alignment patterns, with 17.88% undetermined characters or gaps. Estimated base frequencies were as follows: A = 0.242776, C = 0.267065, G = 0.262377, and T = 0.227782; substitution rates were AC = 1.393798, AG = 4.050932, AT = 1.300292, CG = 1.282338, CT = 7.225689, and GT = 1.0; and the gamma distribution shape parameter α = 0.297082. In the BI analyses, the average standard deviation of the split frequencies was 0.006 after 1,000,000 generations of runs. The phylogenetic tree topology is similar to the previous study by Li et al. (2024). According to the phylogenetic analyses, our strain MFLUCC 25-0022 clades within *Astrocystis*, with *Astrocystisbambusae* strains (HAST 8902190 and GMB0700).

###### ﻿Taxonomy

### ﻿ Astrocystis bambusae

Taxon classificationFungiAscomycotaXylariales

(Henn.) Læssøe & Spooner, Kew Bull. 49(1): 13 (1994) [1993]

5B8DF55F-6917-52A6-9668-B5754B8EF78E

Index Fungorum: IF361739

Facesoffungi Number: FoF17292

Fig. 2

Rosellinia bambusae Henn. 1908. Basionym.

#### Description.

***Saprobic*** on dead culms of *Bambusavulgaris*. ***Sexual morph*: *Stromata*** 1.2–0.9 mm diam., 0.8–1 mm high, scattered, solitary, superficial, black, appear as black raised spots on the host surface, hexagonal prism-shaped, containing one ascoma, with a circle of black tissue at the bottom. ***Perithecia*** 0.5–0.7 mm diam., 0.4–0.5 mm high, comprising black, fragile, carbonaceous tissue. ***Peridium*** 15–45 μm wide, 5–8 layers, brown to dark brown cells of *textura angularis*. ***Hamathecium*** comprising 2–8 μm wide, oblong to cylindrical, septate, unbranched, cellular, paraphyses. ***Asci*** 55–90 × 5–6.5 µm (x̄ = 75 × 6.2 µm, n = 30), 8- or 6-spored, unitunicate, cylindrical, short pedicellate, persistent, apically rounded, with amyloid, cuboid, apical apparatus, staining blue in Melzer’s reagent, 2–3 µm high × 1.4–2.44 μm wide (x̄ = 2.6 × 1.9 μm). ***Ascospores*** 10–13.5 × 4–6 µm (x̄ = 12 × 5 μm, n = 30), uniseriate, unicellular, hyaline when immature, dark brown at maturity, aseptate, equilateral ellipsoid, with rounded ends, smooth, guttulate, with a straight germ slit nearly full-length, surrounded by a sheath. ***Asexual morph***: undetermined.

**Figure 2.**
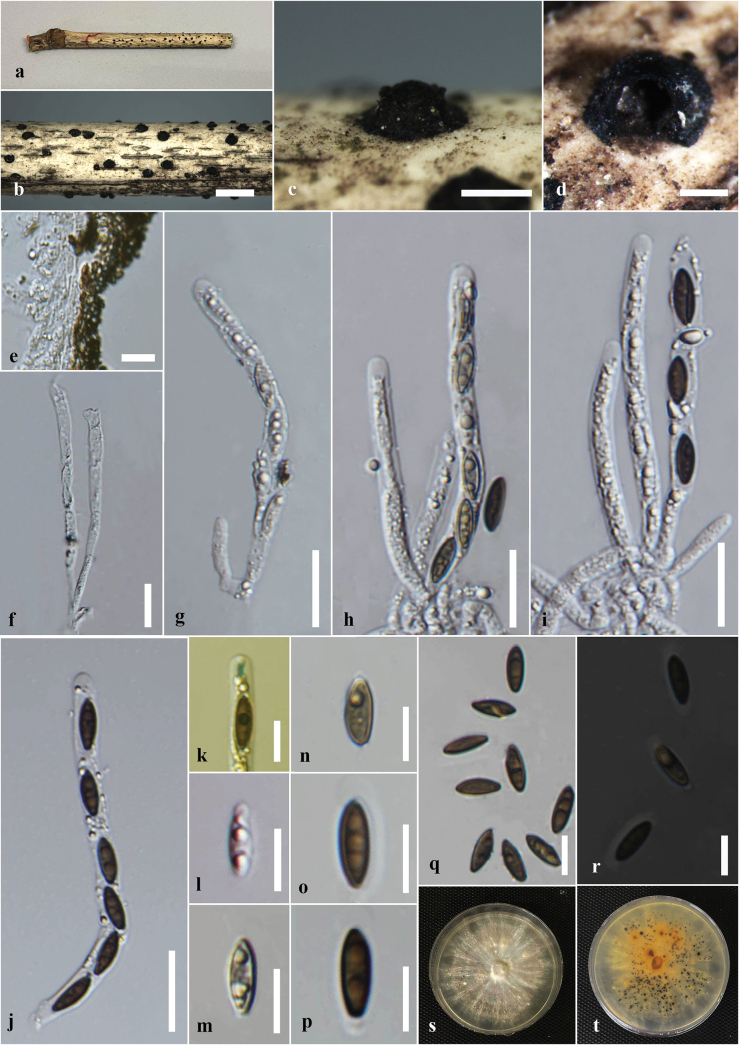
*Astrocystisbambusae* on a dead twig of *Bambusavulgaris* (MFLU 24-0522, a new host record). **a.** Substrate; **b, c.** Appearance of stromata on the host; **d.** Cross section of the stroma; **e.** Peridium; **f.** Paraphyses; **g–j.** Asci; **k.** Ascus apical apparatus (stained in Melzer’s reagent); **l–q.** Ascospores; **r.** Ascospores with sheath; **s, t.** Colony on the PDA (s upper, t lower). Scale bars: 5 mm (**b**); 1 mm (**c**); 500 μm (**d**); 20 μm (**f–j**); 10 μm (**e, k–r**).

#### Culture characteristics.

Ascospores germinated on the PDA within 24 hours at 25 °C. Germ tubes are produced from one side of the ascospore. Colonies on PDA reaching 2–2.5 cm diam. after 5 days at 25 °C, circular in shape, white at first, cottony, slightly thinning towards the edge, white color in the front view, and light brown in the reverse view.

#### Material examined.

Thailand • Chiang Rai, Mae Chan, Mae Chan District, on dead culms of *Bambusavulgaris* (Poaceae), 18 March 2024, Hsan Win (MFLU 24-0522), living culture MFLUCC 25-0022.

#### Known distribution and hosts.

China, India, Thailand (*Bambusa* sp.); Ghana (*Oxytenantheraabyssinica*); Philippines (*Bambusa* sp., *Schizostachyum* sp.) (Læssøe and Spooner 1993; Li et al. 2024); Thailand (*Bambusavulgaris*) (this study).

#### Notes.

Morphologically, our collection (MFLUCC 25-0022) exhibits characteristics similar to the holotype of *A.bambusae* (basionym: *Roselliniabambusae*) (Merrill 5030) and other isolates of *A.bambusae* (GMB0700). These similarities include scattered, solitary, superficial, black stromata containing one ascomata, with a circle of black tissue at the bottom and unitunicate, cylindrical, short pedicellate asci, with an apical apparatus that stains blue in Melzer’s reagent (Li et al. 2024). The ascospores have a straight germ slit nearly full-length and are surrounded by a sheath (Ju and Rogers 1990; Li et al. 2024). However, the asci and ascospores in our collection (MFLUCC 25-0022, 55–90 μm and 10–13.5 μm, respectively) are smaller than the holotype (100–130 μm and 10.5–15(–16) μm) (Ju and Rogers 1990). Based on multi-gene phylogenetic analyses (ITS, *β-tub*, and *rpb2*), our strain (MFLUCC 25-0022) clustered with other authentic strains (HAST 89021904 and GMB0700) in a well-supported clade (89% ML, 0.95 BYPP) (Fig. 1). *Astrocystisbambusae* has previously been recorded on *Bambusa* sp. in China, India, the Philippines, and Thailand (Ju and Rogers 1990; Læssøe and Spooner 1993; Li et al. 2024). In our study, we reported a new host record for *A.bambusae* on *Bambusavulgaris*.

### ﻿ Annulohypoxylon

Taxon classificationFungiAscomycotaXylariales

Y.M. Ju, J.D. Rogers & H.M. Hsieh, Mycologia 97(4): 855 (2005)

806C1BA3-7232-544D-A1F9-660E6FD1ED71

Index Fungorum: IF500298

Facesoffungi Number: FoF02983

#### Notes.

*Annulohypoxylon* was introduced with *A.truncatum* as the type species (Hsieh et al. 2005). Kuhnert et al. (2017) conducted a concise revision of this genus based on molecular phylogeny and chemotaxonomic data, resulting in the identification of several additional species, such as *A.massivum*, *A.violaceopigmentum*, *A.viridistratum*, and *A.yungensis*. *Annulohypoxylon* is characterized by effused-pulvinate or pulvinate, glomerate stromata, waxy or carbonaceous tissue immediately beneath the surface and between perithecia, spherical, obovoid, with a carbonaceous stromata layer surrounding individual perithecia. Asci are light- to dark-colored, 8-spored, cylindrical, stipitate, persistent, with discoid apical ring, amyloid or infrequently inamyloid, while ascospores are light- to dark-colored, ellipsoid or short fusoid, inequilateral, narrowly rounded, or with broadly rounded ends, with a germ slit, perispore dehiscent or indehiscent in 10% KOH (Li et al. 2016). *Annulohypoxylon* species have mainly been recorded in tropical and subtropical regions as saprobes associated with dead dicotyledonous wood and as endophytes in seed plants (Kuhnert et al. 2017). Hyde et al. (2024) listed 60 species under this genus, while 73 species are included in the Index Fungorum (2025).

##### ﻿Phylogenetic analyses for *Annulohypoxylon*

**Figure 3.**
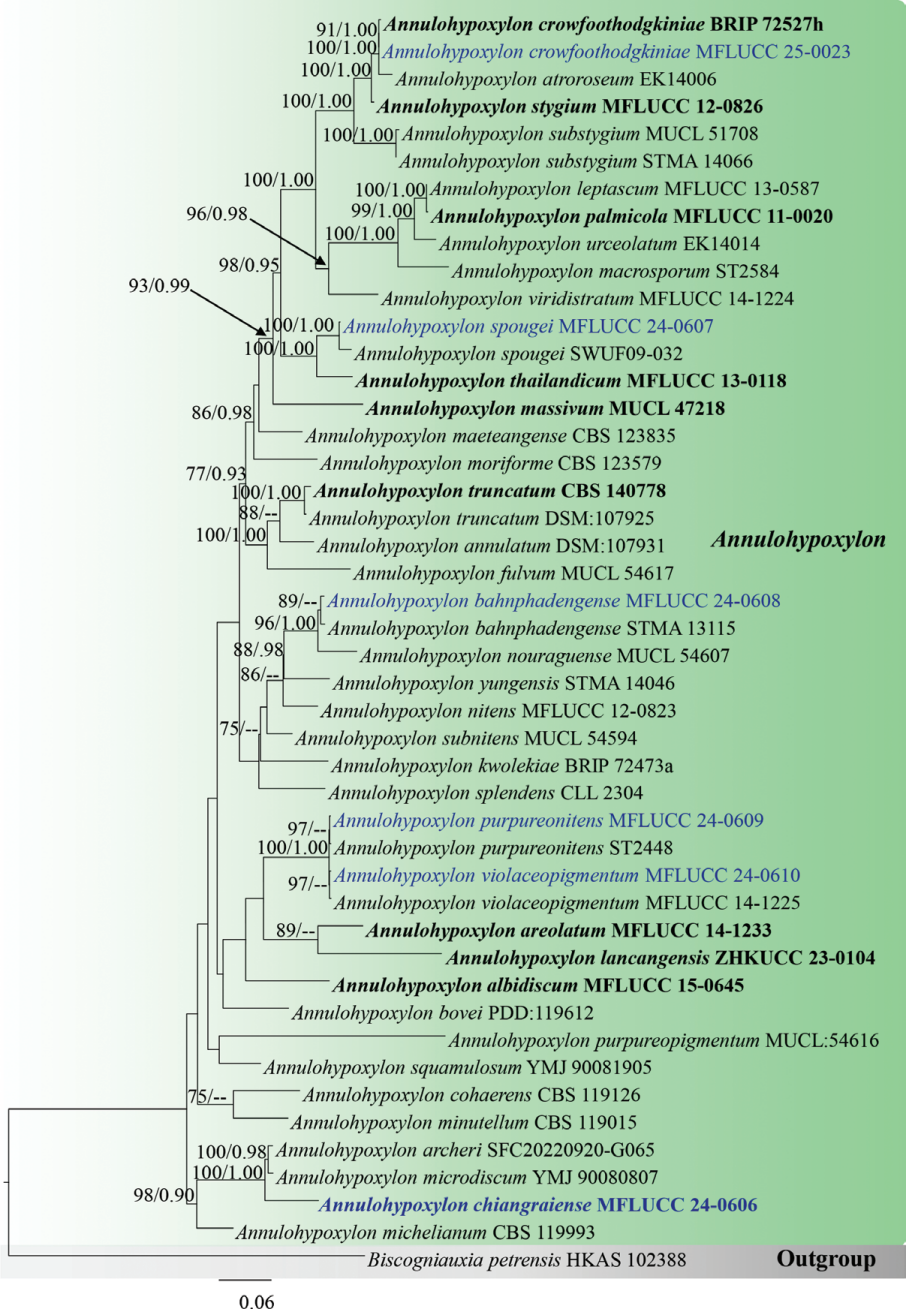
Phylogram generated from ML analysis based on the combined dataset of ITS, LSU, *β-tub*, and *rpb*2. The tree is rooted to *Biscogniauxiapetrensis* (HKAS102388). Bootstrap support values for ML ≥ 70% and Bayesian posterior probabilities (PP) ≥ 0.90 are noted at the nodes. Strain numbers are noted after the species names. Strains isolated in this study are represented in blue, and type strains are in bold.

Forty-six taxa of *Annulohypoxylon* were included in the combined data set (ITS, LSU, *β-tub*, and *rpb*2) with *Biscogniauxiapetrensis* (HKAS102388) as the outgroup taxon. After alignment, the dataset comprised 2832 characters, including gaps (ITS = 585 bp, LSU = 850 bp, *β-tub* = 380 bp, *rpb*2 = 1017 bp). The tree topology of the BI analysis (not shown) was similar to the ML tree. The best-scoring RAxML tree was obtained (Fig. 3), with a final likelihood value of -19758.666775. The matrix had 1149 distinct alignment patterns with 46.81% undetermined characters or gaps. Estimated base frequencies were as follows: A = 0.248997, C = 0.254092, G = 0.262808, T = 0.234103; substitution rates AC = 1.551284, AG = 4.096946, AT = 1.717695, CG = 1.101636, CT = 7.150044, GT = 1.0; gamma distribution shape parameter α = 0.220052. In BI analyses, the average standard deviation of split frequencies was 0.008 after 3,000,000 generations of runs. The phylogenetic tree topology is similar to that by Kuhnert et al. (2017). Our strains (MFLUCC 24-0606, MFLUCC 24-0607, MFLUCC 24-0608, MFLUCC 24-0609, MFLUCC 24-0610, and MFLUCC 25-0023) cluster within *Annulohypoxylon*.

###### ﻿Taxonomy

### ﻿ Annulohypoxylon bahnphadengense

Taxon classificationFungiAscomycotaXylariales

J. Fourn. & M. Stadler, Fungal Diversity 40: 30 (2010)

F08BA9AC-8591-5E4C-AF60-0F9CCAF2F19E

Index Fungorum: IF512545

Facesoffungi Number: FoF17293

Fig. 4

#### Description.

***Saprobic*** on the dead wood of *Berryacordifolia*. ***Sexual morph*: *Ascostromata*** 3–14 × 2–10 × 0.5–0.2 mm (x̄ = 7 × 6 × 0.3 mm, n = 8), effused-applanate, superficial, pulvinate to hemispherical, clustered, hard-textured, shiny, surface black, carbonaceous. ***Ascomata*** 0.5–3.5 mm high × 0.3–0.5 mm diam. (x̄ = 2 × 0.4 mm, n = 15), immersed in the stroma, subglobose to globose, black, ostiolate, papillate, encircled with a flattened truncatum-type disc 0.2–0.25 mm diam. (x̄ = 0.22 mm, n = 10). ***Peridium*** 40–60 μm wide, composed of several layers of hyaline to dark brown cells of *textura angularis*. ***Hamathecium*** 4–6 μm wide, comprising long, hyaline, unbranched, septate paraphyses. ***Asci*** 54–130 × 3–5 μm (x̄ = 94 × 4.5 μm, n = 20), 8-spored, unitunicate, cylindrical, short pedicellate, with an apical ring bluing in Melzer’s reagent. ***Ascospores*** 6–8 × 3–4 μm (x̄ = 7.5 × 3.5 μm, n = 40), uniseriate, one-celled, inequilaterally ellipsoidal, with narrowly rounded ends, hyaline when immature, becoming light brown to dark brown at maturity, guttulate. ***Asexual morph***: Undetermined.

**Figure 4.**
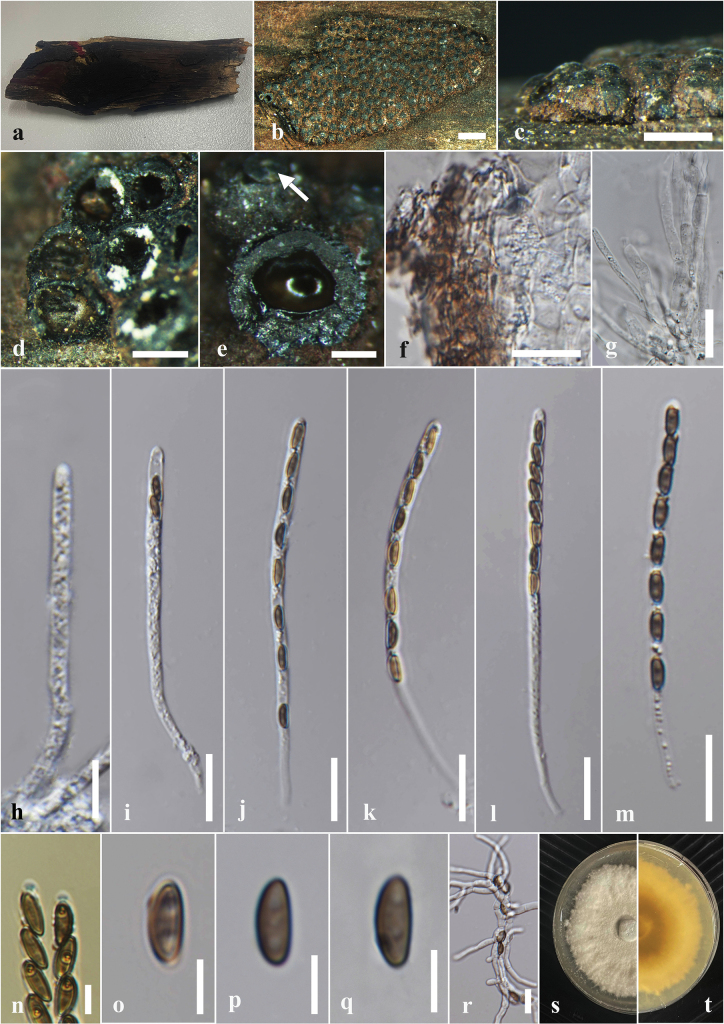
*Annulohypoxylonbahnphadengense* on dead wood of *Berryacordifolia* (MFLU 24-0526, a new host record). **a.** Substrate; **b, c.** Appearance of ascostromata on host; **d, e.** Horizontal section through ascomata (arrow shows the ostiolar disc); **f.** Peridium; **g.** Paraphyses; **h–m.** Asci; **n.** Apical apparatus stained blue with Melzer’s reagent; **o–q.** Ascospores; **r.** Germinated ascospores; **s, t.** Colony on the PDA (**s** upper, **t** lower). Scale bars: 2 mm (**b**); 1 mm (**c**); 500 μm (**d**); 200 μm (**e**); 20 μm (**f, g, i–m**); 10 μm (**h, r**); 5 μm (**n–q**).

#### Culture characters.

Ascospores germinated on the PDA within 24 hours at 25 °C. Germ tubes are produced from both sides of the ascospore. Colonies on the PDA reaching 2.0–2.5 cm diam. after six days at 25 °C, circular in shape, white at first, cottony, white color in the front view, brown in the middle, and pale brown at the margin in the reverse view.

#### Material examined.

Thailand • Chiang Rai, Phan District, Sai Khao, forest area near Wat Udom Waree, on decaying wood of *Berryacordifolia* (Malvaceae), 05 July 2024, Achala Rathnayaka, AA28 (MFLU 24-0526); living culture MFLUCC 24-0608.

#### Known distribution and hosts.

China (decaying wood) (Ke et al. 2024); Thailand (dead bark or wood, *Berryacordifolia*) (Fournier et al. 2010b; this study).

#### Notes.

Morphologically, our collection (MFLUCC 24-0608) shows similar characteristics to the holotype of *A.bahnphadengense* (MFU08-1552), including shiny, black, carbonaceous ascostromata; 8-spored, cylindrical, short-pedicellate asci with an apical ring bluing in Melzer’s reagent; and uniseriate, one-celled, inequilaterally ellipsoidal, ascospores with narrowly rounded ends (Fournier et al. 2010b). According to the multi-gene phylogenetic analyses (ITS, LSU, *β-tub*, and *rpb*2), our strain (MFLUCC 24-0608) clusters with the ex-type strain of *A.bahnphadengense* (STMA 13115) with 89% ML bootstrap and 0.84 PP support (Fig. 3). Based on the morpho-molecular evidence, we identified our collection as a new host record of *A.bahnphadengense* on *Berryacordifolia* in Thailand.

### ﻿ Annulohypoxylon chiangraiense

Taxon classificationFungiAscomycotaXylariales

Rathnayaka, K.D. Hyde & Chethana sp. nov.

187AE288-FC89-5D75-875E-1B77BCD88780

Index Fungorum number: IF903883

Facesoffungi Number: FoF17288

Fig. 5

#### Etymology.

The epithet chiangraiense refers to Chiang Rai Province, where the fungus was collected.

#### Holotype.

MFLU 24-0524.

#### Description.

***Saprobic*** on the dead branch of *Tamarindusindica*. ***Sexual morph*: *Ascostromata*** 0.4–0.6 × 0.8–1.5 mm (x̄ = 0.5 × 1.2 mm, n = 10), semi-immersed to superficial, with the base immersed, pulvinate to hemispherical, solitary or clustered, spherical surface black, carbonaceous. ***Ascomata*** immersed in stroma, globose to subglobose, black. ***Peridium*** 18–30 μm wide, composed of several layers of hyaline to dark brown cells of *textura angularis*. *Paraphyses* 3–6 μm wide, hyaline, abundant, persistent, unbranched, septate. ***Asci*** 90–145 × 8–5 μm (x̄ = 124 × 7 μm, n = 20), 8-spored, unitunicate, cylindrical, short pedicellate, with an apical ring not bluing in the Melzer’s reagent (without KOH pretreatment). ***Ascospores*** 10–12 × 4–7 μm (x̄ = 11 × 5 μm, n = 30), uniseriate, one-celled, inequilaterally ellipsoidal, with narrowly rounded ends, hyaline at immature stages, dark brown when mature, guttulate. ***Asexual morph***: Undetermined.

**Figure 5.**
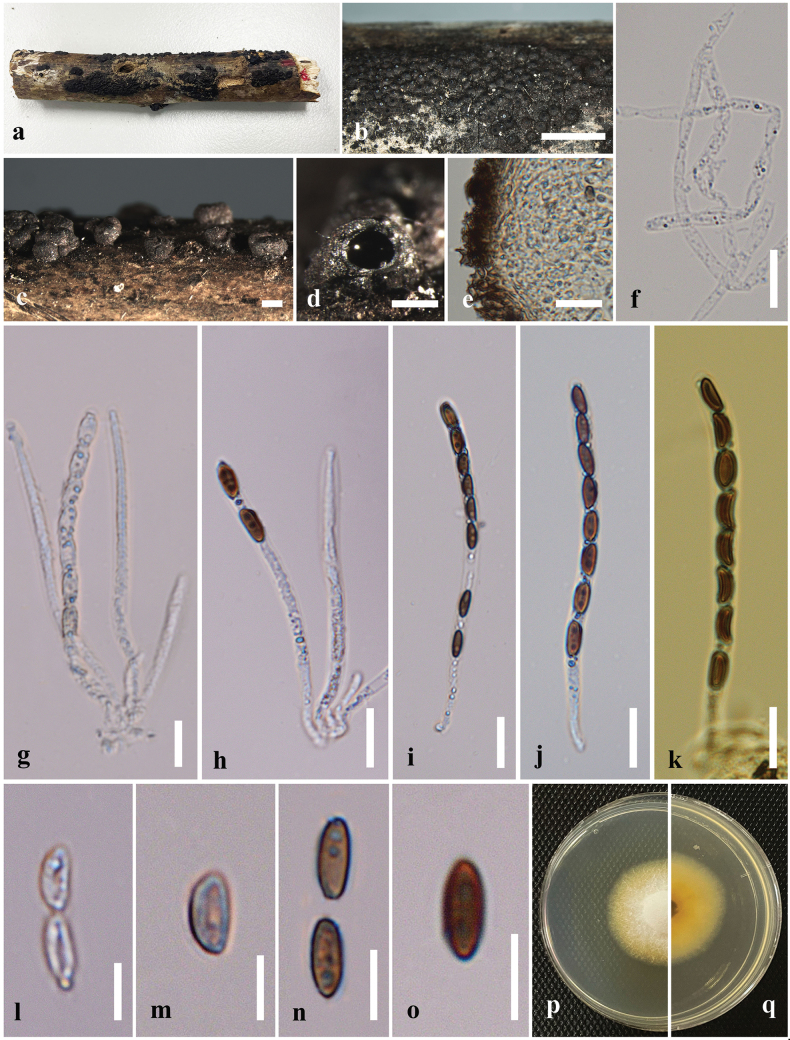
*Annulohypoxylonchiangraiense* on a dead branch of *Tamarindusindica* (MFLU 24-0524, Holotype). **a.** Sustrate; **b, c.** Stromata on the host; **d.** Cross section of the stroma; **e.** Peridium; **f.** Paraphyses; **g–j.** Asci; **k.** Ascal apical apparatus (not staining in Melzer’s reagent); **l–o.** Ascospores; **p, q.** Colony on the PDA (**p** upper, **q** lower). Scale bars: 5 mm (**b**); 1 mm (**c**); 500 μm (**d**); 20 μm (**e–k**); 10 μm (**l–o**).

#### Culture characters.

Ascospores germinated on the PDA within 24 hours at 25 °C. Germ tubes are produced from one side of the ascospore. Colonies on the PDA reaching 1.0–2.0 cm diam. after five days at 25 °C, circular in shape, white at first, cottony, slightly thinning towards the edge, with white color in the middle and pale yellow color in the margin of the front view, and pale yellow in the reverse view.

#### Material examined.

Thailand • near Nang Lae waterfall, Chiang Rai, on decaying wood of *Tamarindusindica* (Fabaceae), 18 March 2024, Achala Rathnayaka, AA11 (MFLU 24-0524, holotype); ex-type living culture, MFLUCC 24-0606.

#### Notes.

*Annulohypoxylon* is a speciose genus with more than 60 species; however, the present study shows the genus to be more diverse as predicted by Bhunjun et al. (2024). Based on the multi-gene phylogeny (ITS, LSU, *β-tub*, and *rpb*2), *Annulohypoxylonchiangraiense* (MFLUCC 24-0606) formed a distant lineage sister to *A.archeri* (SGNLB 5) and *A.microdiscum* (HMAS 285320) with 100% ML bootstrap and 1.00 PP support (Fig. 3). *Annulohypoxylonchiangraiense* fits within the generic concept of *Annulohypoxylon* by having spherical, carbonaceous ascostromata; 8-spored, cylindrical asci; and ellipsoid, light- to dark-brown ascospores (Li et al. 2016). *Annulohypoxylonchiangraiense* differs from both *A.archeri* and *A.microdiscum* by having smaller ascostromata (0.4–0.6 × 0.8–1.5 mm vs. 8–20 × 5–10 mm and 0.5–4 × 0.3–2 cm) (Raei et al. 2012; Cruz et al. 2020). The asci of *A.chiangraiense* are shorter and wider (90–145 × 8–5 μm) than *A.microdiscum* (130–187 × 5–6.5 μm). However, asci were not observed in *A.archeri* (Cruz et al. 2020). In *A.chiangraiense*, the apical ring does not turn blue in Melzer’s iodine reagent, whereas in *A.microdiscum*, the apical ring turns blue in Melzer’s iodine reagent (Raei et al. 2012). While ascospores of both *A.archeri* and *A.microdiscum* have a straight germ slit, such a character was not observed in the ascospores of *A.chiangraiense* (Raei et al. 2012; Cruz et al. 2020). When comparing the ITS base pair differences of *A.chiangraiense* with *A.archeri* and *A.microdiscum*, it shows 1.6% (8/564) and 1.9% (10/533) differences (without gaps), respectively. Based on the distinct morphology and phylogenetic evidence, we established *Annulohypoxylonchiangraiense* as a new species.

### ﻿ Annulohypoxylon crowfoothodgkiniae

Taxon classificationFungiAscomycotaXylariales

Y.P. Tan, Bishop-Hurley, Bransgr. & R.G. Shivas, Index of Australian Fungi 1: 1 (2022)

3BF3D690-588C-50BE-8D58-39E4C3204566

Index Fungorum: IF900010

Facesoffungi Number: FoF17294

Fig. 6

#### Description.

***Saprobic*** on dead wood of *Swieteniamacrophylla*. ***Sexual morph*: *Ascostromata*** 0.5–0.7 × 0.3–0.6 mm (x̄ = 0.6 × 0.5 mm, n = 10), semi-immersed to superficial, with the base immersed, pulvinate to hemispherical, clustered, shiny, surface black, carbonaceous. ***Ascomata*** immersed in the stroma, subglobose to hemispherical, black, ostiolate, papillate. ***Hamathecium*** 3–7 μm wide, comprising long, hyaline, unbranched, aseptate paraphyses. ***Asci*** 55–65 × 4–6 μm (x̄ = 60 × 4.5 μm, n = 20), 8-spored, unitunicate, cylindrical, short pedicellate, with an apical ring not bluing in the Melzer’s reagent. ***Ascospores*** 5–6 × 2–3 μm (x̄ = 5.4 × 2.8 μm, n = 30), uniseriate, one-celled, ellipsoidal inequilaterally, with narrowly rounded ends, hyaline when immature, becoming brown at maturity. ***Asexual morph***: Undetermined.

**Figure 6.**
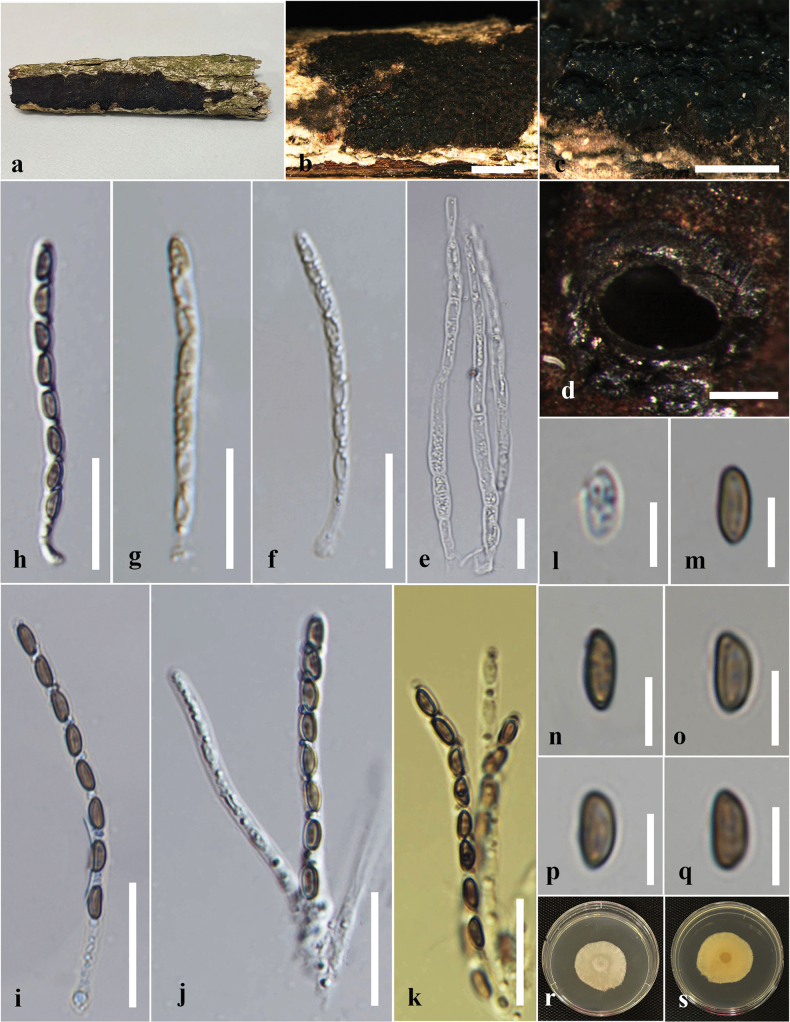
*Annulohypoxyloncrowfoothodgkiniae* on decaying wood of *Swieteniamacrophylla* (MFLU 24-0522, a new host and geographical record). **a.** Substrate; **b, c.** Stromata on the host; **d.** Cross section of the stroma; **e.** Paraphyses; **f–j.** Asci; **k.** Ascus apical apparatus (not stained in Melzer’s reagent); **l–q.** Ascospores; **r, s.** Colony on PDA (**r** upper, **s** lower). Scale bars: 5 mm (**b**); 1 mm (**c**); 200 μm (**d**); 20 μm (**e–k**); 5 μm (**l–q**).

#### Culture characters.

Ascospores germinated on the PDA within 24 hours at 25 °C. Germ tubes are produced from one side of the ascospore. Colonies on the PDA reaching 2.0–2.5 cm diam. after five days at 25 °C, circular in shape, white at first, cottony, slightly thinning towards the edge, white color in the front view, and light brown in the reverse view.

#### Material examined.

Thailand • Nang Lae Village, Chiang Rai, on decaying wood of *Swieteniamacrophylla* (Meliaceae), 21 May 2024, Zaw Lin Tun, AZ2 (MFLU 24-0523); living culture MFLUCC 25-0023.

#### Known distribution and hosts.

Australia (*Pandanustectorius*) (Tan and Shivas 2022a); Thailand (*Swieteniamacrophylla*) (this study).

#### Notes.

In the multi-gene phylogeny (ITS, LSU, *β-tub*, and *rpb*2), our strain (MFLUCC 25-0023) and the ex-type strain of *A.crowfoothodgkiniae* (BRIP 72527 h) clustered with 91% ML bootstrap and 1.00 PP support (Fig. 3). When comparing the base pair differences between our strain (MFLUCC 25-0023) and the ex-type strain of *A.crowfoothodgkiniae* (BRIP 72527 h), the ITS shows a 0.3% (3/894) difference, and there are no differences in LSU. The morphology of the holotype is not recorded. Therefore, we could not compare the morphology between the holotype and our strain. In here, we provide complete morphology with an illustration for *A.crowfoothodgkiniae*. Based on molecular evidence, we introduce our collection as a new host record of *A.crowfoothodgkiniae* from *Swieteniamacrophylla* and also as a new geographical record from Thailand.

### ﻿ Annulohypoxylon spougei

Taxon classificationFungiAscomycotaXylariales

Suwannasai, M.P. Martín, Phosri & Whalley, Persoonia 44: 353 (2020)

5CC6F3EF-0165-5012-B3A7-96BFD7DB8A69

Index Fungorum: IF811164

Facesoffungi Number: FoF17295

Fig. 7

#### Description.

***Saprobic*** on *Antidesmamadagascariense* dead wood. ***Sexual morph*: *Ascostromata*** 1–3 cm long × 0.3–2 cm broad and 0.8–1.2 mm thick (x̄ = 2 × 1.4 × 1 mm, n = 10), hemispherical, effused-pulvinate, shiny, surface black, carbonaceous. ***Ascomata*** 0.25–0.6 mm high × 0.25–0.5 mm diam. (x̄ = 0.4 × 0.3 mm, n = 10), immersed in the stroma, subglobose to globose, black, ostioles papillate, encircled with a flattened, truncatum-type disc, 0.2–0.25 mm diam. (x̄ = 0.23 mm, n = 8). ***Hamathecium*** 3–5 μm wide, comprising long, hyaline, unbranched, septate paraphyses. ***Asci*** 27–42 × 2–3 μm (x̄ = 36 × 2 μm, n = 20), the spore-bearing parts 17–25 µm long with stipes 9–15 µm long, 8-spored, unitunicate, cylindrical, with an apical ring bluing in Melzer’s iodine reagent. ***Ascospores*** 7–9 × 3–4 μm (x̄ = 7.6 × 3.6 μm, n = 40), uniseriate, one-celled, inequilaterally ellipsoidal, with narrowly rounded ends, hyaline when immature, becoming brown at maturity, guttulate. ***Asexual morph***: Undetermined.

**Figure 7.**
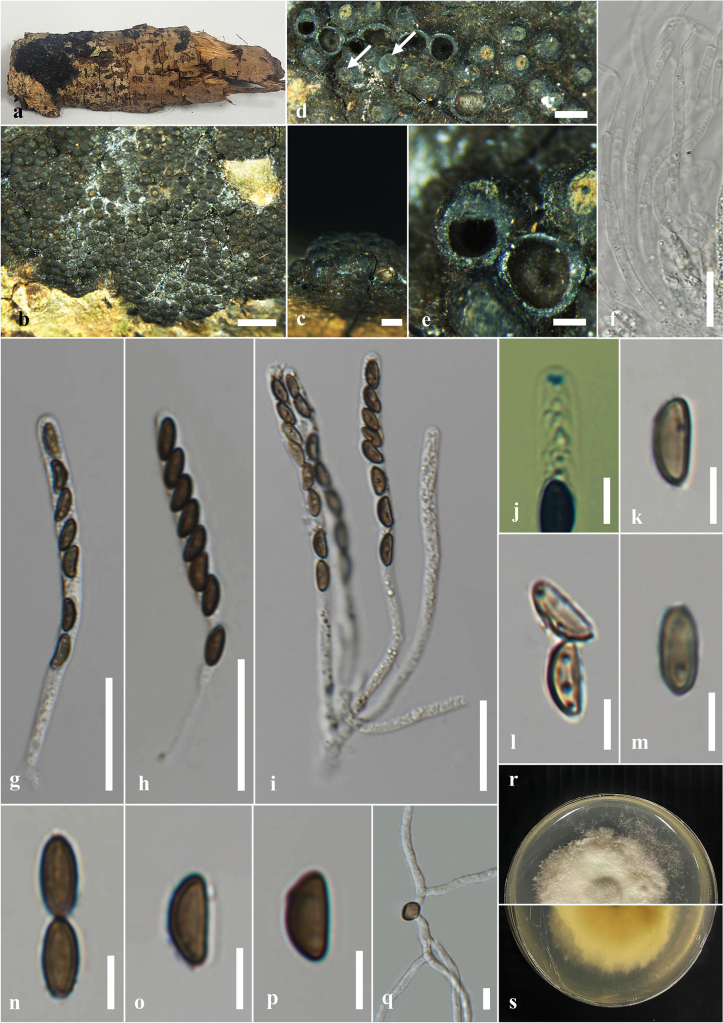
*Annulohypoxylonspougei* on dead wood of *Antidesmamadagascariense* (MFLU 24-0526, a new host record). **a.** Substrate; **b, c.** Appearance of ascostromata on the host; **d.** Ostiolar discs in ascomata (indicated by arrows); **e.** Horizontal section through the ascomata; **f.** Paraphyses; **g-i.** Asci; **j.** Apical apparatus stained blue with Melzer’s reagent; **k-p.** Ascospores; **q.** Germinated ascospores; **r, s.** Colony on the PDA (**r** upper, **s** lower). Scale bars: 2 mm (**b**); 500 μm (**c, d**); 200 μm (**e**); 20 μm (**f**); 10 μm (**g–i, q**); 5 μm (**j–p**).

#### Culture characters.

Ascospores germinated on the PDA within 24 hours at 25 °C. Germ tubes are produced from both sides of the ascospore. Colonies on the PDA reaching 1.5–2.0 cm diam. after five days at 25 °C, circular in shape, white at first, cottony, white color in the front view, brown in the middle, and pale brown at the margin of the reverse view.

#### Material examined.

Thailand • Chiang Rai, near Ang Kep Nam Huai Luang Than Thong Reservoir, on decaying wood of *Antidesmamadagascariense* (Phyllanthaceae), 05 July 2024, Achala Rathnayaka, AA24 (MFLU 24-0525); living culture MFLUCC 24-0607.

#### Known distribution and hosts.

China (rotten wood) (Ke et al. 2024); Thailand (on corticated wood, *Antidesmamadagascariense*) (Crous et al. 2020; this study).

#### Notes.

According to the multi-gene phylogenetic analyses (ITS, LSU, *β-tub*, and *rpb*2), our strain (MFLUCC 24-0607) clustered with the ex-type strain of *A.spougei* (SWUF09-032) with 100% ML bootstrap and 1.00 PP support (Fig. 2). Our fungal collection (MFLUCC 24-0607) exhibits morphological characteristics similar to the holotype of *A.spougei* (SWUFH099), including black, shiny carbonaceous ascostromata; 8-spored, unitunicate, cylindrical asci, with an apical ring that bluing in Melzer’s iodine reagent; and unicellular, inequilaterally ellipsoidal, brown ascospores (Crous et al. 2020). However, the ascospores of the *A.spougei* holotype show a straight germ slit along the full length of the spore, which is not observed in our isolate (MFLUCC 24-0607). Based on the morpho-molecular evidence, we identified our collection as a new host record of *A.spougei* on *Antidesmamadagascariense* in Thailand.

### ﻿ Annulohypoxylon purpureonitens

Taxon classificationFungiAscomycotaXylariales

(Y.M. Ju & J.D. Rogers) Y.M. Ju, J.D. Rogers & H.M. Hsieh, Mycologia 97(4): 861 (2005)

2147B750-CED2-549B-A177-6B263B9FD47B

Index Fungorum: IF500323

Facesoffungi Number: FoF17296

Fig. 8

Hypoxylon purpureonitens Y.M. Ju & J.D. Rogers 1996. Basionym.

#### Description.

***Saprobic*** on the dead wood of *Sterculiatragacantha*. ***Sexual morph*: *Ascostromata*** 2–10 mm long × 3–8 mm broad and 0.25–0.35 mm thick (x̄ = 7.5 × 5.5 × 0.3 mm, n = 10), hemispherical, effused-pulvinate, solitary or clustered, shiny, surface black, carbonaceous. ***Ascomata*** 0.4–0.5 mm high × 0.3–0.4 mm diam. (x̄ = 0.45 × 0.35 mm, n = 15), immersed in the stroma, subglobose to globose, black, ostioles papillate, encircled with a flattened truncatum-type disc 0.18–0.25 mm diam. (x̄ = 0.2 mm, n = 10). ***Hamathecium*** 2.5–4.5 μm wide, comprising long, hyaline, unbranched, aseptate paraphyses. ***Asci*** 90–110 × 5–7 μm (x̄ = 100 × 6.2 μm, n = 20), the spore-bearing parts 60–70 µm long with stipes 20–40 µm long, 6-spored, unitunicate, cylindrical, with an apical ring not bluing in Melzer’s iodine reagent. ***Ascospores*** 7–9 × 3–6 μm (x̄ = 8.5 × 4.4 μm, n = 40), uniseriate, one-celled, inequilaterally ellipsoidal, with narrowly rounded ends, hyaline when immature, becoming dark brown at maturity, guttules present at the immature stage, with straight, spore length germ slit. ***Asexual morph***: Undetermined.

**Figure 8.**
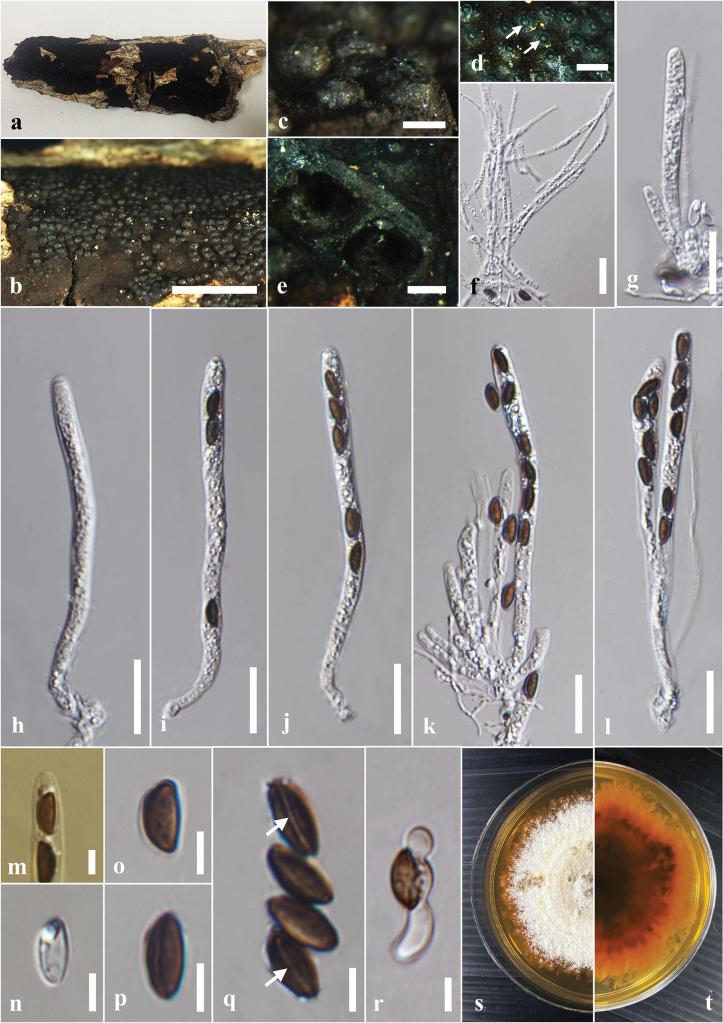
*Annulohypoxylonpurpureonitens* on the dead wood of *Sterculiatragacantha* (MFLU 24-0527, a new host record). **a.** Substrate; **b, c.** Appearance of ascostromata on host; **d.** Ostiolar discs in ascomata (indicated by arrows); **e.** Horizontal section through ascomata; **f.** Paraphyses; **g–l.** Asci; **m.** Apical apparatus (not staining in Melzer’s reagent); **n–q.** Ascospores (**q**: arrows indicate the germ slit); **r** Germinated ascospores; **s, t** Colony on the PDA (**s** upper, **t** lower). Scale bars: 5 mm (**b**); 500 μm (**c, d**); 200 μm (**e**); 20 μm (**f–l**); 5 μm (**m–r**).

#### Culture characters.

Ascospores germinated on the PDA within 24 hours at 25 °C. Germ tubes are produced from both sides of the ascospore. Colonies on the PDA reaching 2–2.5 cm diam. after seven days at 25 °C, circular in shape, white at first, cottony, white color in the front view, dark brown or black in the middle, and brown at the margin in the reverse view.

#### Material examined.

Thailand • Chiang Rai, Phan District, Sai Khao, forest area near Wat Udom Waree, on decaying wood of *Sterculiatragacantha* (Malvaceae), 05 July 2024, Achala Rathnayaka, AA30 (MFLU 24-0527); living culture MFLUCC 24-0609.

#### Known distribution.

Brazil (on an unidentified branch of a dicotyledonous tree) (Pereira et al. 2009); Thailand (on rotten wood, *Sterculiatragacantha*) (Suwannasai et al. 2013; this study).

#### Notes.

According to the multi-gene phylogenetic analyses (ITS, LSU, *β-tub*, and *rpb*2), our strain (MFLUCC 24-0609) clustered with the ex-type strain of *A.purpureonitens* (MFLUCC 14-1225) with 97% ML bootstrap and 0.84 PP support (Fig. 3). Our fungal collection (MFLUCC 24-0609) shows morphological characteristics similar to the holotype of *A.purpureonitens* (WSP 71615), including effused-pulvinate ascostromata and unicellular, ellipsoid-inequilateral, brown ascospores with a straight, spore-length germ slit (Pereira et al. 2009). In this study, we introduced our fungal collection as a new host record of *A.purpureonitens* on *Sterculiatragacantha* in Thailand.

### ﻿ Annulohypoxylon violaceopigmentum

Taxon classificationFungiAscomycotaXylariales

Sir & Kuhnert, Fungal Diversity: 10.1007/s13225-016-0377-6, [9] (2016)

D28D608A-AFA8-5EC4-834A-57EDF81E1D20

Index Fungorum: IF552341

Facesoffungi Number: FoF02507

Fig. 9

#### Description.

***Saprobic*** on the dead wood of *Syzygiumpolyanthum*. ***Sexual morph*: *Ascostromata*** 8–15 mm long × 5–10 mm broad and 0.5–1.2 mm thick (x̄ = 12 × 5.5 × 0.8 mm, n = 10), hemispherical, effused-pulvinate, clustered, developing within cuticle, surface black, carbonaceous. ***Ascomata*** 0.4–0.5 mm high × 0.3–0.4 mm diam. (x̄ = 0.45 × 0.35 mm, n = 10), immersed in the stroma, subglobose to globose, black, ostioles papillate, encircled with a flattened truncatum-type disc 0.2–0.25 mm diam. (x̄ = 0.24 mm, n = 5). ***Hamathecium*** 1–2 μm wide, comprising long, hyaline, unbranched, aseptate paraphyses. ***Asci*** 74–110 × 5–7 μm (x̄ = 97 × 6.7 μm, n = 20), the spore-bearing parts 70–77 µm long with stipes 29–32 µm long, 8-spored, unitunicate, cylindrical, with an apical ring not bluing in Melzer’s iodine reagent. ***Ascospores*** 7–10 × 4–6 μm (x̄ = 8.7 × 4.6 μm, n = 40), uniseriate, one-celled, inequilaterally ellipsoidal with narrowly rounded ends, hyaline when immature, becoming dark brown at maturity, guttules at immature stage, with a spore length straight germ slit. ***Asexual morph***: Undetermined.

**Figure 9.**
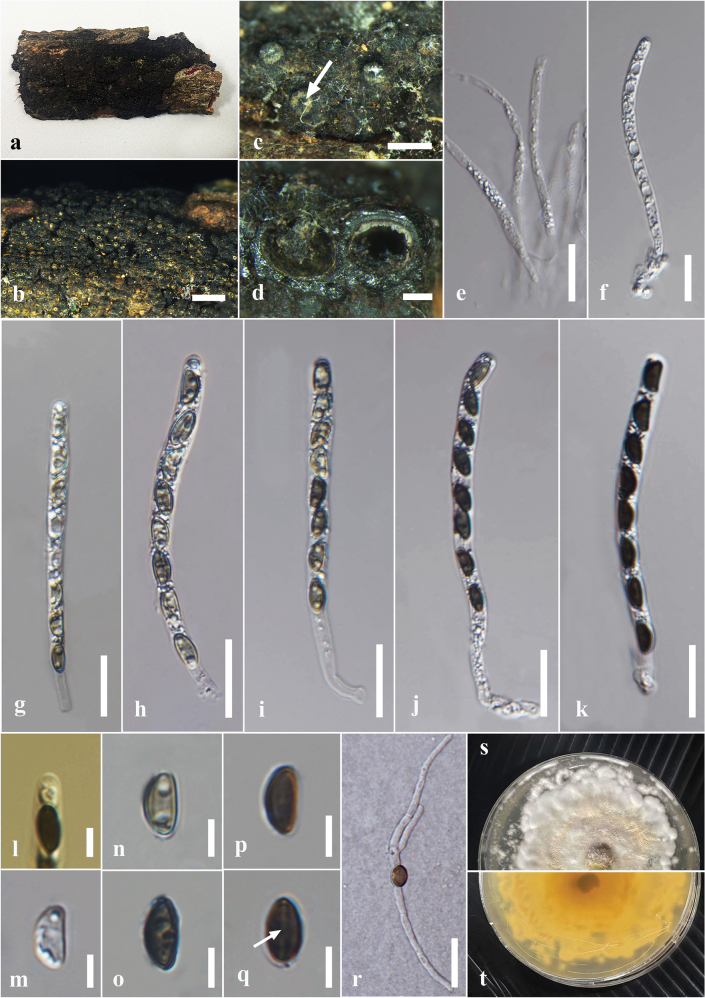
*Annulohypoxylonviolaceopigmentum* on the dead wood of *Syzygiumpolyanthum* (MFLU 24-0528, a new host record). **a.** Substrate; **b, c.** Appearance of ascomata on the host (ostiolar discs indicated by the arrow); **d.** Horizontal section through ascomata; **e.** Paraphyses; **f–k.** Asci; **l.** Ascus apical apparatus (not stained in Melzer’s reagent); **m–q.** Ascospores (**q**: arrow indicates the germ slit); **r** A germinated ascospore; **s, t** Colony on the PDA (**s** upper, **t** lower). Scale bars: 2 mm (**b**); 500 μm (**c**); 200 μm (**d**); 20 μm (**e–k, r**); 5 μm (**l–q**).

#### Culture characters.

Ascospores germinated on the PDA within 24 hours at 25 °C. Germ tubes are produced from both sides of the ascospore. Colonies on the PDA reaching 2–2.5 cm diam. after five days at 25 °C, circular in shape, white at first, cottony, white color in the front view, brown in the middle, and pale brown at the margin in the reverse view.

#### Material examined.

Thailand • Chiang Rai, Phan District, Sai Khao, forest area near Wat Udom Waree, on decaying wood of *Syzygiumpolyanthum* (Myrtaceae), 05 July 2024, Achala Rathnayaka, AA31 (MFLU 24-0528); living culture MFLUCC 24-0610.

#### Known distribution and hosts.

Thailand (on dead wood, *Syzygiumpolyanthum*) (Kuhnert et al. 2017; this study).

#### Notes.

The morphological description of our collection (MFLUCC 24-0610) aligns with the holotype of *A.violaceopigmentum* (MFLU 14-0314), including effused-pulvinate ascostromata; black, ostioles, papillate ascomata encircled by a flattened truncatum-type disc; 8-spored, cylindrical asci; and brown, unicellular, inequilaterally ellipsoidal ascospores with broadly rounded ends and a straight germ slit along the spore length (Kuhnert et al. 2017). Based on the multi-gene phylogenetic analyses (ITS, LSU, *β-tub*, and *rpb*2), our strain (MFLUCC 24-0610) clusters with the ex-type strain of *A.violaceopigmentum* (MFLUCC 14-1225) with 97% ML bootstrap and 0.85 PP support (Fig. 3). Considering the morpho-molecular evidence, we conclude that our collection is a new host record of *A.violaceopigmentum* on *Syzygiumpolyanthum* in Thailand.

### ﻿ Halorosellinia

Taxon classificationFungiAscomycotaXylariales

Whalley, E.B.G. Jones, K.D. Hyde & Læssøe, Mycol. Res. 104(3): 368 (2000)

63FA1191-BB72-5099-A27F-5311F6CD8849

Index Fungorum: IF28368

Facesoffungi Number: FoF03045

#### Notes.

*Halorosellinia* was introduced by Whalley et al. (1999) as a monotypic genus to accommodate *H.oceanica* (previously referred to as *Hypoxylonoceanicum*). This genus is characterized by uniperitheciate ascomata immersed in a pseudostroma (Hyde et al. 2016). *Halorosellinia* currently comprises five species (Index Fungorum 2025). Only three *Halorosellinia* species are included in Wijayawardene et al. (2022), while five species are listed in the Index Fungorum (2025) and Hyde et al. (2024).

##### ﻿Phylogenetic analyses for *Xylariaceae*

**Figure 10.**
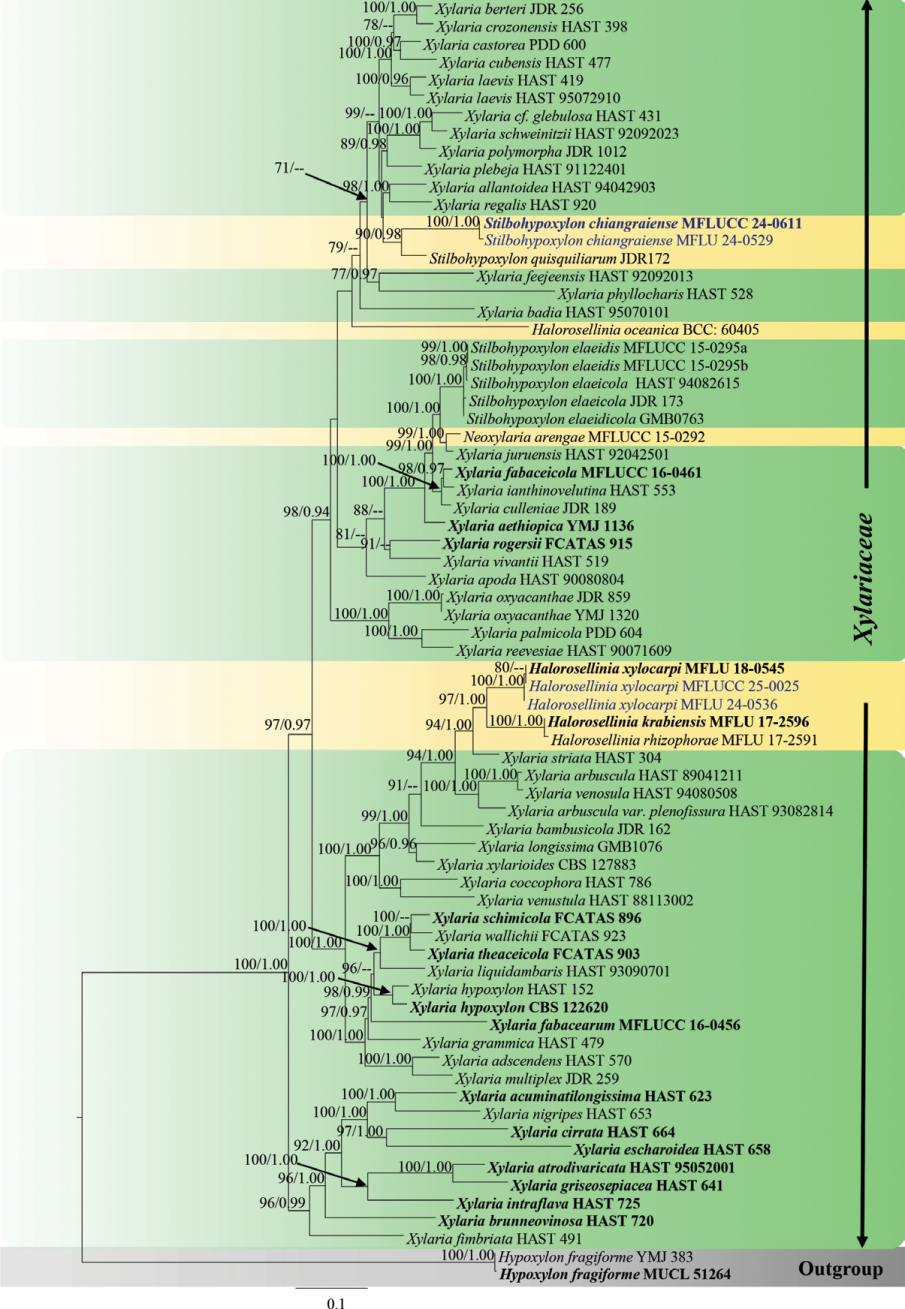
Phylogram generated from ML analysis based on the combined dataset of ITS, *rpb*2, and *β-tub*. The tree is rooted to *Hypoxylonfragiforme* (HAST 383 and MUCL 51264). Bootstrap support values for ML ≥ 70% and Bayesian posterior probabilities (PP) ≥ 0.90 are noted at the nodes. Strain numbers are noted after the species names. Strains isolated in this study are represented in blue, and type strains are in bold.

For Xylariaceae, the ITS, *rpb*2, and *β-tub* gene regions were used in the combined data set. Seventy-two isolates of Xylariaceae species were included in the analysis, with *Hypoxylonfragiforme* (HAST 383 and MUCL 51264) as the outgroup taxa. After alignment, the dataset comprises 2747 characters, including gaps (ITS = 580 bp, *rpb*2 = 1122 bp, *β-tub* = 1045 bp). The topology of the BI tree was similar to that of the ML tree. The best-scoring RAxML tree, with a final likelihood value of -49702.0557, is shown in Fig. 10. The matrix comprises 1637 distinct alignment patterns, with 16.93% undetermined characters or gaps. Estimated base frequencies were as follows: A = 0.243971, C = 0.271335, G = 0.241282, and T = 0.243412; substitution rates were AC = 1.350468, AG = 5.371719, AT = 1.169242, CG = 1.195494, CT = 7.102768, and GT = 1.0; and the gamma distribution shape parameter α = 0.335659. In the BI analysis, the average standard deviation of split frequencies was 0.01 after 3,000,000 generations of runs. The phylogenetic tree topology is similar to the study by Konta et al. (2020a). According to the phylogenetic analyses, our strains (MFLU 24-0536 and MFLUCC 25-0025) cluster with *Haloroselliniaxylocarpi* (MFLU 18-0545) with 100% ML bootstrap and 1.00 PP support, while MFLUCC 24-0611 clusters sister to *Stilbohypoxylonquisquiliarum* (YMJ 172) with 90% ML bootstrap and 0.98 PP support (Fig. 10).

###### ﻿Taxonomy

### ﻿ Halorosellinia xylocarpi

Taxon classificationFungiAscomycotaXylariales

Dayar & K.D. Hyde, Mycosphere 11(1): 158 (2020)

D67773CC-30AE-5878-9C0F-DB4DD040DBEC

Index Fungorum number: IF556600

Facesoffungi Number: FoF06192

Fig. 11

#### Description.

***Saprobic*** on decaying submerged wood of Arecaceae sp. ***Sexual morph*: *Pseudostromata*** 0.6–1.0 × 0.5–0.8 mm (x̄ = 0.8 × 0.6 mm, n = 5), superficial, pulvinate to hemispherical, in clusters of uni-peritheciate pseudostromata, surface black, carbonaceous, lacking ascomatal projections. ***Ascomata*** 0.3–0.34 × 0.34–0.36 mm (x̄ = 0.33 × 0.35 mm, n = 5), superficial, globose or subglobose to hemispherical, black, ostioles papillate. ***Peridium*** 25–38 μm wide, consists of 6–7 layers of brown to dark brown *textura angularis* cells. ***Paraphyses*** 4–8 μm wide, hyaline, abundant, persistent, unbranched, septate. ***Asci*** 85–115 × 9–17 μm (x̄ = 97 × 12 μm, n = 20), 8-spored, unitunicate, cylindrical, long pedicellate, with J+, cylindrical apical ring. ***Ascospores*** 13–17 × 7–9 μm (x̄ = 15 × 8 μm, n = 30), overlapping 1–2-seriate, hyaline, becoming opaque green and dark brown when mature, more or less equilaterally ellipsoid, straight, both ends often pointed, 1-celled, guttulate, without appendages, a spore length germ slit on the ventral side, straight. ***Asexual morph***: Undetermined.

**Figure 11.**
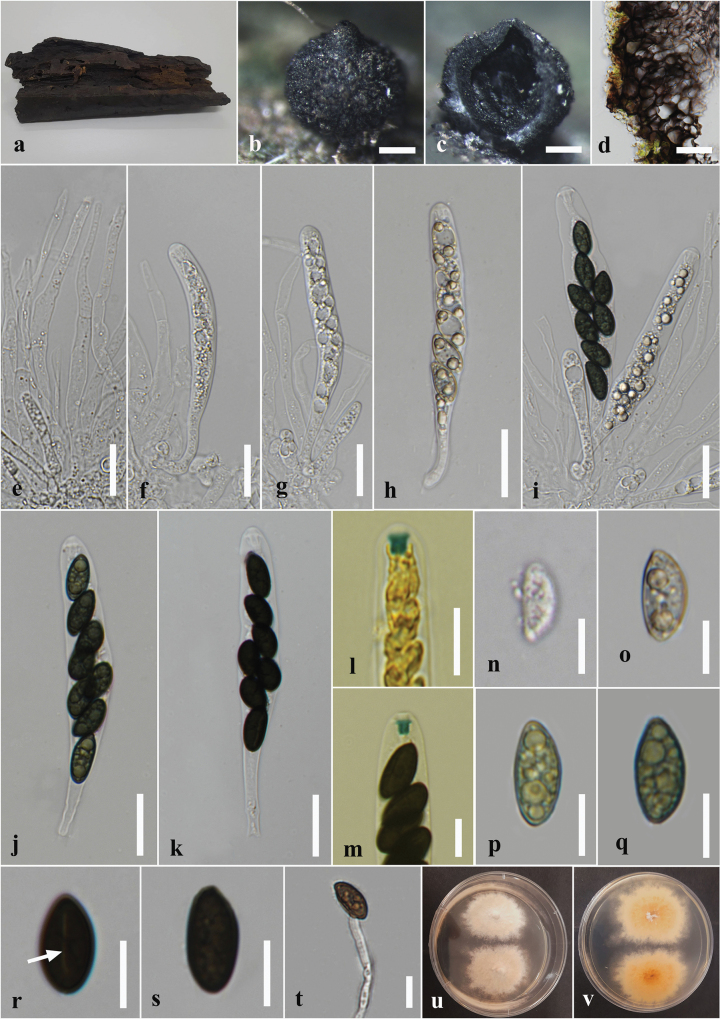
*Haloroselliniaxylocarpi* on decaying submerged wood of Arecaceae sp. (MFLU 24-0536, a new host record). **a.** Substrate; **b.** Appearance of an ascoma on the host; **c.** A horizontal section through an ascoma; **d.** Peridium; **e.** Paraphyses; **f–k.** Asci; **l, m.** Apical apparatus stained blue with Melzer’s reagent; **n–s.** Ascospores (**r**: arrow shows the germ slit on the ventral side); **t** A germinated ascospore; **u, v** Colony on the PDA (**u** upper, **v** lower). Scale bars: 100 μm (**b, c**); 20 μm (**d–k**); 10 μm (**l–t**).

#### Culture characteristics.

Ascospores germinated on the PDA within 24 hours at 25 °C. Germ tubes are produced from one side of the ascospore. Colonies on the PDA at 25–28 °C, reaching 6 cm in seven days, circular in shape, zonate with diffused margins, white color in front view, and pale yellow in reverse view.

#### Material examined.

Thailand • Chang Wat Prachuap Khiri Khan Province, Pran Buri District, Pran Buri riverbank, 26 February 2023, Tharindu Bhagya, on decaying submerged wood of Arecaceae sp., TB (MFLU 24-0536), living culture, MFLUCC 25-0025.

#### Known distribution and hosts.

Thailand (submerged wood of *Xylocarpus* sp., *Rhizophora* sp., and submerged wood of Arecaceae sp.) (Dayarathne et al. 2020b; this study)

#### Notes.

Morphologically, our collection (MFLU 24-0536/MFLUCC 25-0025) resembles the holotype of *H.xylocarpi* (MFLU 18-0545) in having superficial, carbonaceous, uni-perithecial pseudostromata; 8-spored, cylindrical, unitunicate, long-pedicellate asci with a J+, cylindrical apical ring; and dark brown, unicellular, ellipsoid, straight ascospores with both ends often pointed and a straight germ slit on the ventral side along the spore length (Dayarathne et al. 2020b). However, asci (85–115 × 9–17 μm vs. 126–135 × 20–28 μm) and ascospores (13–17 × 7–9 μm vs. 20–26 × 10–14 μm) of our collection (MFLUCC 25-0025) are smaller than the holotype (Dayarathne et al. 2020b). According to multi-gene phylogeny (ITS, *rpb*2, and *β-tub*), our strains (MFLU 24-0536 and MFLUCC 25-0025) cluster with the ex-type strain of *H.xylocarpi* (MFLU 18-0545) with 100% ML bootstrap and 1.00 PP support (Fig. 10). Considering the morpho-molecular evidence, we conclude that our collection is a new host record on decaying submerged wood of Arecaceae sp. in Thailand.

### ﻿ Stilbohypoxylon

Taxon classificationFungiAscomycotaXylariales

Henn., Hedwigia 41: 16 (1902)

4941AF0D-E617-5406-95A8-4A4CCC6B16E9

Index Fungorum: IF5264

Facesoffungi Number: FoF03071

#### Notes.

*Stilbohypoxylon* was established by Hennings (1902) to accommodate *S.moelleri* as the type species. Morphologically, this genus is characterized by black, globose to pulvinate stromata, cylindrical asci with a J+, apical ring; and brown, ellipsoidal ascospores surrounded by a thin mucilaginous sheath and a straight or spiral germ slit. *Stilbohypoxylon* species have geniculosporium-like asexual morphs (Hennings 1902; Rogers and Ju 1997; Petrini 2004; Daranagama et al. 2018). Based on morphology and phylogenetic studies by Daranagama et al. (2018) and Wendt et al. (2018), *Stilbohypoxylon* was accepted in Xylariaceae. *Stilbohypoxylon* species cluster with *Xylaria* species in two subclades, providing evidence that this genus is polyphyletic (Hsieh et al. 2010; Li et al. 2017; Daranagama et al. 2018; Wendt et al. 2018). Hyde et al. (2024) listed 20 species under this genus, while 18 are included in the Index Fungorum (2025).

### ﻿ Stilbohypoxylon chiangraiense

Taxon classificationFungiAscomycotaXylariales

Rathnayaka, K.D. Hyde & Chethana sp. nov.

15643B0D-C30C-577A-B93F-47A23D7CBEBF

Index Fungorum number: IF903884

Facesoffungi Number: FoF17289

Fig. 12

#### Etymology.

The epithet chiangraiense refers to Chiang Rai Province, where the fungus was collected.

#### Holotype.

MFLU 24-0529.

#### Description.

***Saprobic*** on a dead branch of *Saraca* sp. ***Sexual morph*: *Stromata*** superficial, visible as a black conical or globose structure on the host surface, solitary, showing yellow scales on mature stromata, carbonaceous, brittle, fragile. ***Ascomata*** 420–440 × 450–510 μm (x̄ = 427 × 471 μm, n = 10), black, carbonaceous, globose to mammiform, 1 per stroma, covered with remnants of the host tissue, ostioles papillate. ***Peridium*** 10–20 μm wide, thick-walled, composed of several layers of cells of *textura angularis*, dark brown to black. ***Paraphyses*** 2–3 μm wide, filamentous, cylindrical, aseptate, unbranched, longer than asci. ***Asci*** 48–58 × 7–9 μm (x̄ = 53 × 8 μm, n = 10), unitunicate, cylindrical, long pedicellate, apically rounded, with a J+, apical ring (rarely seen). ***Ascospores*** 21–27 × 10–14 μm (x̄ = 24 × 12 μm, n = 30), uniseriate, hyaline when immature, dark brown at maturity, equilateral ellipsoidal to broadly fusoid, unicellular, guttulate, with a spiral germ slit over the whole spore length. ***Asexual morph***: Undetermined.

**Figure 12.**
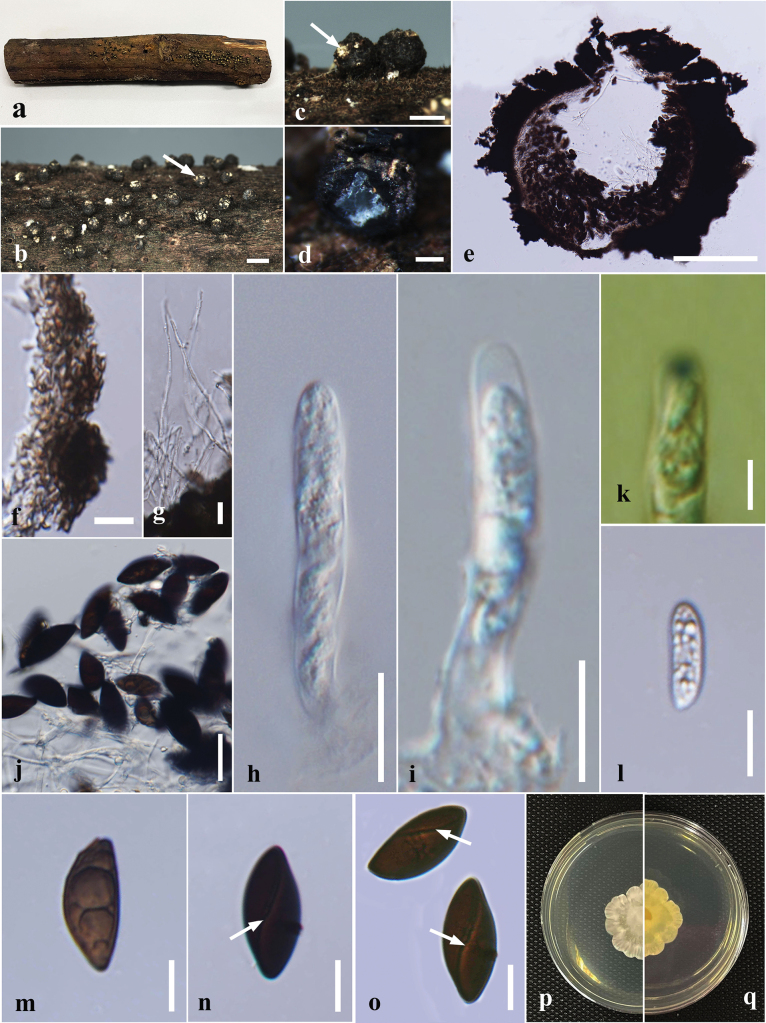
*Stilbohypoxylonchiangraiense* on the dead branch of *Saraca* sp. (MFLU 24-0529, Holotype). **a.** Substrate; **b, c.** Appearance of stromata on the host, showing yellow scales (arrows indicating yellow scales); **d, e.** A horizontal section through a stroma; **f.** Peridium; **g.** Paraphyses; **h, i.** Immature asci; **j.** Apical apparatus stained in blue with Melzer’s reagent; **k–m.** Ascospores; **n, o.** Ascospores with a germ slit (arrows indicating a spiral germ slit); **p, q.** Colony on the PDA (**p** upper, **q** lower). Scale bars: 1 mm (**b**); 500 μm (**c**); 200 μm (**d, e**); 20 μm (**g–j**); 10 μm (**f, k–o**).

#### Culture characters.

Ascospores germinated on the PDA within 24 hours at 25 °C. Germ tubes are produced from both sides of the ascospore. The slow-growing colonies on the PDA reached 1–1.5 cm diam. After five days at 25 °C, circular in shape, cottony, slightly less dense towards the edge, white color in the front view, and pale yellow in the reverse view.

#### Material examined.

Thailand • Chiang Rai, Nang Lae village, on decaying branch of *Saraca* sp. (Fabaceae), 18 March 2024, Achala Rathnayaka, AA13 (MFLU 24-0529, holotype); ex-type living culture, MFLUCC 24-0611.

#### Notes.

Based on the multi-gene phylogeny (ITS, *rpb*2, and *β-tub*), our collections (MFLU 24-0529 and MFLUCC 24-0611) formed a distinct lineage sister to *S.quisquiliarum* (YMJ 172) with 90% ML bootstrap and 0.98 PP support (Fig. 10). *Stilbohypoxylonchiangraiense* shares morphologies similar to the *Stilbohypoxylon* genus by having superficial, solitary, globose stromata, cylindrical asci with a J+, apical ring; and brown, ellipsoidal ascospores with a spiral germ slit. However, asci are very rarely observed in *S.chiangraiense*. *Stilbohypoxylonquisquiliarum* differs from our fungal collection in that its yellow scales turn brown when mature, which are present on the stromata. In addition, our fungal collection has shorter ascospores (21–27 μm) than *S.quisquiliarum* (27.5–28.5 μm) (Petrini 2004). The base pair differences between *S.chiangraiense* (MFLUCC 24-0611) and *S.quisquiliarum* (YMJ 172) are as follows: ITS = 1.9% (11/578), *β-tub* = 11.9% (123/1033). Considering the morpho-molecular data analysis, we established *S.chiangraiense* as a new species in *Stilbohypoxylon*.

### ﻿ Hypoxylon

Taxon classificationFungiAscomycotaXylariales

Bull., Hist. Champ. Fr. (Paris) 1: 168 (1791)

07775400-3971-5843-993C-A1103C1CD36C

Index Fungorum: IF2456

Facesoffungi Number: FoF02980

#### Notes.

Bulliard (1791) introduced *Hypoxylon* to accommodate *H.fragiforme* (basionym: *H.coccineum*) as the type species. The sexual morph of this genus is characterized by ascomata embedded in a colorful, effused, or pulvinate stroma containing secondary metabolites (Ju and Rogers 1996). The ascomata are perithecioid, monostichous, and open separately through umbilicate, rarely slightly papillate ostioles. The asci are 8-spored, unitunicate, cylindrical, stipitate, and provided with a typically amyloid apical apparatus. The ascospores are unicellular, ellipsoid, and brown and have a germ slit on the most convex side of the inequilateral ascospores (Ju and Rogers 1996). The asexual morph is characterized by a nodulisporium-like morph, but other types of conidial states have also been observed, such as sporothrix-like, virgariella-like, and periconiella-like (Ju and Rogers 1996). The evolutionary relationships of hypoxylaceous fungi have been studied using phylogenetic, chemotaxonomic, and morphological data (Kuhnert et al. 2021). Most of the *Hypoxylon* species have been able to produce highly bioactive secondary metabolites, which are released from the stromata (Ju and Rogers 1996; Kuhnert et al. 2014b; Fournier et al. 2016). *Hypoxylon* species have a cosmopolitan distribution and are recorded as saprotrophs that grow on dead wood, endophytes in seed plants, and facultative parasites on diseased hosts (Ju and Rogers 1996; Stadler 2011; Kuhnert et al. 2014a; Daranagama et al. 2018; Helaly et al. 2018; Rogers 2018). Hyde et al. (2024) listed 200 species under this genus, while 466 are included in the Index Fungorum (2025).

##### ﻿Phylogenetic analyses for *Hypoxylaceae*

**Figure 13.**
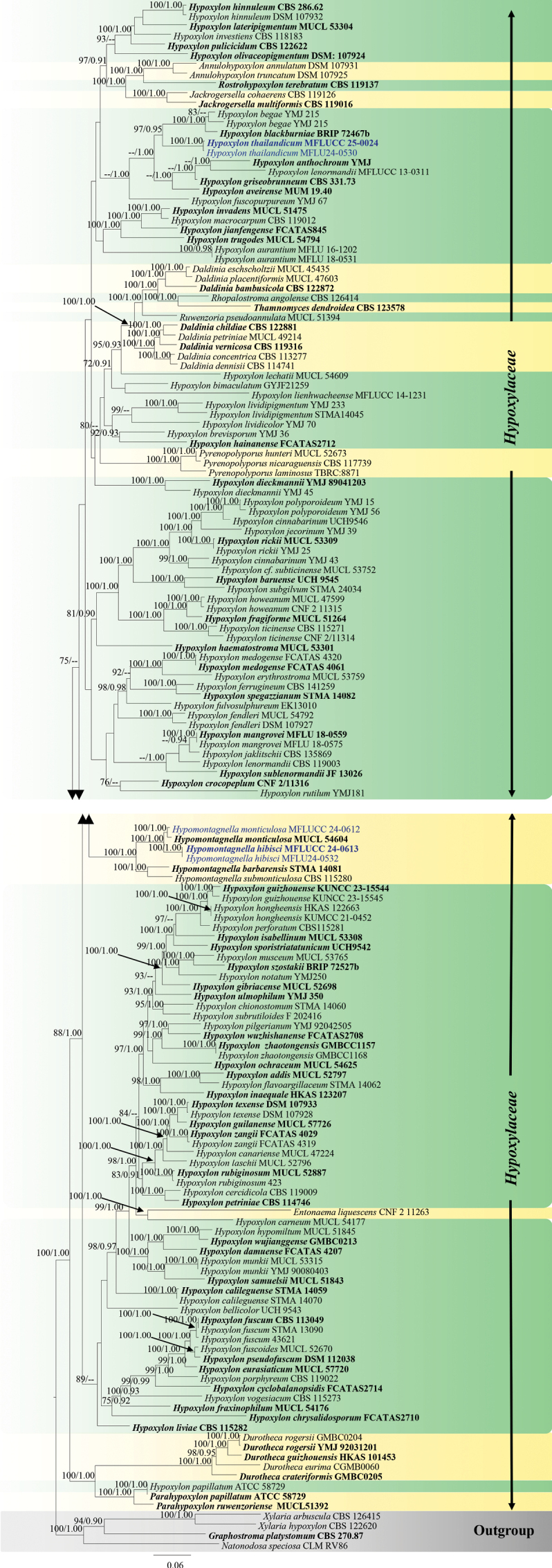
Phylogram generated from ML analysis based on the combined dataset of ITS, LSU, *rpb*2, and *β-tub*. The tree is rooted to *Graphostromaplatystomum* (CBS 270.87), *Natonodosaspeciosa* (CLM RV86), *Xylariaarbuscula* (CBS 126415), and *X.hypoxylon* (CBS 122620). Bootstrap support values for ML ≥ 70% and Bayesian posterior probabilities (PP) ≥ 0.90 are noted at the nodes. Strain numbers are noted after the species names. Strains isolated in this study are represented in blue, and type strains are in bold.

For *Hypoxylon*, 150 taxa were included in the combined data set (ITS, LSU, *rpb*2, and *β-tub*). *Graphostromaplatystomum* (CBS 270.87), *Natonodosaspeciosa* (CLM RV86), *Xylariaarbuscula* (CBS 126415), and *X.hypoxylon* (CBS 122620) were used as the outgroup taxa. After alignment, the dataset comprised 3003 characters, including gaps (ITS = 614 bp, LSU = 822 bp, *rpb*2 = 1017 bp, *β-tub* = 550 bp). Both the ML and BI analyses exhibit a similar tree topology. The best-scoring RAxML tree was obtained (Fig. 13), with a final likelihood value of -75469.623433. The matrix included 1852 distinct alignment patterns, with 29.09% undetermined characters or gaps. The estimated base frequencies were as follows: A = 0.247189, C = 0.251478, G = 0.262907, and T = 0.238426; substitution rates were AC = 1.284950, AG = 4.479967, AT = 1.344479, CG = 1.074839, CT = 6.471802, and GT = 1.0; and the gamma distribution shape parameter α = 0.313445. In the BI analysis, the average standard deviation of the split frequencies was 0.01 after 5,000,000 generations of runs. The phylogenetic tree topology is similar to the study by Karimi et al. (2023). According to the phylogenetic analyses, our strains (MFLU 24-0530, MFLUCC 25-0024, MFLUCC 24-0613, MFLU 24-0532, and MFLUCC 24-0612) cluster within *Hypoxylon* and *Hypomontagnella*.

###### ﻿Taxonomy

### ﻿ Hypoxylon thailandicum

Taxon classificationFungiAscomycotaXylariales

Rathnayaka, K.D. Hyde & Chethana sp. nov.

8CECEFFF-0DB3-539F-ADEE-0E56590A994E

Index Fungorum number: IF903885

Facesoffungi Number: FoF00373

Fig. 14

#### Etymology.

The epithet *thailandicum* refers to Thailand, where the fungus was collected.

#### Holotype.

MFLU 24-0530.

#### Description.

***Saprobic*** on a dead branch of *Bambusavulgaris*. ***Sexual morph*: *Stromata*** 0.3–1 cm long × 0.1–0.5 cm wide, pulvinate, with conspicuous perithecial mounds, gregarious, surface bright orange; orange-red granules immediately beneath the surface and between ascomata, the tissue below the perithecial layer inconspicuous. ***Ascomata*** 200–220 × 195–203 × 180–210 µm (x̄ = 210 × 200 × 190 µm, n = 5), globose, ostiolate. ***Peridium*** 21–27 μm wide, two-layered, outer layer composed of dark brown to brown cells of *textura angularis*, inner layer composed of hyaline cells of *textura angularis*. ***Asci*** 60–105 × 8–11 µm (x̄ = 80 × 9.4 µm, n = 15), 8-spored, unitunicate, cylindrical, pedicellate, with an inamyloid, apical ascal apparatus. ***Ascospores*** 13–18 × 7–10 µm (x̄ = 16 × 8.4 µm, n = 30), uniseriate, slightly overlapping, one-celled, ellipsoid, with narrowly rounded ends, straight, initially light brown, becoming brown to dark brown at maturity, rough surface, guttulate. ***Asexual morph***: Undetermined.

**Figure 14.**
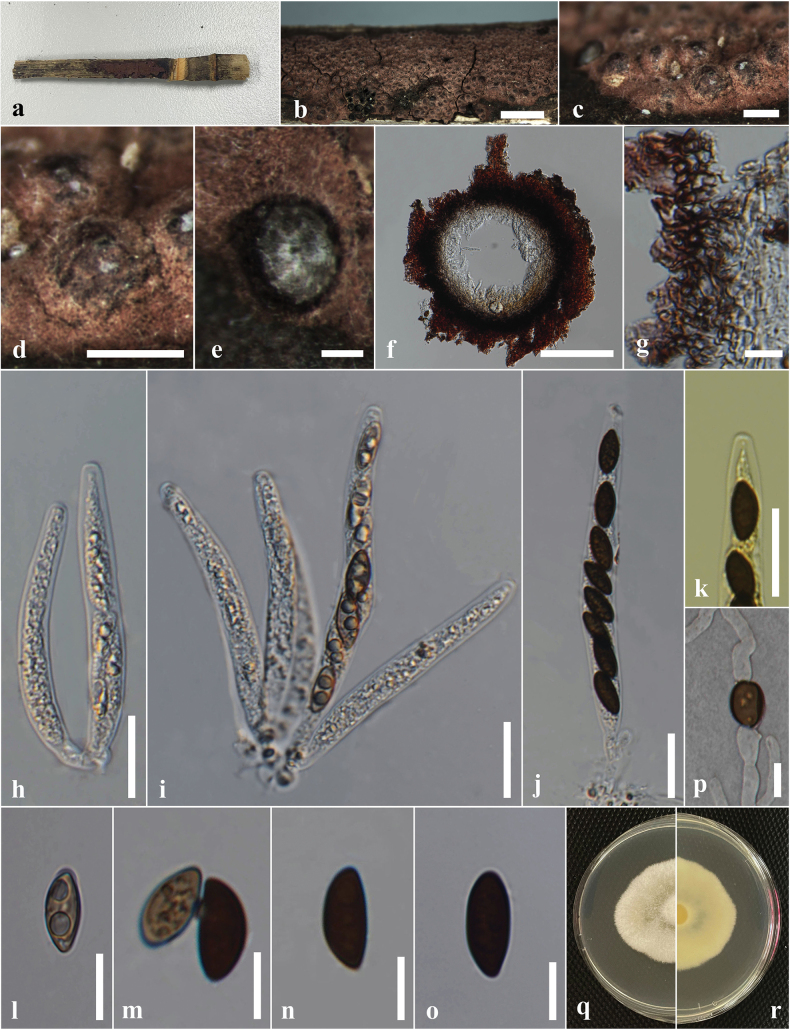
*Hypoxylonthailandicum* on a dead branch of *Bambusavulgaris* (MFLU 24-0530, Holotype). **a.** Substrate; **b–d.** Appearance of ascostromata on the host; **e, f.** A horizontal section through an ascoma; **g.** Peridium; **h–j.** Asci; **k.** Inamyloid apical ascal apparatus stained with Melzer’s reagent; **l–o.** Ascospores; **p.** A germinated ascospore; **q, r.** Colony on the PDA (**q** upper, **r** lower). Scale bars: 1 mm (**b**); 200 μm (**c, d**); 100 μm (**e, f**); 20 μm (**h–k**); 10 μm (**g, l–p**).

#### Culture characters.

Ascospores germinated on the PDA within 24 hours at 25 °C. Germ tubes are produced from both sides of the ascospore. The slow-growing colonies on the PDA reach 1–2 cm diam. after seven days at 25 °C, circular in shape, cottony, slightly less dense towards the edge, white color in the front view, and pale yellow in the reverse view.

#### Material examined.

Thailand • Chiang Rai, Mae Chan District, Mae Chan village, on a dead branch of *Bambusavulgaris* (Poaceae), 18 March 2024, Achala Rathnayaka, AA06 (MFLU 24-0530, holotype); ex-type living culture, MFLUCC 25-0024.

#### Notes.

In multi-gene phylogeny (ITS, LSU, *rpb*2, and *β-tub*), our novel isolates (MFLU 24-0530 and MFLUCC 25-0024) formed a separate lineage sister to *H.begae* (S99 and YMJ 215) and *H.blackburniae* (BRIP 72467b) with 97% ML bootstrap and 0.95 PP support (Fig. 13). Morphologically, our new fungal collection (MFLUCC 25-0024) is similar to *Hypoxylon* by having ascomata embedded in colorful effused or pulvinate stromata (Ju and Rogers 1996). Due to the lack of morphological data for *H.begae* and *H.blackburniae*, we could not compare the morphological characters between these three species. When comparing the ITS base pair differences (without gaps) between *H.thailandicum* (MFLUCC 25-0024) with *H.begae* (YMJ 215) and *H.blackburniae* (BRIP 72467b), there are 9.5% (46/482) and 9.12% (44/482) differences, respectively. For *β-tub*, there is a 10.21% (43/423) base pair difference (without gaps) between *H.thailandicum* (MFLUCC 25-0024) and *H.begae* (YMJ 215). However, due to the lack of sequence availability, we were unable to compare the base pair differences between *H.thailandicum* (MFLUCC 25-0024) and *H.blackburniae* (BRIP 72467b). Additionally, the absence of *rpb*2 sequences prevented a comparison among *H.begae*, *H.blackburniae*, and our collection. Based on the available morphological and phylogenetic evidence, we propose *H.thailandicum* as a new species.

### ﻿ Hypomontagnella

Taxon classificationFungiAscomycotaXylariales

Sir, L. Wendt & C. Lamb., in Lambert, Wendt, Hladki, Stadler & Sir, Mycol. Progr. 18(1–2): 190 (2019)

7C5C3D09-44C5-57EC-874C-F912589A8731

Index Fungorum: IF827251

Facesoffungi Number: FoF06136

#### Notes.

Lambert et al. (2019) introduced *Hypomontagnella* to accommodate *H.monticulosa* as the type species and included several species previously described under *Hypoxylon*. *Hypomontagnella* differs from *Annulohypoxylon* and *Jackrogersella* by smooth perispores or transversally striate ornamentations. Additionally, *Hypomontagnella* species are distinguished from *Hypoxylon* species by woody to carbonaceous stromata that lack colored granules (Lambert et al. 2019). They have papillate ostioles, usually with black annulate discs, without apparent KOH-extractable pigments in mature stromata (Lambert et al. 2019). The cultures of *Hypomontagnella* species produce sporothrolide-type strong antifungal polyketides. Species in this genus have been reported as saprobic or endophytic on plants (Lambert et al. 2019). Six species are listed under *Hypomontagnella* in Hyde et al. (2024) and Index Fungorum (2025).

### ﻿ Hypomontagnella hibisci

Taxon classificationFungiAscomycotaXylariales

Rathnayaka, K.D. Hyde & Chethana sp. nov.

B0C3A28D-4C43-5037-9064-D8FB59AF9633

Index Fungorum number: IF903886

Facesoffungi Number: FoF17290

Fig. 15

#### Etymology.

In reference to the host genus from which the fungus was collected

#### Holotype.

MFLU 24-0532

#### Description.

***Saprobic*** on a dead branch of *Hibiscus* sp. ***Sexual morph*: *Stromata*** effused-pulvinate, with conspicuous to inconspicuous perithecial mounds, surface blackish, carbonaceous tissue immediately beneath the surface and between the perithecial surface and perithecia. ***Ascomata*** 157–300 × 133–218 μm (x̄ = 223 × 178 μm, n = 5), globose to spherical, ostioles higher than the stromatal surface. ***Peridium*** 13–26 μm diam., composed of thin-walled, brown to dark brown cells of *textura angularis*. ***Paraphyses*** 2–3 μm wide, hyaline, filamentous, long, branched, aseptate, arising from the base of ascomata. ***Asci*** 78–100 × 5–6 μm (x̄ = 90 × 5.4 μm, n = 25), the spore-bearing parts 46–53 µm long, 8-spored, cylindrical, with J-, apical ring and a stipe of 28–36 µm long. ***Ascospores*** 7–9 × 3–5 μm (x̄ = 8 × 4 μm, n = 30), unicellular, uni-seriate, slightly overlapping, ellipsoid, with narrowly rounded ends, slightly curved, hyaline to dark brown, smooth to finely roughened, guttulate. ***Asexual morph***: Undetermined.

**Figure 15.**
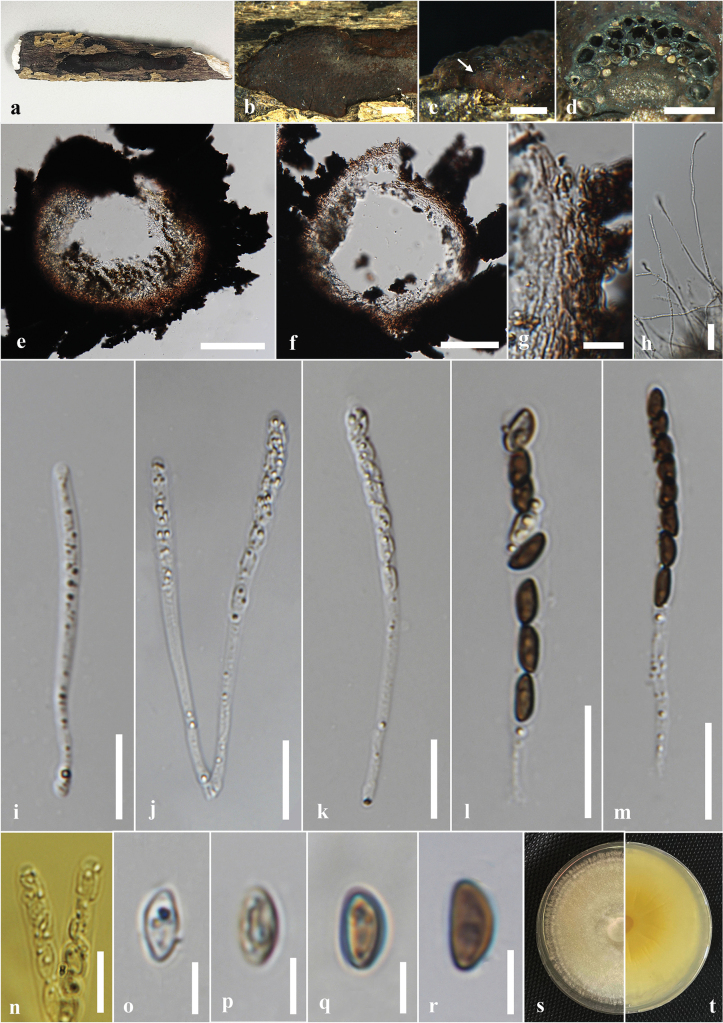
*Hypomontagnellahibisci* on a decaying branch of *Hibiscus* sp. (MFLU 24-0532, Holotype). **a.** Substrate; **b.** Mature stroma on the bark; **c.** Stromatal surface showing papillate and ostiolar discs (indicated by white arrows); **d–f.** Stromata in vertical sections; **g.** Peridium; **h.** Paraphyses; **i–m.** Asci; **n.** Apical apparatus with Melzer’s reagent; **o–r.** Ascospores; **s, t.** Colony on the PDA (**s** upper, **t** lower). Scale bars: 2 mm (**b**); 1 mm (**c**); 200 μm (**d**); 100 μm (**e**); 50 μm (**f**); 10 μm (**g, n**); 20 μm (**h–m**); 5 μm (**o–r**).

#### Culture characteristics.

Ascospores are germinated on the PDA within 24 hours at 25 °C. Germ tubes are produced from one side of the ascospore. Colonies on the PDA reach 1.5–2.5 cm diam. after seven days at 25 °C, circular in shape, flat, cottony, slightly less dense towards the edge, white color in the front view, and pale yellow in the reverse view.

#### Material examined.

Thailand • Chiang Rai, Mae Fah Luang University premises, on a decaying branch of *Hibiscus* sp. (Malvaceae), 08 March 2024, Zaw Lin Tun, AZ01 (MFLU 24-0532, holotype); ex-type culture MFLUCC 24-0613.

#### Notes.

*Hypomontagnellahibisci* (MFLUCC 24-0613) is similar to *Hypomontagnella* in having stromata with conical, papillate ostioles and cylindrical asci with a short pedicel (Lambert et al. 2019). According to the multi-gene phylogenetic analyses, our strains (MFLU 24-0532 and MFLUCC 24-0613) formed a separate lineage sister to *H.monticulosa* (MUCL 54604 and MFLUCC 24-0613) with 100% ML bootstrap and 1.00 PP support (Fig. 13). However, *H.hibisci* has globose to spherical perithecia and asci with J-, apical rings, whereas *H.monticulosa* has spherical to obovoid perithecia and asci with J+ discoid apical rings (Chethana et al. 2021a). Additionally, *H.monticulosa* differs from *H.hibisci* by having ascospores with a straight, spore-length germ slit, which is not observed in *H.hibisci* (Chethana et al. 2021a). With the evidence of unique morphology and distinct phylogeny, we introduce *H.hibisci* as a new species.

### ﻿ Hypomontagnella monticulosa

Taxon classificationFungiAscomycotaXylariales

(Mont.) Sir, L. Wendt & C. Lamb., in Lambert, Wendt, Hladki, Stadler & Sir, Mycol. Progr. 18(1–2): 190 (2019)

6CFB1552-E384-563C-A0BF-D92B6618326B

Index Fungorum: IF827252

Facesoffungi Number: FoF06781

Fig. 16

#### Description.

***Saprobic*** on a dead branch of *Macarangapeltata*. ***Sexual morph*: *Stromata*** effused-pulvinate, with conspicuous to inconspicuous perithecial mounds, surface blackish, woody to carbonaceous tissue immediately beneath the surface and between the perithecial surface and the perithecia. ***Perithecia*** globose to subglobose, ostioles higher than the stromatal surface. ***Paraphyses*** 4–7 μm wide, hyaline, abundant, persistent, unbranched, septate. ***Asci*** 70–95 × 4–6.5 μm (x̄ = 82 × 5.4 μm, n = 20), the spore-bearing parts 40–50 µm long with stipes 36–44 µm long, 8-spored, unitunicate, cylindrical, with J+, discoid apical ring. ***Ascospores*** 6–8 × 3–4 μm (x̄ = 7.2 × 3.4 μm, n = 30), uniseriate, unicellular, ellipsoid-inequilateral, with broadly to less frequently narrowly rounded ends, light brown to brown, smooth. ***Asexual morph***: Undetermined.

**Figure 16.**
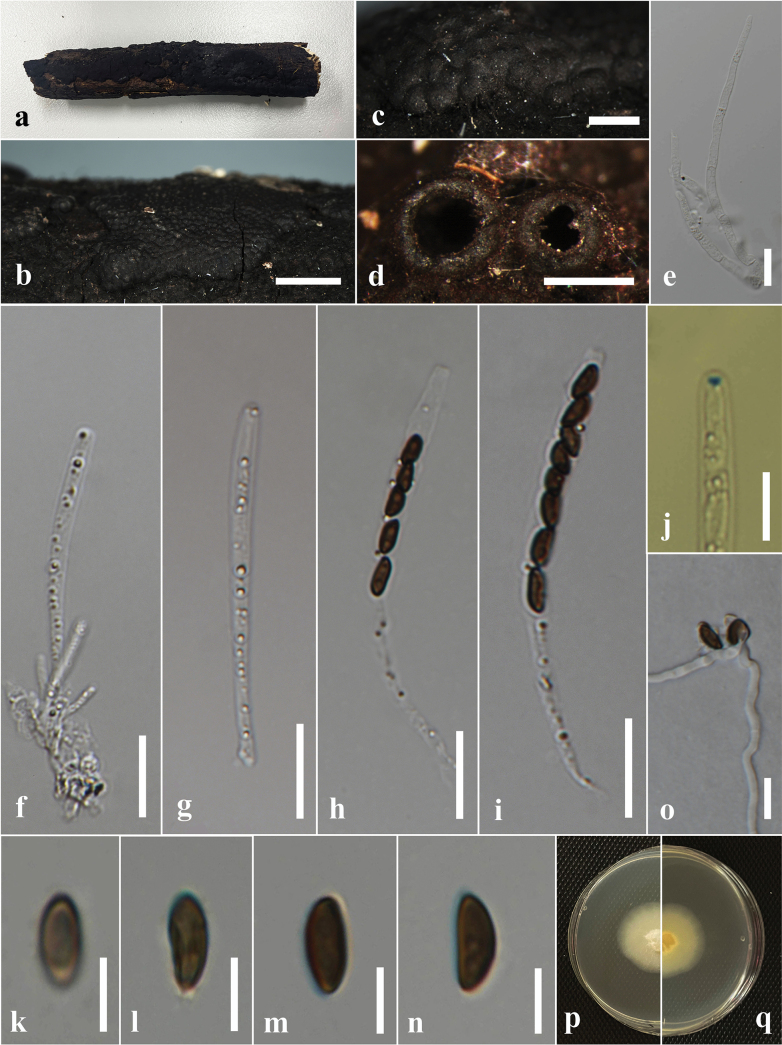
*Hypomontagnellamonticulosa* on a dead branch of *Macarangapeltata* (MFLU 24-0531, a new host record). **a.** Substrate; **b, c.** Appearance of mature stroma on the host; **d.** A horizontal section through ascomata; **e.** Paraphyses; **f–i.** Asci; **j.** Apical apparatus stained blue with Melzer’s reagent; **k–n.** Ascospores; **o.** A germinated ascospore; **p, q.** Colony on the PDA (**p** upper, **q** lower). Scale bars: 2 mm (**b**); 500 μm (**c**); 200 μm (**d**); 20 μm (**e–i**); 10 μm (**j, o**); 5 μm (**k–n**).

#### Culture characteristics.

Ascospores are germinated on the PDA within 24 hours at 25 °C. Germ tubes are produced from one side of the ascospore. The slow-growing colonies on the PDA reach 1.0–1.5 cm diam. after seven days at 25 °C, circular in shape, flat, cottony, slightly less dense towards the edge, white color in the front view, and pale yellow in the reverse view.

#### Material examined.

Thailand • Chiang Rai, Nang Lae village, on decaying branch of *Macarangapeltata* (Euphorbiaceae), 18 March 2024, Achala Rathnayaka, AA10 (MFLU 24-0531); living culture, MFLUCC 24-0612.

#### Known distribution and hosts.

Argentina (*Ficusmaroma*) (Lambert et al. 2019), French Polynesia (dead wood) (Lambert et al. 2019), Indonesia, Malaysia (lichen, Sargassum seaweed) (Zainee et al. 2018), Paraguay (dead wood) (Lambert et al. 2019), Thailand (*Leucaenaleucocephala*, *Macarangapeltata*) (Chethana et al. 2021a, this study), USA (*Cladonialeporina*) (U’Ren et al. 2016).

#### Notes.

Morphologically, our collection (MFLUCC 24-0612) is similar to the ex-type strain of *H.monticulosa* (MUCL 54604), which was collected from a dead branch of *Leucaenaleucocephala* in Thailand (Chethana et al. 2021a). However, asci (70–95 μm vs. 85–110 μm) and ascospores (6–8 μm vs. 7.5–9.3 μm) of our collection (MFLUCC 24-0612) are shorter than the ex-type strain (MUCL 54604) (Chethana et al. 2021a). According to multi-gene phylogeny (ITS, LSU, *rpb*2, and *β-tub*), our strain (MFLUCC 24-0612) clusters with the ex-type strain of *H.monticulosa* (MUCL 54604) with 100% ML bootstrap and 1.00 PP support (Fig. 13). Based on the morpho-molecular evidence, we established the first host record of *H.monticulosa* on *Macarangapeltata* in Thailand.

### ﻿ Diatrypella

Taxon classificationFungiAscomycotaXylariales

(Ces. & De Not.) De Not., Sfer. Ital.: 29 (1863)

A090A059-70E2-5033-8C01-436EEC16FB85

Index Fungorum: IF1505

Facesoffungi Number: FoF11777

#### Notes.

Cesati and De Notaris (1863) introduced *Diatrypella* with *D.verruciformis* as the type species. This genus is characterized by stromata, which are conical to truncate, cushion-like or discoid, and usually delimited by a black zone within host tissues, umbilicate or sulcate ostiolar necks. Asci are cylindrical, polysporous, and long-stalked, and ascospores are hyaline to yellowish. *Diatrypella* species have a libertella-like coelomycete asexual morph (Kirk et al. 2008; Hyde et al. 2020a). There are 74 *Diatrypella* species in Species Fungorum (2024), and only 23 of them have sequence data.

**Phylogenetic analysis for *Diatrypaceae***

**Figure 17.**
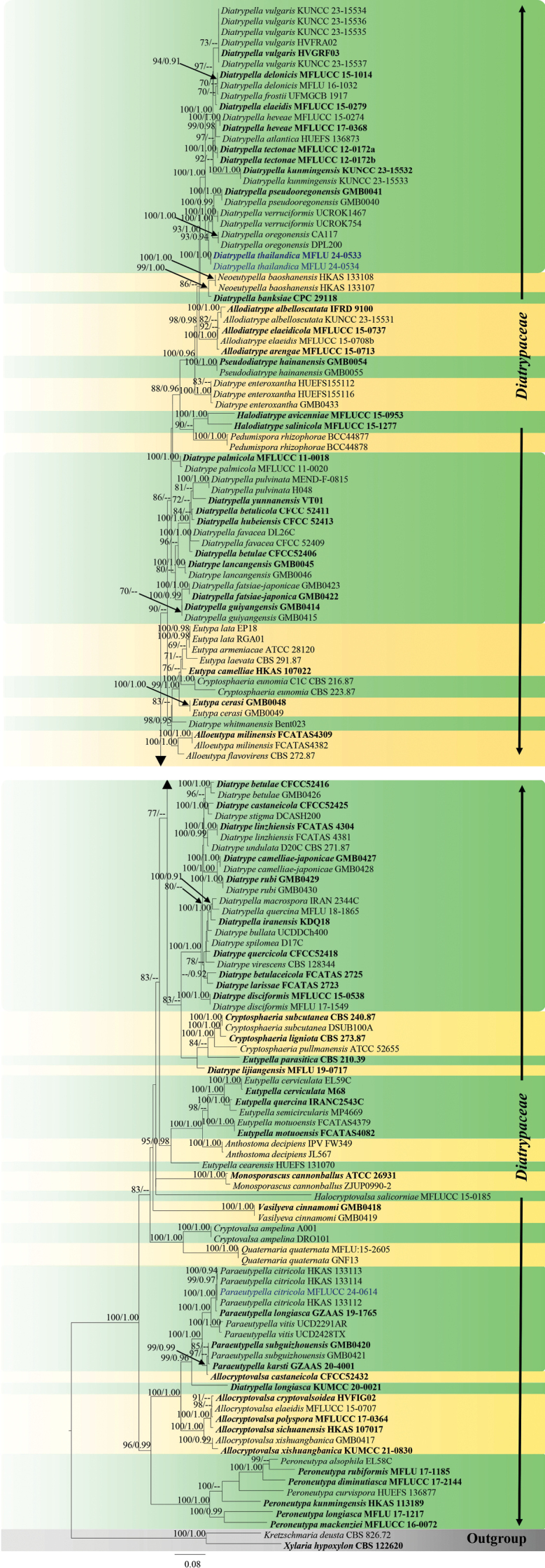
Phylogram generated from ML analysis based on the combined dataset of ITS and *β-tub*. The tree is rooted to *Kretzschmariadeusta* (CBS 826.72) and *Xylariahypoxylon* (CBS 122620). Bootstrap support values for ML ≥ 70% and Bayesian posterior probabilities (PP) ≥ 0.90 are noted at the nodes. Strain numbers are noted after the species names. Strains isolated in this study are presented in blue, and type strains are in bold.

The ITS and *β-tub* combined data set consists of 144 taxa representing strains of Diatrypaceae, including *Kretzschmariadeusta* (CBS 826.72) and *Xylariahypoxylon* (CBS 122620) as the outgroup taxa. The aligned data set comprises 1404 characters, including gaps (ITS = 504 bp, *β-tub* = 897 bp). The topology of the BI tree was similar to that of the ML tree. The best-scoring RAxML tree, with a final likelihood value of -20102.616796, is shown in Fig. 17. The matrix comprises 944 distinct alignment patterns, with 36.45% undetermined characters or gaps. Estimated base frequencies were as follows: A = 0.222804, C = 0.272806, G = 0.233404, T = 0.270985; substitution rates AC = 1.101521, AG = 3.499467, AT = 1.354291, CG = 0.845871, CT = 4.738241, GT = 1.0; gamma distribution shape parameter α = 0.360572. In the BI analysis, the average standard deviation of split frequencies was 0.009 after 9,000,000 generations of runs. The phylogenetic tree topology is similar to the study by Dissanayake et al. (2024). According to the phylogenetic analyses, our strains, MFLU 24-0533 and MFLU 24-0534, formed a separate clade with *Diatrypellaoregonensis* (CA117 and DPL200), *D.pseudooregonensis* (GMB0041 and GMB0040), and *D.verruciformis* (UCROK1467 and UCROK754) with 93% ML bootstrap and 0.94 pp, while MFLUCC 24-0614 clusters with *Paraeutrypellacitricola* (HVGRF01 and HKAS 133111) with 99% ML and 0.97 pp bootstrap support.

##### ﻿Taxonomy

### ﻿ Diatrypella thailandica

Taxon classificationFungiAscomycotaXylariales

Rathnayaka, K.D. Hyde & Chethana sp. nov.

91BF7F2A-2103-54FF-983D-E06CE865E42E

Index Fungorum number: IF903887

Facesoffungi Number: FoF17291

Fig. 18

#### Etymology.

The epithet thailandica refers to Thailand, from where the fungus was collected.

#### Holotype.

MFLU 24-0533.

#### Description.

***Saprobic*** on a dead branch of Fabaceae sp. ***Sexual morph*: *Stromata*** 0.5–1 mm in diam., well-developed, with groups of 10–15 perithecia, solitary to gregarious, erumpent, black, immersed, globose to subglobose or conical shape. ***Endostroma*** white to light yellow. ***Ascomata*** 410–450 μm high × 275–370 μm diam. (x̄ = 434 × 326 μm, n = 10), perithecial, immersed in stromata, 2–4 perithecial arrangement, subglobose, with an individual ostiole. ***Ostiolar canal*** 200–253 μm high, 110–132 μm diam., cylindrical, periphysate, with yellowish pigment around ostioles. ***Peridium*** 10–25 μm wide, composed of 3–7 layers, hyaline to brown, thick-walled cells of *textura angularis*. ***Hamathecium*** 2.4–6 μm wide, comprising dense, hyaline, aseptate, unbranched paraphyses, tapering towards the apex, embedded in a hyaline gelatinous matrix. ***Asci*** 80–150 × 11–23 μm (x̄ = 107 × 16 μm, n = 25), polysporous, unitunicate, clavate to cylindric-clavate, with a J-apical ring and a long pedicel. ***Ascospores*** 6–8 × 1–3 μm (x̄ = 7.5 × 2.3 μm, n = 30), multi-seriate, crowded, initially hyaline, becoming pale yellowish at maturity, oblong to allantoid, aseptate, slightly curved, smooth-walled, mostly with small guttules. ***Asexual morph***: Undetermined.

**Figure 18.**
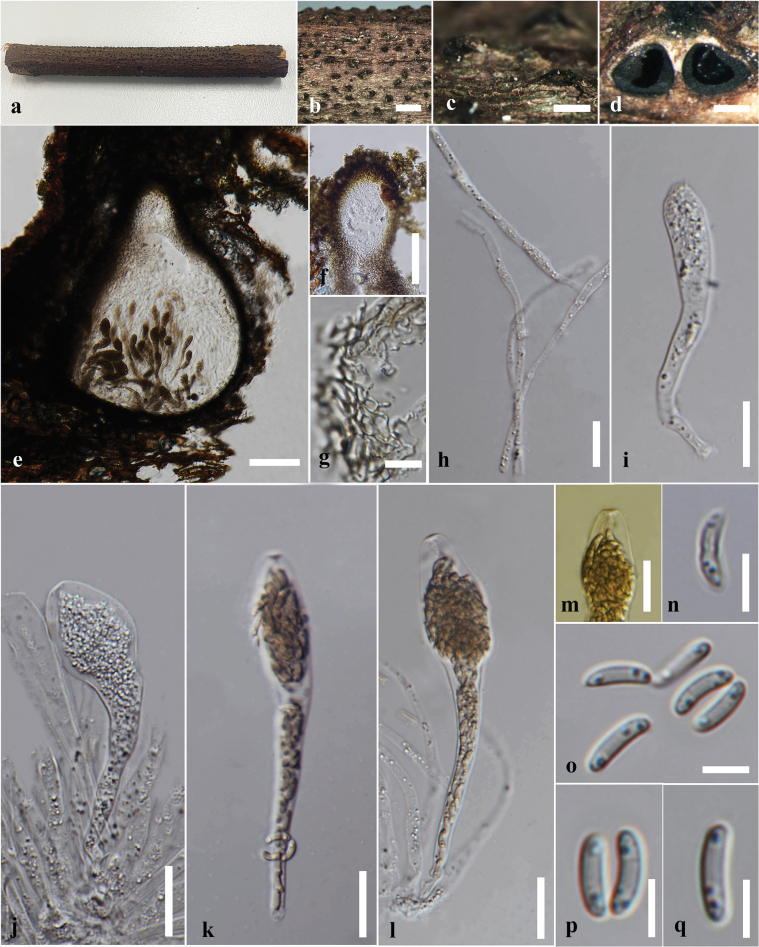
*Diatrypellathailandica* on a dead branch of Fabaceae sp. (MFLU 24-0533, holotype) **a.** Substrate; **b**, **c.** Stromata on the substrate; **d.** Cross-section of a stroma; **e.** Vertical section through stroma showing ostiole and perithecia; **f.** Ostiole; **g.** Peridium; **h.** Paraphyses; **i–l.** Asci; **m.** Apical apparatus in Melzer’s reagent; **n–q.** Ascospores. Scale bars: 2 mm (**b**); 500 μm (**c**); 200 μm (**d**); 100 μm (**e, f**); 10 μm (**g**); 20 μm (**h–m**); 5 μm (**n–q**).

#### Culture characteristics.

Ascospores are germinated on the PDA within 24 hours at 25 °C. Germ tubes are produced from both sides of the ascospore. Colonies on the PDA at 25–28 °C reach 2 cm in 10 days, medium dense, circular to slightly irregular, cottony, white at first, becoming light brownish yellow in the front view, and pale yellow in the reverse view.

#### Material examined.

Thailand • Chiang Rai, Nang Lae village, on a decaying branch of *Morus* sp. (Moraceae), 18 March 2024, Achala Rathnayaka, AA14 (MFLU 24-0533, holotype); *ibid.*, on a dead branch of Fabaceae sp. (Fabaceae), 20 March 2024, Achala Rathnayaka, AA15 (MFLU 24-0534, topotype).

#### Notes.

Our new fungal collection (MFLU 24-0533 and MFLU 24-0534) fits within *Diatrypella* by having conical-shaped stromata, white to light yellow, well-developed endostroma, cylindrical, polysporous, and long-stalked asci, and hyaline to yellowish ascospores (Hyde et al. 2020a). According to the multi-gene phylogenetic analyses (ITS and *β-tub*), our strains (MFLU 24-0533 and MFLU 24-0534) formed a separate clade sister to *D.oregonensis* (CA117, DPL200), *D.pseudooregonensis* (GMB0039, GMB000), and *D.verruciformis* (UCROK1467, UCROK754) with 93% ML bootstrap and 0.94 PP support (Fig. 17). Morphologically, *D.thailandica* differs from *D.oregonensis*, *D.pseudooregonensis*, and *D.verruciformis*, as mentioned in Table 2. *Diatrypellathailandica* has polysporous asci, while *D.oregonensis* and *D.pseudooregonensis* have 8-spored asci. *Diatrypellaverruciformis* differs from our fungal collection by having diamond- or star-shaped ascomata (Table 2). When comparing the ITS and *β-tub* base pairs (without gaps) between *D.thailandica* (MFLU 24-0533) with *D.oregonensis* (CA117), *D.pseudooregonensis* (GMB0039), and *D.verruciformis* (UCROK1467), there are 2.15% (11/512), 3.3% (16/485), and 2.73% (14/512) base pair differences in the ITS and 2.17% (18/828), 2.53% (21/828), and 1.7% (14/828) for *β-tub*, respectively. Based on both morphological and molecular evidence, we introduce *Diatrypellathailandica* (MFLU 24-0533) as a new species in *Diatrypella*.

**Table 2.** Synopsis of morphological characters of sexual morphs between *D.thailandica* and species in the sister clade.

Species	Stromata	Perithecial neck (μm)	Asci	Ascospores	References
*D.thailandica*	groups of 10–15 perithecia, globose to subglobose or conical shape, 0.5–1 mm in diam	200–253 high, 110–132 diam.	Polysporous, 80–150 × 11–23 (x̄ = 107 × 16) μm	6–8 × 1–3 (x̄ = 7.5 × 2.3) μm, L/W = 3.26	This study
*D.oregonensis*	pustules of 1–30 perithecia pulvinate, hemispherical or forming linear stripes, 0.3–0.6 mm diam	–	8-spored, 50–65 (–80) × 6–9.5 μm	(7–)10–12 (14) × 2–2.5	Trouillas et al. (2010)
*D.pseudooregonensis*	groups of 3–16 perithecia, 2 × 1.5 mm	218.5–465 high, 112–257 diam.	8-spored, 95–149 × 6.5–11.5 (av. = 120 × 10.5) μm	11–16 × 1.5–3.5 (x̄ = 14 × 2.5) μm, L/W = 5.6	Long et al. (2021)
*D.verruciformis*	Diamond or star shape, 5–6 × 2–3 mm	Difficulty to recognize	Multispored, 120–140 (170) × 10–14 (16) μm	6–7 (8) × 1.5–2 µm	http://www.taunuspilz.de/coppermine/displayimage.php?pid=9376

### ﻿ Paraeutypella

Taxon classificationFungiAscomycotaXylariales

L.S. Dissan., J.C. Kang, Wijayaw. & K.D. Hyde, Biodiversity Data Journal 9: e63864, 11 (2021)

D95C3B42-DBE2-50A3-BE83-694941A3E59B

Index Fungorum: IF557954

Facesoffungi number: FoF09231

#### Notes.

*Paraeutypella* was introduced by Dissanayake et al. (2021) to accommodate *P.guizhouensis* as the type species, together with *P.citricola* and *P.vitis*, which were previously classified under *Eutypella* sensu lato. The genus is characterized by erumpent, clustered, irregularly shaped, dark brown to black, poorly developed stromata, 8-spored asci; and ascospores that are allantoid, overlapping, and subhyaline (Trouillas et al. 2011; de Almeida et al. 2016; Dissanayake et al. 2021). A coelomycetous asexual morph has been recorded in this genus, which was characterized by black, subconic, multi-loculate, largely prosenchymatous conidiomata with yellowish conidial masses. Conidia are hyaline, single-celled, slightly curved, and guttulate (Glawe and Jacobs 1987). There are six species listed in the Index Fungorum (2025).

### ﻿ Paraeutypella citricola

Taxon classificationFungiAscomycotaXylariales

(Speg.) L.S. Dissan., Wijayaw., J.C. Kang & K.D. Hyde, Biodiversity Data Journal 9: e63864, 14 (2021)

2D28D00C-1D4A-5D13-8A8E-923EDBB3BB27

Index Fungorum: IF557954

Facesoffungi Number: FoF09150

Fig. 19

Eutypella citricola Syd. & P. Syd., Hedwigia 49: 80 (1909), nom. illegit., Art. 53.1. Basionym.

#### Description.

*Saprobic* on a dead branch of *Swieteniamacrophylla*. ***Sexual morph*: *Stromata*** immersed in the bark of dead branches, erumpent, aggregated, circular to irregular in shape, superficial, carbonaceous. ***Endostroma*** white to light yellow. ***Ostiole*** opening separately, papillate or apapillate, central. ***Ascomata*** 840–880 μm high × 430–455 μm diam. (x̄ = 867 × 446 µm, n = 10), perithecial, with groups of 5–10 perithecia arranged in a valsoid configuration, black, subglobose, clustered, immersed in ascostroma with an ostiolar neck. ***Necks*** 220–265 μm long (x̄ = 248 µm, n = 10), papillate, central ostiolar canal filled with paraphyses. ***Peridium*** 25–48 μm wide, composed of two layers of *textura angularis* to *textura prismatica*; inner layer cells hyaline, outer layer cells brown to dark brown. ***Hamathecium*** 3–5 μm wide (x̄ = 4 µm, n = 15) comprises hyaline, long, narrow, unbranched, aseptate, guttulate cells, paraphyses arising from the base of perithecia. ***Asci*** 56–94 × 5–7 μm (x̄ = 67 × 6.4 μm, n = 20), 8-spored, unitunicate, thin-walled, clavate to cylindrical clavate, long pedicellate (35–55 μm), J- apical ring. ***Ascospores*** 7–9 × 2–3 μm (x̄ = 8 × 2.4 μm, n = 40), overlapping 2–3 seriate, allantoid, hyaline to light brown, smooth, aseptate, usually with small guttules. ***Asexual morph***: Undetermined.

**Figure 19.**
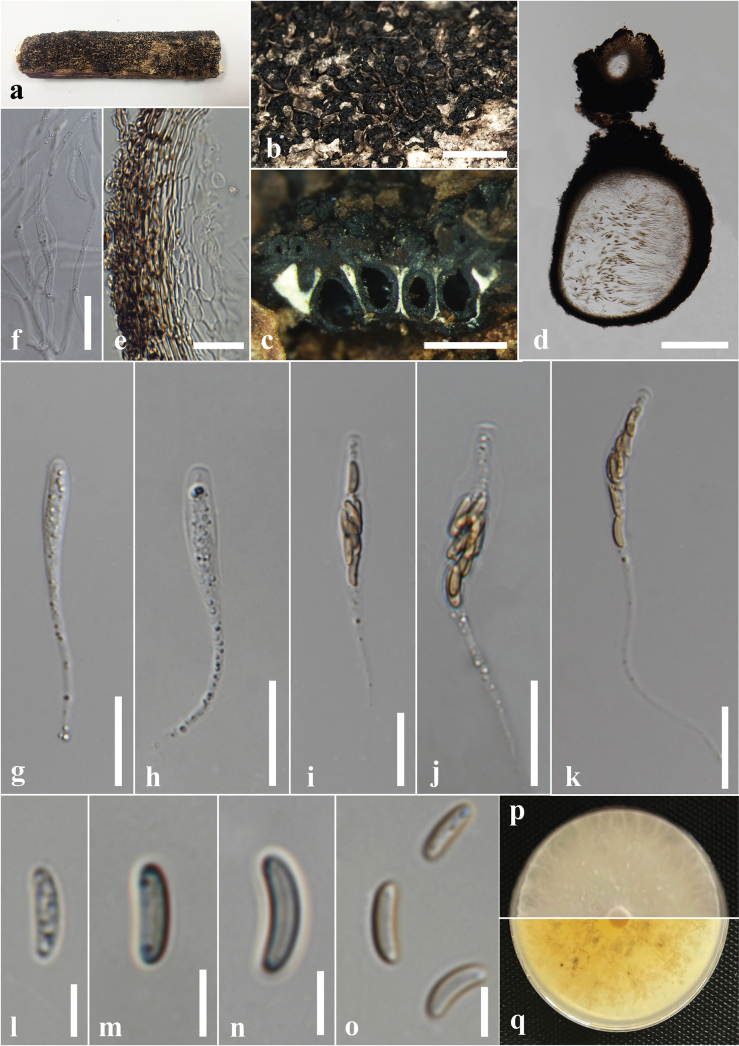
*Paraeutypellacitricola* on a dead branch of *Swieteniamacrophylla* (MFLU 24-0535, a new host record). **a.** Substrate; **b.** Stromata on the substrate; **c.** A cross-section of a stroma; **d.** A vertical section through the stroma shows ostioles and perithecia; **e.** Peridium; **f.** Paraphyses; **g–k.** Asci; **l–o.** Ascospores; **p, q.** Colony on the PDA (**p** upper, **q** lower). Scale bars: 5 mm (**b**); 1 mm (**c**); 200 μm (**d**); 20 μm (**e–k**); 5 μm (**l–o**).

#### Culture characteristics.

Ascospores are germinated on the PDA within 24 hours at 25 °C. Germ tubes are produced from one side of the ascospore. Colonies on the PDA at 25–28 °C reaching 3–5 cm in five days, medium dense, circular to slightly irregular, cottony, white color in the front view, and pale yellow in the reverse view.

#### Material examined.

Thailand • Chiang Rai, Nang Lae village, on a decaying branch of *Swieteniamacrophylla* (Meliaceae), 08 April 2024, Achala Rathnayaka, AA16 (MFLU 24-0535); living culture, MFLUCC 24-0614.

#### Known distribution.

Wide host range and widely distributed in temperate, tropical, and subtropical regions (Senwanna et al. 2021).

#### Notes.

Based on the phylogenetic analyses, our collection (MFLUCC 24-0614) clustered with other strains of *P.citricola* (HKAS 13311 and HVGRF01) with 100% ML bootstrap and 1.00 PP support (Fig. 17). Morphologically, our collection is similar to the holotype of *P.citricola* (HMAS 290660), which was collected from the dead twigs of *Acerpalmatum* in China (Dissanayake et al. 2021). Both specimens share similar morphological characteristics, including immersed, erumpent, aggregated, superficial, carbonaceous stromata; black, subglobose, clustered ascomata immersed in the ascostroma with an ostiolar neck; 8-spored, unitunicate, clavate to cylindrical-clavate, long pedicellate asci with a J- apical ring; and allantoid, hyaline to light brown, aseptate ascospores, usually with small guttules (Dissanayake et al. 2021). However, our collection has a shorter neck (220–265 µm vs. 360–390 µm) and longer asci (56–94 µm vs. 70–75 µm) than the holotype (Dissanayake et al. 2021). Based on the morpho-molecular evidence, *Paraeutypellacitricola* has been recorded from Thailand on various woody plants, including *Heveabrasiliensis* (Senwanna et al. 2021), *Magnolia* sp. (de Silva et al. 2022), and *Microcospaniculata* (Afshari et al. 2023). We identified our collection as a new host record of *Paraeutypellacitricola* from *Swieteniamacrophylla* from Thailand.

##### ﻿Preliminary screening for antibacterial activity

**Figure 20.**
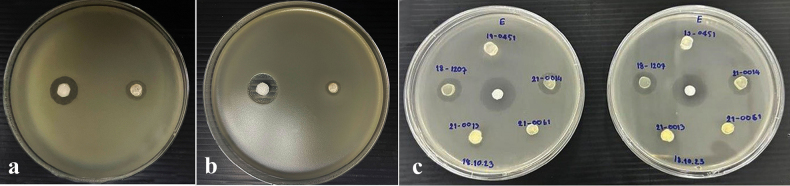
Preliminary screening of antimicrobial activity using the agar plug diffusion method against *Bacillussubtilis* (**a, b**) and *E.coli* (**c, d**). **a.***Annulohypoxylonchiangraiense* (MFLUCC 24-0606); **b.***Hypoxylonthailandicum* (MFLUCC 25-0024); **c.***Daldiniaeschscholtzii* (MFLUCC 18-1207), and *Xylariachrysanthum* (MFLUCC 21-0014). Positive control (ampicillin discs): Left side of the plate in a and b; middle of the petri plate in c.

In the present study, we conducted a preliminary screening to assess the antibacterial activity of selected fungal species against *Bacillussubtilis* (TISTR 1248) and *Escherichiacoli* (TISTR 527). Two newly introduced species from Hypoxylaceae and Xylariaceae, *Annulohypoxylonchiangraiense* (MFLUCC 24-0606) and *Hypoxylonthailandicum* (MFLUCC 25-0024), showed antibacterial activity against *Bacillussubtilis*, each producing a 2 mm zone of inhibition, indicating partial inhibition compared to the positive control. Additionally, other Xylariales species, including *Hypoxylon* sp. (MFLUCC 18-1207), *Daldiniaeschscholtzii* (MFLUCC 18-1207), and *Xylariachrysanthum* (MFLUCC 21-0014), demonstrated antibacterial activity against *E.coli*, each producing a 3 mm zone of inhibition, respectively, compared to the positive control (Fig. 20).

##### ﻿Metabolite profiling of selected fungal extracts by HPLC coupled to LC-QTOF-MS analyses

**Figure 21.**
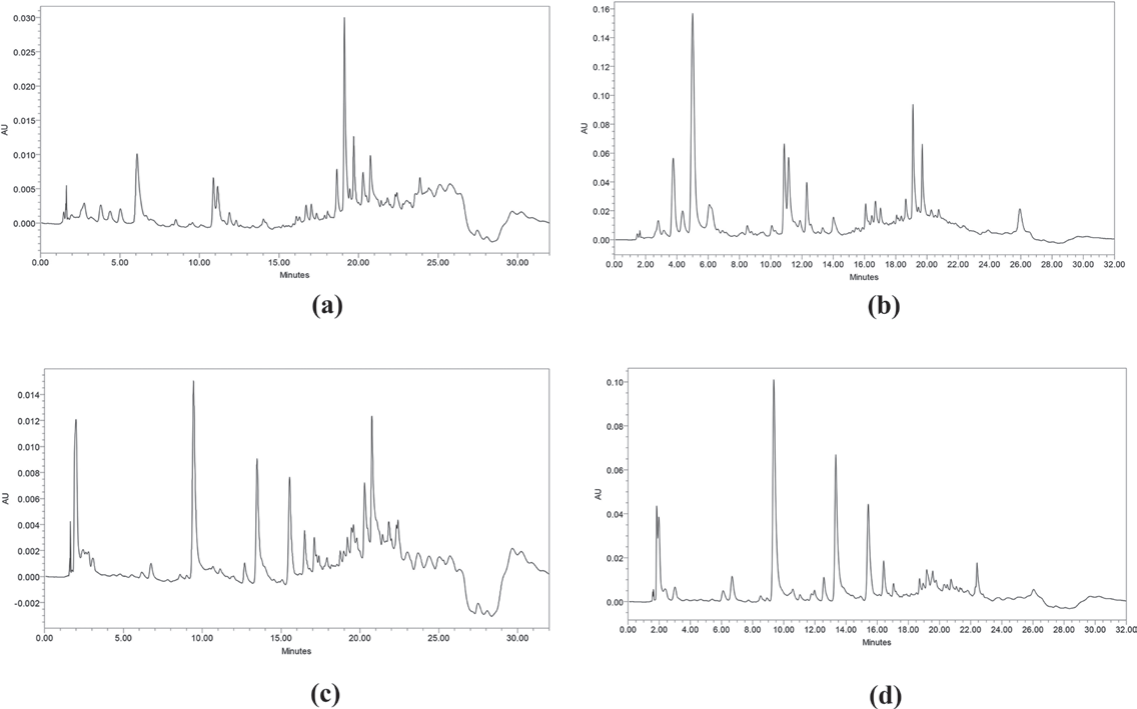
HPLC chromatograms of the culture extracts of *Annulohypoxylonchiangraiense* (MFLUCC 24-0606) **a.** Mycelium in MEB media; **b.** Supernatant in MEB media. *Hypoxylonthailandicum* (MFLUCC 25-0024); **c.** Mycelium in YMB media; **d.** Supernatant in YMB media.

**Figure 22.**
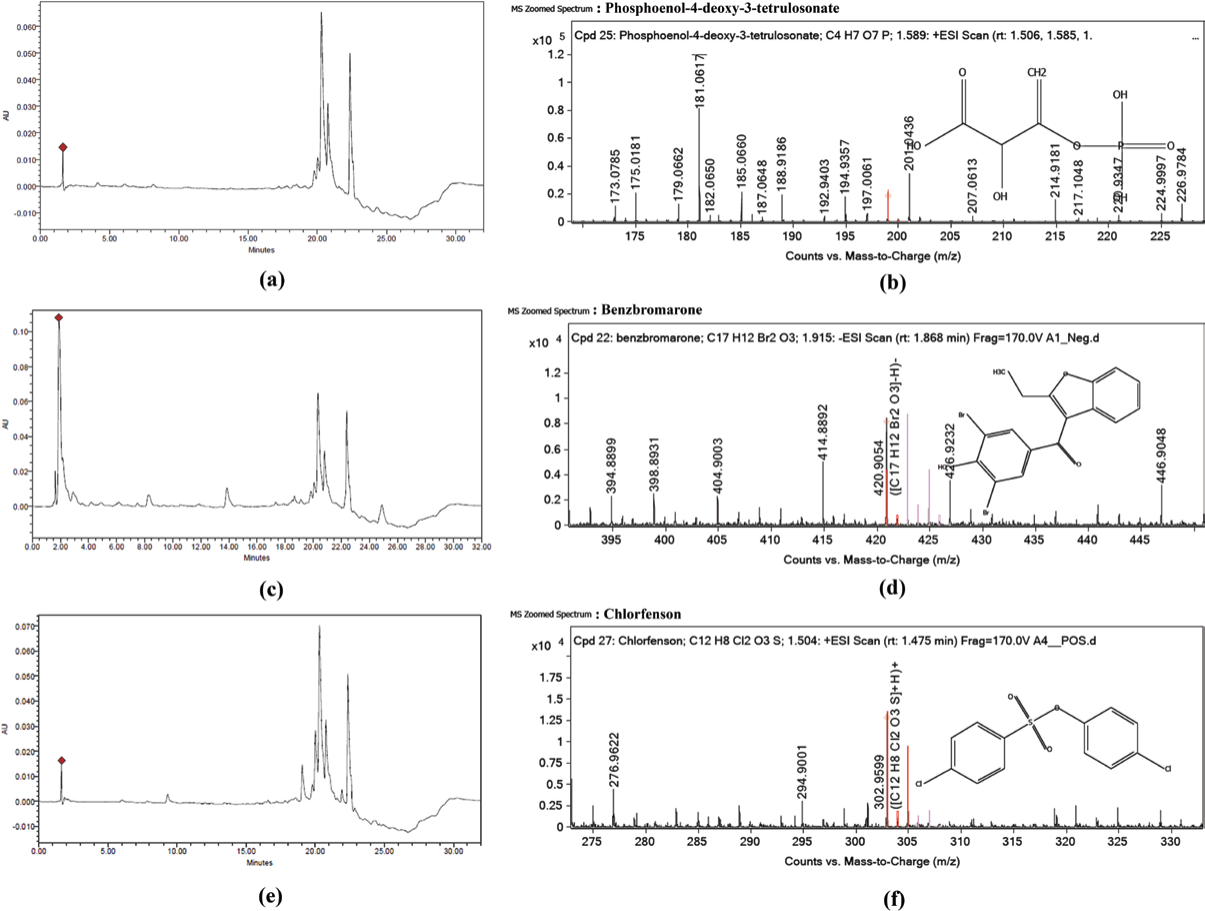
HPLC chromatograms of the culture extracts of *Xylariachrysanthum* (MFLUCC 21-0014) **a.** in MEB media; **b.** Phosphoenol-4-deoxy-3-tetrulosonate; **c.** in YMB media; **d.** Benzbromarone; **e.***Daldiniaeschscholtzii* (MFLUCC 18-1207) in MEB media; **f.** Chlorfenson. Peaks corresponding to metabolites are indicated in the HPLC profiles.

The crude extracts of *Annulohypoxylonchiangraiense*, *Hypoxylonthailandicum*, *Daldiniaeschscholtzii*, and *Xylariachrysanthum* were weighed at 0.01 g, 0.02 g, 0.08 g, and 0.02 g, respectively. An untargeted screening approach initially detected secondary metabolites at wavelengths of 256 and 425 nm. Secondary metabolites have been spotted by the information from spectra and molecular weight, matched with reference compounds from the online databases. Some characteristic HPLC chromatograms are shown in Figs 21, 22.

As Fig. 21 depicts, the HPLC analysis revealed distinct differences in the metabolite profiles of *Annulohypoxylonchiangraiense* and *Hypoxylonthailandicum* when cultivated in different media. For *Annulohypoxylonchiangraiense*, the metabolic output was highly dependent on the growth medium. The mycological extract derived from malt extract broth (MEB) exhibited a complex metabolite profile with numerous peaks, indicating the production of a diverse array of secondary metabolites. This suggests that MEB provides favorable conditions for the production of a broad range of secondary metabolites. Similar findings have been reported in previous studies, where MEB was shown to enhance the production of secondary metabolites in various fungi, including *Hypoxylon* and *Xylaria* species, due to its rich composition of carbohydrates, amino acids, and vitamins (Kuhnert et al. 2015). In contrast, potato dextrose broth (PDB) resulted in significantly fewer peaks, suggesting a reduced metabolic diversity under these conditions. This could be attributed to the simpler composition of PDB, which may not provide the necessary nutrients or environmental conditions to induce the production of a broad spectrum of secondary metabolites.

Similarly, yeast malt broth (YMB) also showed fewer peaks compared to MEB, suggesting that PDB and YMB may not be as conducive for secondary metabolite production in *Annulohypoxylonchiangraiense*. However, it is noteworthy that *Hypoxylonthailandicum* exhibited the highest number of well-defined peaks in YMB, indicating that this medium was optimal for the secondary metabolite production of this species. Similar results have been shown in other studies where they report unique metabolites in YMB, such as hypoxylonols, which are not as prominently produced in other media (Kuhnert et al. 2017). These findings highlight the influence of nutritional composition on secondary metabolite production in different fungal taxa. The variation in peak intensities and numbers across different media indicates that metabolite expression is medium-dependent and species-specific, and hence, must be carefully considered in natural product discovery and metabolic studies.

The mass spectrum obtained from the analysis suggested an identification of many compounds. Based on the HPLC profile of the *Annulohypoxylonchiangraiense* (MFLUCC 24-0606) isolate, the dominant compounds in the mycelium grown in MEB medium are 5-amino-2-(p-toluidino)benzenesulphonic acid (C_13_H_14_N_2_O_3_S), sulfamethoxazole sodium (C_10_H_10_N_3_NaO_3_S), N,N-dibutyl-3-methyl-5-(4,4,5,5-tetramethyl-1,3,2-dioxaborolan-2-yl)aniline (C_21_H_36_BNO_2_), and 2-(benzyloxy)-5-(4,4,5,5-tetramethyl-1,3,2-dioxaborolan-2-yl)pyridine (C_18_H_22_BNO_3_), detected at retention times of 6.0, 11.1, 19.0, and 20.0 minutes, respectively (Fig. 21a). Among these compounds, 5-amino-2-(p-toluidino)benzenesulphonic acid is an aromatic sulfonic acid primarily used in the dye and pigment industries (National Center for Biotechnology Information 2025a). Sulfamethoxazole sodium is the sodium salt form of sulfamethoxazole, which is used as a broad-spectrum sulfonamide antibiotic. Sulfamethoxazole sodium is mainly used in treating bacterial infections, including urinary tract infections (UTIs), respiratory infections, gastrointestinal infections, and skin infections (Kemnic and Coleman 2022).

The dominant compounds of the *Annulohypoxylonchiangraiense* (MFLUCC 24-0606) in the supernatant in MEB medium are Pramipexole (C_10_H_17_N_3_S), 5-Ethyl-1-ethoxymethyl-6-(3,5-dimethylphenylthio)uracil, 10-Deoxymethymycin (C_25_H_43_NO_6_), and Spisulosine (C_18_H_39_NO), detected at retention times of 5.0, 11.0, 12.5, and 19.75, respectively (Fig. 21b). Pramipexole is an orally active aminothiazole dopamine and is used to treat Parkinson’s disease (Bennett and Piercey 1999). 5-Ethyl-1-ethoxymethyl-6-(3,5-dimethylphenylthio)-2-thiouracil has antiviral, anticancer, and antimicrobial properties and is active against the majority of viruses (Balzarini et al. 1995). 10-Deoxymethymycin is a glycoside, a macrolide antibiotic that is active against gram-positive bacteria (National Center for Biotechnology Information 2025b). Spisulosine is a bioactive sphingoid and has been used as an antineoplastic agent (National Center for Biotechnology Information 2025c).

According to the HPLC profile in the *Hypoxylonthailandicum* (MFLUCC 25-0024) isolate, the dominant compounds in the mycelium in YMB medium are 2-Naphthalenepropanol, 6-methoxy-a-methyl-, hydrogen sulfate (C_15_H_18_O_5_S), N-({[Dimethoxy(methyl)silyl]oxy}methyl) aniline (C_10_H_17_NO_3_Si), Phenethyl rutinoside (C_20_H_30_O_10_), and Ethyl vanillin (C_9_H_10_O_3_), detected at retention times of 9.54, 13.55, 20.25, and 20.75 minutes, respectively (Fig. 21c). 2-Naphthalenepropanol, 6-methoxy-α-methyl-, hydrogen sulfate is a sulfate ester of a naphthalene-derived compound, potentially related to antibacterial naphthol derivatives (Roman et al. 2016). N-({[Dimethoxy(methyl)silyl]oxy}methyl)aniline is utilized in various industries due to its unique properties. This compound can be used as a versatile silane coupling agent, enhancing the adhesion, wetting, and durability of different materials. Therefore, N-({[Dimethoxy(methyl)silyl]oxy}methyl)aniline plays a crucial role in the production of adhesives, sealants, coatings, and composites (Inno Specialty Chemicals 2017). Phenethyl rutinoside is a glycoside that exhibits antioxidant activity (Wang et al. 2004). Ethyl vanillin belongs to the benzaldehydes and is used as an antioxidant and flavoring agent. This compound is widely used in the food industry as a food additive and spice in foods, beverages, cosmetics, and drugs (National Center for Biotechnology Information 2025d).

The dominant compounds of the *Hypoxylonthailandicum* (MFLUCC 25-0024) in the supernatant in YMB medium are 1,4-dimethyl-7-ethylazulene (C_14_H_16_), 3-{[(Benzyloxy)carbonyl]amino}-N-(tertbutoxycarbonyl)-L-alanine—N-cyclohexylcyclo hexanamine (1/1) (C_28_H_45_N_3_O_6_) and ethyl 2-phenyl-3-furancarboxylate (C_13_H_12_O_3_), detected at retention times of 9.54, 13.55, and 15.59 minutes, respectively (Fig. 21d). Ethyl 2-phenyl-3-furancarboxylate is an ester derivative of furan and can be used in pharmaceuticals, fragrances, and organic synthesis (Yannai 2004).

For *Xylariachrysanthum* (MFLUCC 21-0014), most of the bioactive compounds have been reported in MEB, followed by YMB. In MEB media, *Xylariachrysanthum* (MFLUCC 21-0014) showed phosphoenol-4-deoxy-3-tetrulosonate (C_4_H_7_O_7_P) at 1.5 min retention time. Phosphoenol-4-deoxy-3-tetrulosonate plays a key role as an intermediate compound in the biosynthesis of 3-deoxy-D-manno-octulosonic acid (KDO), an important molecule in bacterial biochemistry (Fig. 22a, b). Additionally, benzbromarone (C_17_H_12_Br_2_O_3_) has also been reported to be extracted from *Xylariachrysanthum* (MFLUCC 21-0014) in MEB medium, exhibiting antibacterial properties against Gram-positive pathogens, such as *Enterococcusfaecalis*, *Staphylococcusaureus*, *S.epidermidis*, and *Streptococcusagalactiae* (Meng et al. 2024).

According to the HPLC profile in the *Xylariachrysanthum* (MFLUCC 21-0014) isolate, the dominant compounds in the YMB medium are benzbromarone (C_17_H_12_Br_2_O_3_) (Fig. 22c, d). Benzbromarone, a benzofuran derivative, exhibits potential antibacterial activity against Gram-positive pathogens. Benzbromarone is used as a uricosuric drug that has been used in the treatment of gout and hyperuricemia (high levels of uric acid in the blood) (Heel et al. 1977; Meng et al. 2024). Additionally, monensin (C_32_H_58_O_13_), a polyether antibiotic widely used in veterinary medicine for its efficacy against certain Gram-positive bacteria and protozoa, was also reported in the *Xylariachrysanthum* (MFLUCC 21-0014) isolate in YMB medium (Łowicki and Huczyński 2013).

The HPLC analysis of *Daldiniaeschscholtzii* (MFLUCC 18-1207) cultured in MEB media revealed a peak corresponding to chlorfenson (C_12_H_8_C_l2_O_3_S), with a retention time of 1.5 minutes. Chlorfenson is an organophosphorus compound, mainly used as a pesticide and acaricide (National Center for Biotechnology Information 2025e). (Fig. 22e, f). Additionally, in the *Daldiniaeschscholtzii* (MFLUCC 23–0263) isolate, the most abundant compound in the MEB medium is 13-methoxy-heneicosanoic acid (C_22_H_44_O_3_), followed by linoleic acid (C_18_H_32_O_2_). Both 13-methoxy-heneicosanoic acid and linoleic acid exhibited antimicrobial properties and have potential applications in research, cosmetics, pharmaceuticals, nutraceuticals, and industrial products (Kusumah et al. 2020). Furthermore, 3-methoxymandelic acid-4-O-sulfate (C_9_H_10_O_8_S) and formoterol (C_19_H_24_N_2_O_4_) have been identified in *Daldiniaeschscholtzii* on MEA, demonstrating medicinal properties.

## ﻿Discussion

Thailand is part of the Indo-Malayan hub of biodiversity and is geographically located in the core of the Greater Mekong Subregion (Chaiwan et al. 2021). Thailand is well known to have tropical seasonal forests with rich and diverse plant communities. Therefore, a huge diversity of fungi can be found in Thailand (Tanaka et al. 2008; Vasilyeva et al. 2012; Hyde et al. 2018).

The present study includes the taxonomy of fungi in the families of Xylariales. Based on morphological aspects and phylogenetic analyses, we provided the taxonomic details of five novel species and ten new host/geographical records within Diatrypaceae, Hypoxylaceae, and Xylariaceae. Taxa were collected from February 2023 to July 2024 from forest areas with a high variety of trees and well-grown understory vegetation. These saprobic specimens were collected from different host families. We introduced two novel species in Hypoxylaceae: *Annulohypoxylonchiangraiense* from *Tamarindusindica* and *Hypomontagnellahibisci* from *Hibiscus* sp.; two novel species in Xylariaceae: *Hypoxylonthailandicum* from *Bambusavulgaris* and *Stilbohypoxylonchiangraiense* from *Saraca* sp.; and one novel species in Diatrypaceae: *Diatrypellathailandica* from Fabaceae sp. from Thailand. These five taxonomic novelties fulfilled the basic criteria for establishing new species, including distinct morphologies and multiple loci for the phylogenetic analyses, as described by Chethana et al. (2021b). Six new host records from Hypoxylaceae were recorded from Thailand: *A.bahnphadengense* from *Berryacordifolia*, *A.crowfoothodgkiniae* from *Swieteniamacrophylla*, *A.purpureonitens* from *Sterculiatragacantha*, *A.spougei* from *Antidesmamadagascariense*, *A.violaceopigmentum* from *Syzygiumpolyanthum*, and *Hypomontagnellamonticulosa* from *Macarangapeltata*. Our study also records *A.crowfoothodgkiniae* from Thailand for the first time. *Astrocystisbambusae* from *Bambusavulgaris* and *Haloroselliniaxylocarpi* from Arecaceae sp. were recorded as new host records from Xylariaceae in Thailand. In addition, we include *Paraeutypellacitricola* from *Swieteniamacrophylla* in Diatrypaceae as a new host record from Thailand.

In this study, we examined the antibacterial activity of the newly introduced species, *Annulohypoxylonchiangraiense* (MFLUCC 24-0606) and *Hypoxylonthailandicum* (MFLUCC 25-0024), using a preliminary screening test. Both species showed partial inhibition of the growth of *Bacillussubtilis*. Additionally, some existing Xylariales species, *Daldiniaeschscholtzii* (MFLUCC 18-1207) and *Xylariachrysanthum* (MFLUCC 21-0014), also exhibited slight inhibition zones against the bacterial pathogens. Many studies have been conducted on the antimicrobial properties of *Annulohypoxylon*, *Daldinia*, *Hypoxylon*, and *Xylaria* species (Quang et al. 2005; Yuyama et al. 2017; Pourmoghaddam et al. 2020; Gauchan et al. 2021; Segundo et al. 2022; Brooks et al. 2024; Cedeño-Sanchez et al. 2024; Chen et al. 2024; Yu et al. 2024).

The current study identified some of the secondary metabolites extracted from Xylariales isolates exhibiting biological properties, including antimicrobial, antibiotic, antiviral, and anticancer properties. All four tested fungal species exhibit antibacterial metabolites, including sulfamethoxazole sodium from *Annulohypoxylonchiangraiense*, 2-naphthalenepropanol, 6-methoxy-α-methyl-, hydrogen sulfate from *Hypoxylonthailandicum*, benzbromarone from *Xylariachrysanthum*, and linoleic acid from *Daldiniaeschscholtzii*. Therefore, this study will generate the initial data necessary for large-scale metabolite extractions for future applications.

The genus *Annulohypoxylon* can produce secondary metabolites with cytotoxic, antibacterial, and antioxidant properties (Maciel et al. 2018). Here, we recorded sulfamethoxazole sodium as an antibacterial compound from *Annulohypoxylonchiangraiense*. The genus *Hypoxylon* has been identified as the main producer of potential bioactive metabolites in Hypoxylaceae (Stadler et al. 2006). *Hypoxyloninvadens* produces flaviolin, which exhibits antibacterial activity against *S.aureus* (Becker et al. 2020). Additionally, 2-(4-(dimethylamino)phenyl-4H-chromen-4-one, 1-naphthalenol, 4-methoxy-, and hexadecyl methanesulfonate, produced by *Hypoxylon* species, showed antibacterial activity against human pathogenic bacteria, including *B.subtilis*, *E.coli*, *S.aureus*, and *Pseudomonasaeruginosa* (Mishra et al. 2020). In this study, the new antibacterial compound 2-Naphthalenepropanol, 6-methoxy-α-methyl-, hydrogen sulfate was derived from *Hypoxylonthailandicum*.

Additionally, a few compounds with antibacterial properties were reported in the *Annulohypoxylonchiangraiense* (MFLUCC 24-0606) isolate in MEB medium, including phytosphingosine (C18H39NO3), pipemidic acid (C14H17N5O3), tetranactin (C44H72O12), and neomycin palmitate (C39H78N6O15). Phytosphingosine plays an important role in innate immune defense against epidermal and mucosal bacterial infections (Başpınar et al. 2018). Pipemidic acid is a derivative of piromidic acid and is active against Gram-negative and Gram-positive bacteria (Shimizu et al. 1975). Tetranactin exhibits antibacterial, insecticidal, and mitogenic properties and inhibits the growth of Gram-positive bacteria (Ando et al. 1971). Neomycin palmitate is active against both Gram-positive and Gram-negative organisms and mediates its pharmacological action by binding to bacterial ribosomes and inhibiting protein synthesis (National Center for Biotechnology Information 2025f).

Based on LC–QTOF–MS analyses, the *Hypoxylonthailandicum* (MFLUCC 25-0024) isolate in YMB recorded several compounds with antibacterial properties, including pentamidine (C19H24N4O2), phytosphingosine (C18H39NO3), tiamulin (C28H47NO4S), tobramycin (C18H37N5O9), and dibucaine (C20H29N3O2). Pentamidine exhibits antibacterial activity against Gram-negative bacteria (Stokes et al. 2017). Tiamulin is used as an antibacterial drug in veterinary medicine for the treatment of swine dysentery caused by *Serpulinahyodysenteriae* (National Center for Biotechnology Information 2025g), while dibucaine shows antibacterial activity against *Staphylococcusaureus* (Chakraborty et al. 2024).

The genus *Xylaria* is an important source of a variety of bioactive secondary metabolites, including terpenoids, nitrogen-containing compounds, polyketides, and lactones. These metabolites exhibit a range of biological activities, such as antimicrobial, anti-inflammatory, antifungal, cytotoxic, immunosuppressive, and enzyme-inhibitory activities (Chen et al. 2024). Among terpenoids, xylareremophil exhibits weak antibacterial activity against *Micrococcusluteus* and *Proteusvulgaris*. In triterpenoids, xylarioxides E and F show antibacterial activity against *Alternariaalternata*, *Curvularialunata*, and *Colletotrichumgloeosporioides*. Additionally, kolokosides A and xylarchalasins A display antibacterial activity against *B.subtilis*, *S.aureus*, and *E.coli*, respectively (Chen et al. 2024). This is the first time benzbromarone has been recorded as an antibacterial compound from *Xylariachrysanthum*.

In the genus *Daldinia*, most chemical investigations have focused on *D.concentrica* and *D.eschscholzii*, resulting in different kinds of chemical compounds. These include alkaloids, terpenoids, polyketides, polyphenols, and steroids, which exhibit antimicrobial, anti-inflammatory, antifungal, antiviral, cytotoxic, and enzyme-inhibitory activities (Yu et al. 2024). In alkaloids, dalesindoloids A and in chromones, 8-O-methylnodulisporin F and nodulisporin H showed antibacterial activity against *S.aureus*. In polyketides, 5-hydroxy-2-methoxy-6,7-dimethyl-1,4-naphthoquinone and fusaraisochromenone showed antibacterial activity against *Bacilluscereus* and *Enterococcusfaecalis*, *S.aureus*, *Escherichiacoli*, and *Pseudomonasaeruginosa*, respectively. Additionally, Daldisones B showed moderate antibacterial activities against *B.cereus*, *S.aureus*, and *Enterococcusfaecalis* (Yu et al. 2024). However, this is the first time linoleic acid has been recorded from *Daldiniaeschscholtzii*.

Investigation of new host and geographical records of fungi is important for understanding fungal-host interactions, disease management, monitoring biodiversity, identifying fungal distribution patterns, and revealing hidden fungal diversity (Rathnayaka et al. 2024). The documentation of five new species and nine new host records, including one geographical record within Xylariales, emphasizes the fungal diversity in these provinces in Thailand. This study has led to an expansion of the taxonomic framework of Xylariales as well as to the exploration of fungal diversity in Thailand within various types of dead twigs/branches in the forest ecosystem.

Most undiscovered sexual forms of xylarialean taxa are presumably inconspicuous forms and may be isolated as endophytes. The endophytic life cycle may account for the large numbers of species found in some of these genera (Bhunjun et al. 2022, 2024). However, there are few studies that have been conducted on inconspicuous forms compared to conspicuous forms (Daranagama et al. 2016). Due to the insufficient fresh fungal collection, the taxonomic studies on inconspicuous xylarialean taxa have been limited. Additionally, the genera transferred to new families were previously accepted, although they have uncertain morphologies and phylogenies (Samarakoon et al. 2023). Therefore, it is important to collect more fresh fungal samples to resolve the taxonomic placement of inconspicuous xylarialean taxa.

## Supplementary Material

XML Treatment for Astrocystis

XML Treatment for Astrocystis bambusae

XML Treatment for Annulohypoxylon

XML Treatment for Annulohypoxylon bahnphadengense

XML Treatment for Annulohypoxylon chiangraiense

XML Treatment for Annulohypoxylon crowfoothodgkiniae

XML Treatment for Annulohypoxylon spougei

XML Treatment for Annulohypoxylon purpureonitens

XML Treatment for Annulohypoxylon violaceopigmentum

XML Treatment for Halorosellinia

XML Treatment for Halorosellinia xylocarpi

XML Treatment for Stilbohypoxylon

XML Treatment for Stilbohypoxylon chiangraiense

XML Treatment for Hypoxylon

XML Treatment for Hypoxylon thailandicum

XML Treatment for Hypomontagnella

XML Treatment for Hypomontagnella hibisci

XML Treatment for Hypomontagnella monticulosa

XML Treatment for Diatrypella

XML Treatment for Diatrypella thailandica

XML Treatment for Paraeutypella

XML Treatment for Paraeutypella citricola

## ﻿Additional information

### Use of AI

No use of AI was reported.

### Author ORCIDs

Achala R. Rathnayaka https://orcid.org/0000-0001-8498-2715

K. W. Thilini Chethana https://orcid.org/0000-0002-5816-9269

Amuhenage T. Bhagya https://orcid.org/0009-0002-3988-298X

Hsan Win https://orcid.org/0009-0001-3830-7222

Zaw L. Tun https://orcid.org/0009-0001-3108-4425

Ausana Mapook https://orcid.org/0000-0001-7929-2429

Kevin D. Hyde https://orcid.org/0000-0002-2191-0762

### Data availability

All of the data that support the findings of this study are available in the main text.
